# Adiabatic Klein–Gordon dynamics for the renormalized Nelson model

**DOI:** 10.1007/s00526-026-03436-9

**Published:** 2026-10-12

**Authors:** Morris Brooks, David Mitrouskas

**Affiliations:** 1 Institut für Mathematik, Universität Zürich, Winterthurerstrasse 190, 8057 Zurich, Switzerland; 2 Institute of Science and Technology Austria (ISTA), Am Campus 1, 3400 Klosterneuburg, Austria

**Keywords:** 81Q05

## Abstract

We study the renormalized Nelson model in a semiclassical regime where the field becomes classical while the particle remains quantum. The degree of classicality is measured by a small parameter $\varepsilon \ll 1$. In this scaling the particle evolves on microscopic times, whereas the field exhibits nontrivial dynamics only on the macroscopic scale $t={\mathcal {O}}(\varepsilon ^{-2})$. The natural semiclassical model is the coupled Schrödinger–Klein–Gordon (SKG) system, which encodes the time-scale separation through an explicit ε-dependence. Based on this scale separation in SKG, we apply the adiabatic principle to derive a new PDE for the classical field, the ε-free adiabatic Klein–Gordon (aKG) equation, where the field is driven by the instantaneous ground state of the particle. Our main result is a norm approximation of the Nelson dynamics by the aKG solution corrected by a quasi-free fluctuation dynamics around the classical field, generated by a renormalized Bogoliubov–Nelson Hamiltonian. As a corollary, we obtain convergence of the reduced one-body densities for both subsystems, where the fluctuation correction vanishes, thereby justifying aKG as a semiclassical Born–Oppenheimer type approximation of the renormalized Nelson model.

## Introduction

The Nelson model describes nonrelativistic particles interacting with a quantized scalar boson field. Introduced by E. Nelson in 1964, it has long served as a benchmark in mathematical physics for studying ultraviolet divergences and renormalization at the Hamiltonian level, with motivations ranging from quantum electrodynamics to solid-state models. Beyond these aspects, it also provides a natural setting to investigate semiclassical limits where quantum matter interacts with emergent classical fields.

We investigate the single-particle, massless, renormalized Nelson model in a semiclassical regime where the quantized field behaves effectively classically, while the particle retains its quantum nature. The degree of classicality of the field is measured by a small parameter $\varepsilon \ll 1$, which can be viewed as an effective Planck constant. In this regime, the particle evolves on its intrinsic quantum scale $t={\mathcal {O}}(1)$, during which the field remains essentially frozen. In contrast, on macroscopic times $t={\mathcal {O}}(\varepsilon ^{-2})$, the dynamics of the classical field become visible. This scale separation between the fast particle and the slow field underlies our analysis and highlights the relevance of a long-time description.

The canonical semiclassical model of a quantum particle coupled to a classical field is the Schrödinger–Klein–Gordon (SKG) system,

1.1 $$\begin{aligned} i \partial _t \psi _t (x)&\; =\; \varepsilon ^{-2} \left( - \Delta \;+\; 2 \text {Re}\int _{{\mathbb {R}}^3} \frac{e^{-ikx}}{\sqrt{|k|}}\, \varphi _t(k)\, \text {d}k \right) \psi _t(x) , \end{aligned}$$

1.2 $$\begin{aligned} i \partial _t \varphi _t(k)&\; =\; |k|\, \varphi _t(k) \;+\; \int _{{\mathbb {R}}^3} \frac{e^{-ikx}}{\sqrt{|k|}}\,|\psi _t(x)|^2 \,\text {d}x . \end{aligned}$$

The first equation describes the fast quantum particle in the external potential generated by the field, while the second is a Klein–Gordon equation for the slow classical field driven by the particle density. The SKG system still carries an explicit ε-dependence, which encodes the time-scale separation between the particle and the field. This dependence on the semiclassical parameter, however, also makes SKG somewhat inconvenient as a semiclassical approximation of the full quantum model.

For initial data where the particle is prepared in the gapped ground state of the potential generated by the classical field, one can exploit the adiabatic principle to obtain an ε-free semiclassical description. The idea is to eliminate the fast particle by letting it adiabatically follow the instantaneous ground state associated with the field. This yields an ε-independent, closed evolution for the field alone, the *adiabatic Klein–Gordon (aKG) equation*,

1.3 $$\begin{aligned} i\partial _t\varphi _t(k) \;=\; |k|\,\varphi _t(k) \;+\; \int _{{\mathbb {R}}^3} \frac{e^{-ikx}}{\sqrt{|k|}}\,|\psi _{\varphi _t}(x)|^2 \,\text {d}x, \end{aligned}$$

where $\psi _{\varphi _t}$ denotes the ground state of the Schrödinger operator with external potential generated by $\varphi _t$. The aKG equation governs the autonomous evolution of the field on the macroscopic time scale, and can be viewed as a field-theoretic Born–Oppenheimer type approximation of SKG. To our knowledge, Eq. (1.3) has not been studied before. We establish its local well-posedness and prove that SKG converges to aKG as $\varepsilon \rightarrow 0$. A related adiabatic limit has been studied for the Landau–Pekar equations, which play the role of the SKG equations in the Fröhlich polaron model. In [12], this limit is analyzed in a one-dimensional setting and leads to an adiabatic version of the Landau–Pekar equations, analogous to (1.3). While the strategy in [12] relies on adiabatic dispersive estimates for time-dependent Schrödinger operators, our approach is more direct: inspired by ideas from [30], we compare $\psi _t$ directly with the instantaneous ground state $\psi _{\varphi _t}$, where $(\psi _t,\varphi _t)$ is a solution of SKG. This comparison is based on the persistence of the spectral gap along the aKG flow.

Our main result is a quantitative norm approximation of the renormalized Nelson dynamics, valid for suitable initial states up to times $t={\mathcal {O}}(\varepsilon ^{-2})$. The effective description is given by the aKG pair $(\psi _{\varphi _t}, \varphi _t)$, corrected by quantum fluctuations around the classical field. These fluctuations are governed by a *quadratic* (Bogoliubov) Hamiltonian and hence generate a *quasi-free* evolution on Fock space. The naive quadratic generator, however, inherits the logarithmic ultraviolet divergence of the Nelson Hamiltonian, and one of the key ingredients is the renormalization of this operator, which we call the *Bogoliubov–Nelson Hamiltonian*.

As a consequence of the norm approximation, we obtain convergence of the reduced one-body densities for both particle and field. At this level the fluctuation correction disappears, thereby justifying aKG as a semiclassical Born–Oppenheimer type approximation of the renormalized Nelson model. Moreover, through convergence of SKG to aKG as $\varepsilon \rightarrow 0$, our results directly imply corresponding approximations in terms of the (ε-dependent) SKG solutions.

The remainder of this introduction is devoted to precise formulations of the relevant models: the renormalized Nelson Hamiltonian, the adiabatic Klein–Gordon equation, and the renormalized quantum fluctuation dynamics around the aKG field.

### The renormalized Nelson Hamiltonian

We consider a single particle coupled to a quantized scalar field. The Hilbert space is

1.4 $$\begin{aligned} {\mathscr {H}} = L^2({\mathbb {R}}^3_x) \otimes {\mathcal {F}}, \end{aligned}$$

where $L^2({\mathbb {R}}^3_x)$ denotes the particle space with position variable *x*, and ${\mathcal {F}}:= \Gamma _{\textrm{sym}}(L^2({\mathbb {R}}^3_k))$ is the bosonic Fock space constructed over the one-particle space with momentum variable *k*. We write $a_k^*$ and $a_k$ for the creation and annihilation operators satisfying the usual bosonic commutation relations $[a_k,a_\ell ^*]=\delta (k-\ell )$ and $[a_k,a_\ell ]=[a_k^*,a_\ell ^*]=0$. The free particle and field Hamiltonians are

1.5 $$\begin{aligned} p^2:= - \Delta , \qquad H_f:=\int _{{\mathbb {R}}^3}\omega (k)\,a_k^*a_k\,dk, \qquad \omega (k):=|k|. \end{aligned}$$

Formally, the massless Nelson Hamiltonian with semiclassical parameter $\varepsilon \ll 1$ is given by the expression

1.6 $$\begin{aligned} p^2 \;+\; \varepsilon \, \int _{{\mathbb {R}}^3} \Big ( {{\textbf {G}}}(x,k) a_k^* + \overline{ {{\textbf {G}}}(x,k) } a_k \Big ) \text {d}k \;+ \; \varepsilon ^2 H_f, \end{aligned}$$

acting on $L^2({\mathbb {R}}^3_x) \otimes {\mathcal {F}}$, with *x*-dependent kernel

1.7 $$\begin{aligned} {\textbf{G}}(x,k):=e^{-ikx} \, \omega (k)^{-1/2}. \end{aligned}$$

Note that the powers of ε match the number of creation and annihilation operators in each term, which shows that $\varepsilon \rightarrow 0$ corresponds to a semiclassical limit.

Since $\omega ^{-1} G \notin L^2({\mathbb {R}}^3_k)$, the interaction is not form-bounded with respect to free Hamiltonian $p^2 + H_f$, and the formal Hamiltonian (1.6) is UV singular, hence neither self-adjoint nor associated with a closed quadratic form.

Introducing a cutoff $\Lambda \in (0,\infty )$ and

1.8 $$\begin{aligned} {\textbf{G}}_\Lambda (x,k):= {{\textbf {G}}}(x,k)\, {\textbf{1}}_{ |k|\leqslant \Lambda } . \end{aligned}$$

we define the regularized Nelson Hamiltonian

1.9 $$\begin{aligned} H_\Lambda ^\varepsilon \; = \; p^2 \;+\; \varepsilon \, \int _{{\mathbb {R}}^3} \Big ( {{\textbf {G}}}_\Lambda (x,k) a_k^* + \overline{ {{\textbf {G}}}_\Lambda (x,k) } a_k \Big ) \text {d}k \;+ \; \varepsilon ^2 H_f, \end{aligned}$$

which is self-adjoint and semibounded with quadratic form domain $Q(H_\Lambda ^\varepsilon ) = Q(p^2+H_f)$ for all $\Lambda \in (0,\infty )$.

The following proposition shows that, up to a diverging energy shift, the regularized operators converge as $\Lambda \rightarrow \infty$. We call the limit operator $H^\varepsilon$ the *renormalized Nelson Hamiltonian* in the semiclassical scaling.

#### Proposition 1.1

For every ε>0 there is a self-adjoint, semibounded operator $H^\varepsilon$ such that

1.10 $$\begin{aligned} \exp \big ( -i t H^\varepsilon _\Lambda \big ) \exp \big ( -i t 4\pi \varepsilon ^2 \ln \Lambda \big ) \xrightarrow [\Lambda \rightarrow \infty ]{} \exp \big (-i t H^{\varepsilon } \big ) \end{aligned}$$

for every $t\in {\mathbb {R}}$, in the strong sense on $L^2({\mathbb {R}}^3_x) \otimes {\mathcal {F}}$.

This was first established in [35] and later refined in [16]. The method is based on a unitary dressing transformation, which will also play a central role in our analysis. For completeness, we include a proof of Proposition 1.1 in Appendix A, where we adapt the ideas of [16] to the semiclassical setting. Alternative approaches to renormalization of the Nelson model rely on functional integration [17] or interior boundary conditions [23]. Finally, we note the drastic effect of renormalization on the domain: as shown in [16, 23], the form domains intersect only trivially, that is $Q(H^\varepsilon )\cap Q(H^\varepsilon _\Lambda )=\{0\}$ for all $\Lambda \in (0,\infty )$.

### The adiabatic Klein–Gordon equation

The semiclassical model associated with the Nelson Hamiltonian is obtained by replacing the creation and annihilation operators in (1.6) with a classical field $\varepsilon ^{-1}\varphi \in L^2({\mathbb {R}}^3_k)$. Taking the expectation value in a normalized particle state $\psi \in H^1({\mathbb {R}}^3_x)$ then yields the ε-independent energy functional

1.11 $$\begin{aligned} {\mathcal {E}}(\psi , \varphi ) = \int \text {d}x \, |\nabla \psi (x) |^2 + \int \text {d}k \, \omega (k) |\varphi (k)|^2 + \iint \text {d}k \text {d}x \, | \psi (x)|^2 \frac{2 \text {Re}(e^{-ikx} \varphi (k) ) }{\sqrt{\omega (k)}}. \end{aligned}$$

Unlike the quantum model, the semiclassical energy is ultraviolet regular. Its Hamiltonian flow gives the Schrödinger–Klein–Gordon (SKG) system for $(\psi _t,\varphi _t)\in L^2({\mathbb {R}}^3_x)\times L^2({\mathbb {R}}^3_k)$,

SKG $$\begin{aligned} {\left\{ \begin{array}{ll} \begin{aligned} & \ i \partial _t \psi _t = h_{\varphi _t} \psi _t \\ & \ i \varepsilon ^{-2} \partial _t \varphi _t = \omega \varphi _t + \sigma ( \psi _t ) \end{aligned} \end{array}\right. } \end{aligned}$$

with Schrödinger operator

1.12 $$\begin{aligned} h_{\varphi _t} = p^2 + V_{\varphi _t} \qquad \text {with} \qquad V_{\varphi _t}(x) = 2 \text {Re}\langle \varphi _t , {{\textbf {G}}}(x,\cdot ) \big \rangle \end{aligned}$$

and source term

1.13 $$\begin{aligned} \sigma ( \psi _t ) (k) = \langle \psi _t, {{\textbf {G}}} (\cdot , k) \psi _t\rangle \end{aligned}$$

where ${{\textbf {G}}}$ is given by (1.7).

Note that (SKG) is equivalent to (1.1)–(1.2) after rescaling time by $\varepsilon ^{-2}$. A key feature of the SKG system is its explicit ε-dependence: although the semiclassical energy is ε-free, the underlying Poisson/commutator structure forces the field to evolve on the slow (macroscopic) time scale $t = {\mathcal {O}}( \varepsilon ^{-2})$, while the particle evolves on the fast (microscopic) scale $t = {\mathcal {O}}( 1 )$. Thus the SKG solutions depend explicitly on ε, which makes them less convenient as a semiclassical approximation of the Nelson dynamics in the limit $\varepsilon \rightarrow 0$. This motivates the formulation of an ε-free effective description.

To achieve this, we exploit the explicit time-scale separation in (SKG): inspired by the adiabatic principle, we eliminate the fast particle by letting it adiabatically follow the instantaneous ground state associated with the field. This leads to a closed, ε-independent evolution for the field alone, which we call the *adiabatic Klein–Gordon* (aKG) equation,

aKG $$\begin{aligned} i \partial _t \varphi _t \;=\; \omega \varphi _t \; +\; \sigma (\psi _{\varphi _t}), \end{aligned}$$

where $\psi _{\varphi _t}\geqslant 0$ denotes the normalized ground state of $h_{\varphi _t}=p^2+V_{\varphi _t}$. The structure of aKG mirrors the classical Born–Oppenheimer approximation: the fast quantum particle adiabatically follows its instantaneous ground state, while the slow field evolves autonomously. This is analogous to molecular Born–Oppenheimer theory, where the fast electrons follow the electronic ground state determined by the nuclear configuration, and the heavy nuclei evolve on their intrinsic slow time scale; see [18, 36, 38].

Before continuing, we introduce the energy spaces for the particle and the field

1.14 $$\begin{aligned} H^r({\mathbb {R}}^3_x)&:= \big \{ \psi \in L^2({\mathbb {R}}^3_x) : \Vert (1-\Delta )^{r/2} \psi \Vert _{L^2} < \infty \big \}, \quad&r\in {\mathbb {R}}, \end{aligned}$$

1.15 $$\begin{aligned} {\mathfrak {h}}_s&:= \big \{ \varphi \in L^2({\mathbb {R}}^3_k) : \Vert \omega ^s \varphi \Vert _{L^2} < \infty \big \}, \quad&s\in {\mathbb {R}}. \end{aligned}$$

For brevity, we write $H^r:= H^r({\mathbb {R}}^3_x)$. These spaces will be used throughout.

For all applications that follow, we consider initial data where the particle is in the gapped ground state of the Schrödinger operator $h_\varphi =p^2+V_\varphi$. To this end, we assume that

1.16 $$\begin{aligned} e(\varphi )\;:=\;\inf \sigma (h_\varphi )\;<\;0, \end{aligned}$$

which guarantees that $h_\varphi$ has a unique ground state $\psi _\varphi \in H^2({\mathbb {R}}^3)$. Without loss of generality, we may take $\psi _\varphi \geqslant 0$. We also define the spectral gap

1.17 $$\begin{aligned} \triangle (\varphi ) \,=\, \inf \big \{\,|e(\varphi )-\lambda | : \lambda \in \sigma (h_\varphi )\setminus \{e(\varphi )\}\,\big \}, \end{aligned}$$

that is, the distance between the ground state energy e(φ) and the rest of the spectrum.

Let $I\subset {\mathbb {R}}$ be an interval with 0∈I. We say that $\varphi \in C(I;{\mathfrak {h}}_{1/2})$ is a solution to (aKG) on the interval *I* with initial datum $\varphi _0\in {\mathfrak {h}}_{1/2}$, if $e(\varphi _t)<0$ for all t∈I and

1.18 $$\begin{aligned} \varphi _t \;=\; e^{-i\omega t}\varphi _0 \;-\; i\int _0^t e^{-i\omega (t-s)}\,\sigma (\psi _{\varphi _s})\, \text {d}s. \end{aligned}$$

The existence of the ground state $\psi _{\varphi _t}$ for all t∈I follows from the condition $e(\varphi _t)<0$.

The next lemma establishes local well-posedness of the aKG equation.

#### Proposition 1.2

Let $\varphi _0\in {\mathfrak {h}}_{1/2}$ with $e(\varphi _0)<0$. There exist maximal times $T_-, T_+ \in (0,\infty ]$ such that (aKG) admits a unique solution

1.19 $$\begin{aligned} \varphi \in C((-T_-,T_+ );{\mathfrak {h}}_{1/2}) \ \cap \ C^1((-T_-,T_+);{\mathfrak {h}}_{-1/2}). \end{aligned}$$

In particular, we have $e(\varphi _t)<0$ for all $t\in (-T_-,T_+ )$, and therefore $h_{\varphi _t}$ has a unique ground state $\psi _{\varphi _t}$ for every such *t*. Moreover, for any compact interval $J \subset (-T_-,T_+)$ the spectral gap remains uniformly positive,

1.20 $$\begin{aligned} \inf _{ t \in J }\triangle (\varphi _t) \;>\; 0. \end{aligned}$$

If in addition $\varphi _0\in {\mathfrak {h}}_0\cap {\mathfrak {h}}_{1/2}$, then $\varphi \in C((-T_-, T_+);{\mathfrak {h}}_0)$.

Finally, we either have $T_{+} = \infty$, or else $T_{+}<\infty$ and $\liminf _{ t \rightarrow T_{+}} \triangle (\varphi _t) = 0$, and equivalently for $T_-$.

The proof is given in Sect. 5. In general, global well-posedness cannot be expected, since the spectral gap $\triangle (\varphi _t)$ may close at a finite time. Once the gap vanishes, the ground state map $\varphi _t \mapsto \psi _{\varphi _t}$ is typically no longer well defined; moreover, continuation of the local solution fails in the absence of a positive gap.

Conversely, if the spectral gap remains uniformly positive for all times, then the solution extends globally. This is in particular the case for stationary solutions, which are the minimizers of the classical field functional

1.21 $$\begin{aligned} {\mathcal {E}}_{\textrm{field}}(u) := e(u) + \Vert u\Vert _{{\mathfrak {h}}_{1/2}}^2 . \end{aligned}$$

These minimizers are known to be unique up to spatial translations [31]. We denote by $\varphi _*$ a fixed minimizer, and by

1.22 $$\begin{aligned} {\mathcal {M}} := \{\,T_y \varphi _* : y\in {\mathbb {R}}^3\}, \qquad (T_y u)(k):=e^{-ik y}u(k), \end{aligned}$$

the manifold of all minimizers. For $u\in {\mathfrak {h}}_{1/2}$ we define the distance to ${\mathcal {M}}$ by

1.23 $$\begin{aligned} \textrm{dist}_{{\mathfrak {h}}_{1/2}}(u,{\mathcal {M}}) := \inf _{y\in {\mathbb {R}}^3} \Vert u-T_y\varphi _*\Vert _{{\mathfrak {h}}_{1/2}}. \end{aligned}$$

In the next lemma, we prove that for initial data that is sufficiently close to ${\mathcal {M}}$, the spectral gap remains uniformly positive along the evolution. As a consequence, the corresponding aKG solution exists for all times. The proof of the lemma is postponed to Appendix B.

#### Lemma 1.3

There exists η>0 such that for all initial data $\varphi _0\in {\mathfrak {h}}_{1/2}$ with $\textrm{dist}_{{\mathfrak {h}}_{1/2}}(\varphi _0,{\mathcal {M}}) \;\leqslant \; \eta$, the aKG solution $\varphi _t$ extends globally in time and satisfies

1.24 $$\begin{aligned} \inf _{t\in {\mathbb {R}}} \triangle (\varphi _t) \, > \, 0. \end{aligned}$$

Even though aKG is an equation for the field alone, we will often refer to $(\psi _{\varphi _t},\varphi _t)$ as the aKG solution pair. The main goal of this work is to show that this pair provides an effective description of the renormalized Nelson dynamics in the limit $\varepsilon \rightarrow 0$. At the same time, it is instructive to make the connection between SKG and aKG explicit. In Appendix C, we therefore show that the SKG solution $(\psi _t^\varepsilon ,\varphi _t^\varepsilon )$, when observed on the rescaled slow time scale, converges to the aKG pair $(\psi _{\varphi _t},\varphi _t)$. More precisely, on compact intervals in $(-T_-,T_+)$,

1.25 $$\begin{aligned} \exp \left( i \varepsilon ^{-2} \int _0^t e \left( \varphi ^\varepsilon _{\varepsilon ^{-2}s} \right) \text {d}s \right) \, \psi _{\varepsilon ^{-2} t }^\varepsilon \xrightarrow [\varepsilon \rightarrow 0]{} \psi _{\varphi _t} \qquad \text {and}\qquad \varphi _{\varepsilon ^{-2} t }^\varepsilon \xrightarrow [\varepsilon \rightarrow 0]{} \varphi _t \end{aligned}$$

both in $L^2$-norm, where $e(\varphi _s^\varepsilon ):=\inf \sigma (h_{\varphi _s^\varepsilon })$. Although this result is not used in our proofs, it makes the adiabatic picture precise and may be of independent interest. In particular, it allows us to translate the main results of Sect. 2 into SKG-based approximations.

### The renormalized quantum fluctuations

Let $\varphi _t$ denote the solution to (aKG) with initial condition $\varphi _0 \in {\mathfrak {h}}_{1/2}$ satisfying $e(\varphi _0)<0$, and let $\psi _{\varphi _t}\geqslant 0$ be the instantaneous ground state of $h_{\varphi _t}$. By Proposition 1.2 there exists T>0 such that $h_{\varphi _t}-e(\varphi _t)$ has a uniform spectral gap above zero for all |t|⩽T. In particular, we can define the reduced resolvent on $L^2({\mathbb {R}}^3_x)$, given by

1.26 $$\begin{aligned} R_{\varphi _t} := Q_{\varphi _t}\,( h_{\varphi _t}-e(\varphi _t))^{-1}\,Q_{\varphi _t}, \qquad Q_{\varphi _t}={\textbf{1}}-P_{\varphi _t},\quad P_{\varphi _t}=|\psi _{\varphi _t}\rangle \langle \psi _{\varphi _t}|, \end{aligned}$$

which is a bounded operator for all |t|⩽T thanks to the spectral gap.

For the regularized Nelson Hamiltonian (1.9) with UV cutoff $\Lambda \in (0,\infty )$, the evolution of the quantum fluctuations is generated by the time-dependent quadratic (Bogoliubov) Hamiltonian acting on Fock space ${\mathcal {F}}$,

1.27 $$\begin{aligned} {\mathbb {H}}_\Lambda (t)\;=\;H_f\;- \iint \, \textrm{d}k\, \text {d}\ell \, \langle \psi _{\varphi _t},{{\textbf {G}}}_\Lambda (\cdot , k) R_{\varphi _t} {{\textbf {G}}}_\Lambda (\cdot ,\ell ),\psi _{\varphi _t}\rangle \, \big (a_k^*+a_{-k}\big )\big (a_\ell ^*+a_{-\ell }\big ) \end{aligned}$$

where $H_f=\textrm{d}\Gamma (\omega )$ is the free-field energy and $\langle \cdot , \cdot \rangle$ denotes the inner product in $L^2({\mathbb {R}}^3_x)$. The form of this operator can be understood from second-order perturbation theory around the adiabatic subspace ${\mathcal {V}}(t):=\{\psi _{\varphi _t}\otimes \Theta :\Theta \in {\mathcal {F}}\}$. At a formal level, after applying the Weyl shift associated with the classical field, it arises as the quadratic contribution at order $\varepsilon ^2$ in the Feshbach–Schur complement of the Weyl-shifted Hamiltonian $H_\Lambda ^\varepsilon$ with respect to the decomposition ${\mathcal {V}}(t)\oplus {\mathcal {V}}(t)^\perp$.

We denote by ${\mathbb {U}}_\Lambda (t)$ the unitary propagator on ${\mathcal {F}}$ generated by ${\mathbb {H}}_\Lambda (t)$ with initial time t=0. The next result establishes the existence of a renormalized Bogoliubov evolution in the limit $\Lambda \rightarrow \infty$; in particular, it shows that ${\mathbb {H}}_\Lambda (t)$ exhibits the same logarithmic divergence as the original Nelson Hamiltonian.

#### Proposition 1.4

Let $\varphi _0\in {\mathfrak {h}}_{1/2}$ with $e(\varphi _0)<0$, and let $\varphi _t$ denote the solution to (aKG) with initial datum $\varphi _0$. There exists T>0 such that the following statements hold:

(i) For every $\Lambda \in [2,\infty )$ and $\Psi \in D(H_f^{1/2})$ there exists a unique solution $\Psi \in C([-T,T],{\mathcal {F}})\cap L^\infty ([-T,T],D(H_f^{1/2}))$ to the Cauchy problem
  1.28 $$\begin{aligned} {\left\{ \begin{array}{ll} \begin{aligned} & i\partial _t \Psi (t) = {\mathbb {H}}_\Lambda (t)\,\Psi (t),\\ & \Psi (0) = \Psi , \end{aligned} \end{array}\right. } \end{aligned}$$
  with the equation understood in $D((1+H_f)^{-1/2})$. The map $\Psi \mapsto \Psi (t)$ defines a unitary ${\mathbb {U}}_\Lambda (t)$ on ${\mathcal {F}}$ that is strongly continuous in *t*.
(ii) For all |t|⩽T, the strong limit
  1.29 $$\begin{aligned} {\mathbb {U}}(t)\;:=\;s - \lim _{\Lambda \rightarrow \infty }\,{\mathbb {U}}_\Lambda (t)\,\exp \big (-it\,4\pi \ln \Lambda \big ) \end{aligned}$$
  exists, is unitary, and the map $t\mapsto {\mathbb {U}}(t)$ is strongly continuous.

The proposition is proved in Sect. 6. We call ${\mathbb {U}}(t)$ the *renormalized Bogoliubov–Nelson evolution* on ${\mathcal {F}}$. While we do not explicitly use the following property in our analysis, one can show that ${\mathbb {U}}(t)$ acts as a Bogoliubov transformation on Fock space, i.e., it implements a linear rotation of creation and annihilation operators. In particular, ${\mathbb {U}}(t)$ preserves the Gaussian (Wick factorization) property of quasi-free states in ${\mathcal {F}}$.

## Main results

We consider *semiclassical product initial data* of the form

2.1 $$\begin{aligned} \Psi ^\varepsilon (\varphi _0) \;=\; \psi _{\varphi _0}\ \otimes \ W(\varepsilon ^{-1}\varphi _0)\,\Omega , \end{aligned}$$

where $\psi _{\varphi _0}\geqslant 0$ is the normalized ground state of $h_{\varphi _0}=p^2+V_{\varphi _0}$ and $\Omega = (1, {{\textbf {0}}})$ denotes the Fock vacuum in ${\mathcal {F}}$. Here

2.2 $$\begin{aligned} W(f):=\exp \!\big (a^*(f)-a(f)\big ) \end{aligned}$$

is the Weyl operator, and thus the coherent state $W(\varepsilon ^{-1}\varphi _0)\Omega$ represents the classical field $\varepsilon ^{-1}\varphi _0$ with ${\mathcal {O}}(\varepsilon ^{-2})$ excitations.

From a physical perspective, such states arise naturally in experimental situations: one first *imposes* a classical field $\varphi _0$ (e.g. by external driving) and keeps it fixed until the particle is cooled into the ground state $\psi _{\varphi _0}$ of the frozen Hamiltonian $h_{\varphi _0}=p^2+V_{\varphi _0}$. At time t=0 the external control is removed and the system evolves autonomously under the renormalized Nelson Hamiltonian, with the initial state prepared in $\Psi ^\varepsilon (\varphi _0)$. Unless the stationary condition $\omega \varphi _0+\sigma (\psi _{\varphi _0})=0$ is satisfied, this preparation constitutes a quench from a frozen to a non-stationary system, initiating nontrivial coupled dynamics on the macroscopic time scale.

Our main theorem gives a norm approximation for the renormalized Nelson evolution for initial states of the form (2.1). As corollaries, we obtain convergence of the one-body reduced densities for both subsystems.

### Norm approximation

The following theorem is the main result of this work.

#### Theorem 2.1

Let $\varphi _0\in {\mathfrak {h}}_0\cap {\mathfrak {h}}_{1/2}$ with $e(\varphi _0)<0$, and let $\varphi _t$ be the solution to (aKG) with initial datum $\varphi _0$. Let $\psi _{\varphi _t}\geqslant 0$ denote the normalized ground state of $h_{\varphi _t}=p^2+V_{\varphi _t}$, and set

2.3 $$\begin{aligned} \mu (t)=\int _0^t\Big (\text {Im}\langle \varphi _s,\partial _s \varphi _s\rangle +\Vert \omega ^{1/2}\varphi _s\Vert _{L^2}^2+e(\varphi _s)\Big )\,\textrm{d}s . \end{aligned}$$

Moreover, let $H^\varepsilon$ be the renormalized Nelson Hamiltonian from Proposition 1.1, and let ${\mathbb {U}}(t)$ be the renormalized Bogoliubov–Nelson evolution from Proposition 1.4 (defined in terms of $\varphi _t$). Then there exist constants $C,T,\varepsilon _0>0$, depending only on $\varphi _0$, such that

$$\begin{aligned} \Big \Vert \exp \!\left( -i \varepsilon ^{-2} t H^\varepsilon + i \varepsilon ^{-2}\mu (t)\right) \,(\psi _{\varphi _0}\otimes W(\varepsilon ^{-1}\varphi _0)\Omega ) - \psi _{\varphi _t}\otimes W(\varepsilon ^{-1}\varphi _t)\,{\mathbb {U}}(t)\Omega \Big \Vert \;\leqslant \; C \,\varepsilon ^{\tfrac{1}{10}} \ln \tfrac{1}{\varepsilon } \end{aligned}$$

for all |t|⩽T and $\varepsilon \in (0,\varepsilon _0]$.

This result provides a norm approximation of the renormalized Nelson dynamics up to times $t={\mathcal {O}}(\varepsilon ^{-2})$, in terms of a semiclassical state built from the aKG pair $(\psi _{\varphi _t},\varphi _t)$ and corrected by the fluctuation dynamics ${\mathbb {U}}(t)$ around the coherent aKG field. The theorem is formulated for |t|≤T (corresponding to physical times $|t|\le \varepsilon ^{-2}T$), where *T* is tied to the existence of the aKG solution and the persistence of the associated spectral gap; see Proposition 1.2. In general, *T* may have to be chosen small. By Lemma 5.1, however, if the initial datum is sufficiently close to the manifold ${\mathcal {M}}$ of field minimizers, the aKG solution is global and the gap stays uniformly positive; in this case one can choose *T* arbitrarily large, so the approximation holds on any finite macroscopic interval.

### Convergence of reduced densities

As corollaries of Theorem 2.1, we derive convergence of the reduced one-body densities.

We start with the particle, whose reduced density at time *t* is defined by

2.4 $$\begin{aligned} \gamma _{\textrm{particle}}^\varepsilon (t) := \operatorname {Tr}_{{\mathcal {F}}}\Big ( e^{-i \varepsilon ^{-2} t H^\varepsilon } |\Psi ^\varepsilon (\varphi _0)\rangle \langle \Psi ^\varepsilon (\varphi _0)| e^{i \varepsilon ^{-2} t H^\varepsilon } \Big ), \end{aligned}$$

where $\Psi ^\varepsilon (\varphi _0)$ is the initial state from (2.1).

#### Corollary 2.2

Let $\varphi _0\in {\mathfrak {h}}_0 \cap {\mathfrak {h}}_{1/2}$ with $e(\varphi _0)<0$, and let $\varphi _t$ be the solution to (aKG) with initial datum $\varphi _0$. Let $\psi _{\varphi _t}\geqslant 0$ denote the normalized ground state of $h_{\varphi _t}=p^2+V_{\varphi _t}$. Then there exist constants $C,T,\varepsilon _0>0$, depending only on $\varphi _0$, such that

2.5 $$\begin{aligned} \operatorname {Tr}_{L^2}\Big |\,\gamma _{\textrm{particle}}^\varepsilon (t)-|\psi _{\varphi _t}\rangle \langle \psi _{\varphi _t}|\,\Big | \;\leqslant \; C\,\varepsilon ^{\tfrac{1}{10}}\ln \tfrac{1}{\varepsilon } \end{aligned}$$

for all |t|⩽T and $\varepsilon \in (0,\varepsilon _0]$.

To analyze the reduced one-body density of the field we start from a dressed initial state. In our proof this step is required, since we need an energy estimate for the initial state. The undressed product states $\Psi ^\varepsilon (\varphi _0)$ are not in the form domain of $H^\varepsilon$ and therefore have infinite energy, whereas the dressed state introduced below has finite energy.

We therefore introduce the unitary dressing transformation

2.6 $$\begin{aligned} U_K^\varepsilon \;:=\; \exp \!\left( \varepsilon \int \text {d}k \Big ( {{\textbf {B}}}_K(x,k) a_k^* - \overline{ {\boldsymbol{B}}_K(x,k)} a_k \Big ) \text {d}k \right) \end{aligned}$$

with

2.7 $$\begin{aligned} {\boldsymbol{B}}_K(x,k) := \frac{{{\textbf {G}}}(x,k)}{k^2}\,{\textbf{1}}_{|k|\geqslant K}. \end{aligned}$$

Accordingly, we define the dressed initial state as

2.8 $$\begin{aligned} \Psi _{K}^\varepsilon (\varphi _0) \;:=\; W(\varepsilon ^{-1} \varphi _0)\, (U_K^\varepsilon )^* \,(\psi _{\varphi _0} \otimes \Omega ). \end{aligned}$$

The difference between the dressed state $\Psi _{K}^\varepsilon (\varphi _0)$ and the undressed state $\Psi ^\varepsilon (\varphi _0)$ is negligible in norm as $\varepsilon \rightarrow 0$ or $K\rightarrow \infty$:

2.9 $$\begin{aligned} \Vert \Psi _{K}^\varepsilon (\varphi _0) - \Psi ^\varepsilon (\varphi _0) \Vert \;\leqslant \; C \varepsilon K^{-1}, \end{aligned}$$

so that Theorem 2.1 continues to hold with the dressed initial state. This bound follows from the standard estimate

2.10 $$\begin{aligned} \Vert \big ( U_K^\varepsilon - {\textbf{1}} \big )\Psi \Vert \;\leqslant \; C\varepsilon \Vert B_K\Vert _{L^2} \Vert (N+1)^{1/2} \Psi \Vert , \end{aligned}$$

valid for every $\Psi \in D(N^{1/2})$, together with $\Vert B_K \Vert _{L^2} \leqslant C K^{-1}$. Here *N* denotes the number operator on ${\mathcal {F}}$,

2.11 $$\begin{aligned} N := \int _{{\mathbb {R}}^3} a_k^*a_k \, \text {d}k . \end{aligned}$$

With this modification, we define the field’s reduced one-body density for the dressed initial state, through its kernel

2.12 $$\begin{aligned} \gamma _{\textrm{field}}^\varepsilon (t)(k,\ell ) := \varepsilon ^2\, \big \langle e^{-i\varepsilon ^{-2}t H^\varepsilon }\Psi _{K}^\varepsilon (\varphi _0),\ a_k^*a_\ell \, e^{-i\varepsilon ^{-2}t H^\varepsilon }\Psi _{K}^\varepsilon (\varphi _0) \big \rangle . \end{aligned}$$

The pre-factor ensures the normalization

2.13 $$\begin{aligned} \lim _{\varepsilon \rightarrow 0} \operatorname {Tr}_{L^2}\big (\gamma _{\textrm{field}}^\varepsilon (0)\big ) = \Vert \varphi _0\Vert _{L^2}^2. \end{aligned}$$

Additionally, corresponding to the dressed initial state $\Psi _{K}^\varepsilon (\varphi _0)$, we define the reduced density of the electron as

$$\begin{aligned} {\widetilde{\gamma }}_{\textrm{particle}}^\varepsilon (t) := \operatorname {Tr}_{{\mathcal {F}}}\Big ( e^{-i \varepsilon ^{-2} t H^\varepsilon } |\Psi ^\varepsilon _K(\varphi _0)\rangle \langle \Psi ^\varepsilon _K(\varphi _0)| e^{i \varepsilon ^{-2} t H^\varepsilon } \Big ). \end{aligned}$$

In principle, the parameter *K* could be left free in the next corollary, leading to additional error terms that vanish as $K \rightarrow \infty$. To balance the errors and simplify the statements, we fix $K=\varepsilon ^{-4/5}$.

#### Corollary 2.3

Let $\varphi _0\in {\mathfrak {h}}_0 \cap {\mathfrak {h}}_{1/2}$ with $e(\varphi _0)<0$, and let $\varphi _t$ be the solution to (aKG) with initial datum $\varphi _0$. For $K=\varepsilon ^{-4/5}$, choose $\Psi _K^\varepsilon (\varphi _0)$ as in (2.8). Then there exist constants $C,T,\varepsilon _0>0$, depending only on $\varphi _0$, such that

2.14 $$\begin{aligned} \Big \Vert \,N^{1/2}\,W(\varepsilon ^{-1}\varphi _t)^*\,e^{-i\,\varepsilon ^{-2} t H^\varepsilon }\Psi _{K}^\varepsilon (\varphi _0) \Big \Vert ^2&\;\leqslant \; C \, \varepsilon ^{-29/15} \ln \tfrac{1}{\varepsilon } \end{aligned}$$

2.15 $$\begin{aligned} \operatorname {Tr}_{L^2}\Big |\, \gamma _{\textrm{field}}^\varepsilon (t)-|\varphi _t\rangle \langle \varphi _t|\,\Big |&\;\leqslant \; C\,\varepsilon ^{1/15} \ln \tfrac{1}{\varepsilon } \end{aligned}$$

for all |t|⩽T and $\varepsilon \in (0,\varepsilon _0]$. Furthermore,

2.16 $$\begin{aligned} \operatorname {Tr}_{L^2}\Big |\,{\widetilde{\gamma }}_{\textrm{particle}}^\varepsilon (t)-|\psi _{\varphi _t}\rangle \langle \psi _{\varphi _t}|\,\Big | \;\leqslant \; C\,\varepsilon ^{\tfrac{1}{10}}\ln \tfrac{1}{\varepsilon } \end{aligned}$$

for all |t|⩽T and $\varepsilon \in (0,\varepsilon _0]$.

The estimate (2.14) shows that the field component of $e^{-i\varepsilon ^{-2} t H^\varepsilon }\Psi _{K}^\varepsilon (\varphi _0)$ remains close to the coherent state $W(\varepsilon ^{-1}\varphi _t)\Omega$, in the sense that the number of non-coherent excitations is negligible compared to the ${\mathcal {O}}(\varepsilon ^{-2})$ coherent excitations as $\varepsilon \rightarrow 0$. The trace-norm bound (2.15) then yields convergence of the field’s one-body reduced density to the rank-one projector $|\varphi _t\rangle \langle \varphi _t|$. Taken together, (2.5), (2.14) and (2.15) show that the Nelson dynamics is accurately described at the level of reduced densities by the aKG equation. While the quasi-free fluctuation dynamics ${\mathbb {U}}(t)$ is essential for the norm approximation, it does not affect the reduced observables.

### Comparison with the literature

The present work lies at the intersection of partially classical limits for quantum field models and strong-coupling polaron dynamics. We highlight three closely related works and sketch the relevant similarities and differences.

*Strong-coupling Fröhlich polaron dynamics.* Our analysis is inspired by recent works on the Fröhlich polaron at large coupling [27, 30, 34]; see also [11, 13, 15] for earlier contributions. Setting $\alpha ^{-1} = \varepsilon$ identifies the strong-coupling limit $\alpha \rightarrow \infty$ with a semiclassical limit for the phonon field, and the macroscopic time scale $t = {\mathcal {O}}( \alpha ^2)$ corresponds to our time scale $t = {\mathcal {O}}( \varepsilon ^{-2})$. The main difference from the Nelson model is that the Fröhlich Hamiltonian is UV-regular. In [27], a *norm approximation* of the dynamics was established up to times $t = {\mathcal {O}}( \alpha ^2 )$ by the Landau–Pekar (LP) system, corrected by a Bogoliubov (quasi-free) fluctuation dynamics around the coherent state. The α-dependent LP system is the analogue of (SKG) for the polaron.

In the renormalized Nelson model, the problem is more delicate. Most importantly, the fluctuation generator is UV-divergent and requires a renormalization procedure, a complication that is absent in the Fröhlich case. In addition, we introduce and analyze the aKG equation, which provides an ε-free effective dynamics, whereas both LP and SKG depend explicitly on the semiclassical parameter. These two ingredients, renormalization of the fluctuation generator and the use of aKG, constitute the conceptual novelties of this work. At the same time, our analysis builds on and extends the adiabatic expansion scheme developed for the polaron, by adapting it to the more singular setting of the Nelson model.

*Partially classical limit for the renormalized Nelson model.* Ginibre, Nironi and Velo [14] gave the first rigorous analysis of the renormalized Nelson model in a partially classical regime. They worked on microscopic time scales $t= {\mathcal {O}}( 1 )$ and used a coherent-state/weak-coupling scaling, where the number of field excitations is of order $\varepsilon ^{-2}$ and the coupling strength is proportional to ε. In this regime the quantum field converges to a solution of the free wave (Klein–Gordon) equation, and the particles evolve by a Schrödinger equation with an external potential generated by the free field. Since the back-reaction on the field is suppressed, the effective dynamics remains linear. The renormalized Hamiltonian is constructed via a unitary dressing transformation, and convergence of the propagators is obtained on a suitable subspace, with a quantitative error estimate.

In our scaling the situation is different: the Poisson/commutator structure places a factor $\varepsilon ^{-2}$ in front of $\partial _t \varphi$, so at microscopic times $t= {\mathcal {O}}( 1)$ the field remains frozen rather than evolving freely. Nontrivial field evolution appears only on the slow scale $t = {\mathcal {O}}( \varepsilon ^{-2} )$. On that scale we establish a norm approximation in terms of the nonlinear aKG equation, corrected by a renormalized fluctuation dynamics. The need to renormalize the fluctuation generator has no counterpart in [14], since the field there remains free and unaffected by back-reaction and fluctuations.

*Quasi-classical/Wigner-measure approach.* In [5] the authors developed an abstract quasi-classical framework for particle-field systems and applied it to several models, see also [4], including the UV-regularized Nelson model. The quasi-classical parameter is introduced by scaling creation and annihilation operators with a factor ε,^1^ together with an ε-dependent weight $\nu (\varepsilon )$ in front of the field energy (our setting corresponds to $\nu (\varepsilon )=1$). At microscopic times $t = {\mathcal {O}}( 1 )$ they distinguish two regimes: if $\varepsilon \nu (\varepsilon ) \rightarrow 0$ the classical field remains frozen, while if $\varepsilon \nu (\varepsilon ) \rightarrow 1$ it evolves freely. Since there is no back-reaction on the field on this time scale, the effective particle dynamics is linear, driven by an external classical field. Convergence to the effective evolution is established via state-valued Wigner measures. Their assumptions on initial states are very general and allow for entangled particle-field configurations.

Our analysis addresses the renormalized Nelson model for more specific, semiclassical initial data and focuses on the macroscopic time scale. In this regime the field exhibits nontrivial back-reaction and the effective dynamics is nonlinear.

*Other works.* Within the broader context of deriving effective equations from particle-field models, let us also mention the following works. In [1, 7–9, 24–26, 28, 29], different particle-field models were studied in a many-body mean-field regime, where *N* bosons couple weakly to the quantum field with coupling strength of order $N^{-1/2}$. In particular, [1, 8] treat the renormalized Nelson model and derive the SKG system as an effective description. In this setting there is no separation of time scales between the bosons and the field, and hence ε=1. Another difference to our semiclassical setting is that the quantum fluctuations in the mean-field regime act simultaneously on the excitations of the bosons and the field, which requires a different renormalization strategy [8]. Finally, in the mean-field limit, the norm approximation of the dynamics is obtained as a consequence of the convergence of reduced one-body densities, whereas in our setting we first prove the norm approximation and deduce the reduced densities as a corollary.

The works [3, 6, 17, 21, 39, 40] study the derivation of effective (direct) particle interactions arising from the coupling to Nelson type quantum fields. These results are obtained in suitable weak-coupling and adiabatic regimes, where the roles of time scales are reversed: the particles evolve slowly while the field provides the fast subsystem.

### Outline of the paper

We start in Sect. 3 with a collection of auxiliary estimates for creation and annihilation operators and for the classical potentials. These bounds are used throughout Sects. 4–8.

Section 4 introduces the dressed Nelson Hamiltonian, obtained by applying the unitary dressing transformation $U_K^\varepsilon$, see (2.6), to the renormalized Nelson Hamiltonian. We then perform a Weyl shift around the classical field, which separates the leading particle Hamiltonian from subleading field terms and expands the Hamiltonian in powers of ε. The purpose of this section is to obtain a suitable representation of the Nelson Hamiltonian that is needed in the proof of the norm approximation.

In Sect. 5 we analyze the (aKG) equation: we prove local well-posedness (by a contraction argument), energy conservation and persistence of the spectral gap for suitable time intervals. Beyond well-posedness, this provides control on resolvents and regularity estimates for the aKG pair $(\psi _{\varphi _t},\varphi _t)$, which are repeatedly used later on.

In Sect. 6 we construct the renormalized Bogoliubov–Nelson evolution ${\mathbb {U}}(t)$ as the strong limit of the regularized propagators generated by ${\mathbb {H}}_{\Lambda }(t)$ (defined in (1.27)), up to a logarithmically diverging energy counter term. This renormalization is based on the key identity

2.17 $$\begin{aligned} {\mathbb {H}}_\Lambda (t) = {\mathbb {H}}_{{\mathscr {D}},K,\Lambda }(t) - 4\pi \ln \Lambda \end{aligned}$$

where the operator ${\mathbb {H}}_{{\mathscr {D}},K,\Lambda }(t)$ is related to the dressed Nelson Hamiltonian and has a limit as $\Lambda \rightarrow \infty$. In contrast to the full quantum model, this renormalization does not rely on the application of a unitary dressing transformation. We also establish bounds for powers of the number operator propagated under ${\mathbb {U}}(t)$.

Section 7 is devoted to the proof of Theorem 2.1. After applying the unitary dressing transformation and a coherent shift aligned with the aKG solution, we compare the renormalized Nelson dynamics with the semiclassical evolution associated with $(\psi _{\varphi _t},\varphi _t)$, corrected by the fluctuation dynamics ${\mathbb {U}}(t)$. The comparison relies on a first-order Duhamel expansion combined with a second- and third-order adiabatic expansion around the instantaneous gapped particle ground state. A crucial step in this expansion is a cancellation coming from the generator ${\mathbb {U}}(t)$, which removes a contribution of order one on the macroscopic time scale $t={\mathcal {O}}(\varepsilon ^{-2})$. After this cancellation, all remainder terms are of higher order in ε and can be controlled using the estimates established in the preceding sections.

In Sect. 8 we deduce convergence of the reduced densities. While the convergence of the reduced particle density follows immediately from the norm approximation, the analysis of the reduced one-body density of the field requires additional work. Here we combine the norm approximation with an improved energy estimate and an a priori bound on the propagated number operator.

Finally, Appendix A revisits the renormalization of the Nelson model in the semiclassical regime and establishes the dressing identity for the Nelson Hamiltonian. Appendix B extends the local well-posedness of aKG to global well-posedness for initial data close to ${\mathcal {M}}$, using energy conservation together with a coercive lower bound for the field energy. Appendix C proves the convergence of SKG to aKG as $\varepsilon \rightarrow 0$.

## Auxiliary estimates

### Definitions and notation

Before turning to the technical estimates, we summarize notation that will be used repeatedly throughout this and the following sections.

First, we introduce *x*-dependent creation and annihilation operators acting on $L^2({\mathbb {R}}^3_x)\otimes {\mathcal {F}}$ by

3.1 $$\begin{aligned} (a^*({\textbf{f}})\Psi )(x):=\left( \int _{{\mathbb {R}}^3} {\textbf{f}}(x,k)\,a_k^*\, dk \right) \Psi (x), \quad (a({\textbf{f}})\Psi )(x):=\left( \int _{{\mathbb {R}}^3} \overline{{\textbf{f}}(x,k)}\,a_k \, dk \right) \Psi (x), \end{aligned}$$

for kernels ${\textbf{f}}:{\mathbb {R}}^3_x\times {\mathbb {R}}^3_k\rightarrow {\mathbb {C}}$ that are measurable in *x* and satisfy $\omega ^{-1/2}{\textbf{f}}(x,\cdot )\in L^2({\mathbb {R}}^3_k)$ for almost every $x\in {\mathbb {R}}^3$. Under this condition, the operators are well defined as quadratic forms on $Q(H_f)$. When ${\textbf{f}}(x,k)=e^{-ik x}f(k)$ for suitable $f:{\mathbb {R}}^3_k\rightarrow {\mathbb {C}}$, we keep the boldface notation for the *x*-dependent kernel and write *f* for the *k*-only kernel. We further set

3.2 $$\begin{aligned} \phi ({\textbf{f}}) := a^*({\textbf{f}}) + a({\textbf{f}}). \end{aligned}$$

Next, we recall the kernels already defined in the previous sections,

3.3 $$\begin{aligned} {\boldsymbol{G}}_K(x,k)&= e^{-ikx}\, G_K(k)&\qquad&\text {with} \qquad&G_K(k)&= \omega (k)^{-1/2}\, {\textbf{1}}_{|k|\leqslant K}, \end{aligned}$$

3.4 $$\begin{aligned} {\boldsymbol{B}}_K(x,k)&= e^{-ikx}\, B_K(k)&\qquad&\text {with} \qquad&B_K(k)&= \frac{G(k)}{|k|^2}\, {\textbf{1}}_{|k|\geqslant K}. \end{aligned}$$

With an additional ultraviolet cutoff $\Lambda \geqslant K$, we write

3.5 $$\begin{aligned} {\boldsymbol{B}}_{K,\Lambda }(x,k) := e^{-ikx} B_{K,\Lambda }(k), \qquad B_{K,\Lambda }(k) := B_K(k)\, {\textbf{1}}_{|k|\leqslant \Lambda }. \end{aligned}$$

For K⩾0 we define the modified number and field energy operators

3.6 $$\begin{aligned} N_K := \int {\textbf{1}}_{|k|\geqslant K}\, a_k^* a_k \, \text {d}k, \qquad H_{f,K} := \int {\textbf{1}}_{|k|\geqslant K}\, \omega (k)\, a_k^* a_k \, \text {d}k, \end{aligned}$$

so that $N=N_0$ and $H_f=H_{f,0}$.

For any densely defined operator *A* we write

3.7 $$\begin{aligned} \langle A\rangle := (1+A^*A)^{1/2}, \end{aligned}$$

and we use

3.8 $$\begin{aligned} X \lesssim Y \end{aligned}$$

to denote an estimate of the form $X \leqslant C\,Y$ for some constant C>0 independent of the relevant parameters (e.g. ε, *K*, and Λ). The value of *C* may change from line to line.

For ease of reference we recall the momentum operator and the dispersion relation

3.9 $$\begin{aligned} p:=-i\nabla _x, \qquad \omega (k):=|k|, \end{aligned}$$

as well as the energy spaces for the particle and the classical field:

3.10 $$\begin{aligned} H^r&:= \big \{ \psi \in L^2({\mathbb {R}}^3_x) : \Vert (1+p^2)^{r/2} \psi \Vert _{L^2} < \infty \big \}, \quad&r\in {\mathbb {R}}, \end{aligned}$$

3.11 $$\begin{aligned} {\mathfrak {h}}_s&:= \big \{ \varphi \in L^2({\mathbb {R}}^3_k) : \Vert \omega ^s \varphi \Vert _{L^2} < \infty \big \}, \quad&s\in {\mathbb {R}}. \end{aligned}$$

Finally, for the Fourier transform we use the convention

3.12 $$\begin{aligned} {\widehat{f}}(k) := \int _{{\mathbb {R}}^3} e^{-ikx} f(x)\,\text {d}x, \qquad f(x) = (2\pi )^{-3}\int _{{\mathbb {R}}^3} e^{ikx}\, {\widehat{f}}(k)\,\text {d}k. \end{aligned}$$

### Bounds for creation and annihilation operators

We begin with standard operator bounds for the creation and annihilation operators (3.1). These are simple consequences of the CCR; see e.g. [16, Lemma B.1] and [3, Lemma 3.3].

#### Lemma 3.1

Let ${{\textbf {f}}}(x,k) = e^{-ikx} f(k)$ with $f:{\mathbb {R}}^3 \rightarrow {\mathbb {R}}$ measurable and $\textrm{supp}(f) \subseteq \{ |k| \geqslant K \}$ for K⩾0. Then

3.13 $$\begin{aligned} \Vert a({{\textbf {f}}} ) \Psi \Vert&\le \Vert f\Vert _{L^2} \Vert N_K ^{ \frac{1}{2}} \Psi \Vert , \qquad&\Vert a({{\textbf {f}}} ) \Psi \Vert&\le \Vert \omega ^{ - \frac{1}{2}} f\Vert _{L^2} \Vert H_{f,K}^{ \frac{1}{2} }\Psi \Vert , \end{aligned}$$

3.14 $$\begin{aligned} \Vert a^*({{\textbf {f}}}) \Psi \Vert&\le \Vert f \Vert _{L^2} \Vert \langle N_K ^{ \frac{1}{2} }\rangle \Psi \Vert , \qquad&\Vert a^*({{\textbf {f}}} ) \Psi \Vert&\le \max \{ \Vert f \Vert _{L^2} , \Vert \omega ^{ - \frac{1}{2}} f\Vert _{L^2} \} \Vert \langle H_{f,K}^{ \frac{1}{2} }\rangle \Psi \Vert \end{aligned}$$

for all $\Psi \in L^2({\mathbb {R}}^3_x) \otimes {\mathcal {F}}$. Additionally, for ${{\textbf {g}}}(x,k) = e^{-ikx} g(k)$ with $g:{\mathbb {R}}^3 \rightarrow {\mathbb {R}}$ measurable and $\textrm{supp}(g) \subseteq \{ |k| \geqslant \Lambda \}$ for $\Lambda \geqslant K$, we have

3.15 $$\begin{aligned} \Vert a({{\textbf {f}}}) a({{\textbf {g}}}) \Psi \Vert&\le \Vert \omega ^{ - \frac{1}{4}} f\Vert _{L^2} \Vert \omega ^{ - \frac{1}{4}} g\Vert _{L^2} \, \Vert \langle N_K^{\frac{1}{2}} \rangle H_{f,K} ^{\frac{1}{2}} \Psi \Vert . \end{aligned}$$

The next lemma is a generalized version of the Frank–Schlein estimate [13, Lemma 7].

#### Lemma 3.2

Let ${{\textbf {f}}} (x,k) = e^{-ikx} f(k)$ with $f:{\mathbb {R}}^3 \rightarrow {\mathbb {R}}$ measurable. Then

$$\begin{aligned} \Vert a( {{\textbf {f}}} ) \Psi \Vert ^2 \leqslant {\mathscr {C}}_{s,r}(f) \, \Vert \langle |p|^r \rangle \text {d}\Gamma (\omega ^s)^{\frac{1}{2}} \Psi \Vert ^2 \quad \text {with}\quad {\mathscr {C}}_{s,r}(f) = \sup _{h \in {\mathbb {R}}^3} \int \text {d}k \frac{|f(k)|^2}{\omega (k)^s(1+ |h-k|^{2r}) } \end{aligned}$$

for all $\Psi \in L^2({\mathbb {R}}^3_x) \otimes {\mathcal {F}}$ and s,r⩾0, where $\text {d}\Gamma (\omega ^s) = \int \omega (k)^s a_k^* a_k dk$.

Moreover, if k↦f(k) is radial and monotone decreasing, then

3.16 $$\begin{aligned} {\mathscr {C}}_{s,r}(f) \leqslant \int \text {d}k \frac{|f(k)|^2}{ \omega (k)^s ( 1 + |k|^{2r} ) } \ . \end{aligned}$$

#### Proof

Set $f_s = \omega (k)^{-\frac{s}{2}}f$ and estimate via Cauchy–Schwarz:

3.17 $$\begin{aligned} \Vert a( {{\textbf {f}}} ) \Psi \Vert ^2&= \int \text {d}k \text {d}\ell \Big \langle e^{i k x } \overline{f_s(k)} \omega (k)^{\frac{s}{2}} a_k \Psi , \frac{ \langle |p - k|^r \rangle }{ \langle |p - \ell |^r \rangle } \frac{ \langle |p - \ell |^r \rangle }{ \langle |p - k |^r \rangle } e^{i\ell x} \overline{f_s( \ell )}\omega (\ell )^{\frac{s}{2}} a_\ell \Psi \Big \rangle \nonumber \\&\leqslant \int \text {d}k \text {d}\ell \Big \Vert \frac{1}{\langle |p - k|^r \rangle } f_s(k ) \langle | p - \ell |^r \rangle e^{i\ell x} \omega (\ell )^{\frac{s}{2}} a_\ell \Psi \Big \Vert ^2 \nonumber \\&= \int \text {d}\ell \, \omega (\ell )^{s}\Big \langle \langle |p - \ell |^r \rangle e^{i\ell x} a_\ell \Psi , \int dk \frac{|f_s(k)|^2}{ \langle |p - k|^r \rangle ^2} \langle |p - \ell |^r \rangle e^{i\ell x} a_\ell \Psi \Big \rangle \nonumber \\&\leqslant \bigg \Vert \int \text {d}k \frac{|f_s(k)|^2}{\langle |p - k|^r \rangle ^2} \bigg \Vert \, \Vert \langle |p|^r \rangle \text {d}\Gamma (\omega ^s )^\frac{1}{2} \Psi \Vert ^2 \ , \end{aligned}$$

where we used the identity $\langle | p-\ell |^r\rangle e^{i\ell x } = e^{i \ell x } \langle |p|^r \rangle$ in the last step. It remains to estimate the operator norm, that is, for normalized $\psi \in L^2({\mathbb {R}}^3)$

3.18 $$\begin{aligned} \bigg \Vert \int \text {d}k \frac{ |f_s(k)|^2}{\langle |p- k|^r \rangle ^2 } \psi \bigg \Vert&= \bigg [ \int \text {d}p \bigg | \int \text {d}k \frac{ |f_s(k)|^2}{\langle |p- k|^r \rangle ^2 } {\widehat{\psi }}(p) \bigg |^2 \bigg ]^{\frac{1}{2}} \leqslant \sup _{p\in {\mathbb {R}}^3} \int \text {d}k \frac{ |f_s(k)|^2}{ 1+ | p - k| ^{2r} } . \end{aligned}$$

This completes the proof of the first statement.

The second statement is a consequence of the symmetric rearrangement inequality. □

We now specialize these bounds to the kernels (3.3) and (3.4). For reference, let us note the following bounds:

3.19a $$\begin{aligned}&\Vert \omega ^{- s } G_K \Vert _{L^2} \lesssim K^{1-s}, \end{aligned}$$

3.19b $$\begin{aligned}&\Vert {\textbf{1}}_{|k| \geqslant 2 } |k|^{-1} G_K \Vert _{L^2} \lesssim \sqrt{\ln K} , \end{aligned}$$

3.19c $$\begin{aligned}&\Vert \omega ^{s-1} k B_K \Vert _{ L^2} \lesssim K^{s-1} \ , \end{aligned}$$

for all K⩾2 and s∈[0,1).

The next lemma collects useful estimates on the constant ${\mathscr {C}}_{r,s}(f)$.

#### Lemma 3.3

Let ${\mathscr {C}}_{r,s}(\cdot )$ be defined as in Lemma 3.2. For all K⩾2, $s\in \{0,1\}$ and r∈(0,1],

3.20 $$\begin{aligned} {\mathscr {C}}_{0,1}(G_K)&\lesssim \ln K ,&\qquad \qquad \qquad {\mathscr {C}}_{0,r}(kB_K)&\lesssim K^{-3r/2} , \end{aligned}$$

3.21 $$\begin{aligned} {\mathscr {C}}_{1,1}(G_K)&\lesssim 1 ,&{\mathscr {C}}_{1,1}(\omega k B_K)&\lesssim K^{-3/4} , \end{aligned}$$

3.22 $$\begin{aligned} {\mathscr {C}}_{s,1}(\omega G_K)&\lesssim K^{2-s} ,&{\mathscr {C}}_{0,2}(\omega k B_K)&\lesssim K^{-1} . \end{aligned}$$

#### Proof

The bounds involving $G_K$ are readily verified via (3.16). For the bounds involving $B_K$, where *K* enters as a lower cut-off, we use

3.23 $$\begin{aligned} {\mathscr {C}}_{0,r}(kB_K)&= \sup _{h\in {\mathbb {R}}^3} \int \frac{{\textbf{1}}(|k|\geqslant K) }{\omega (k) |k|^2 (1+ |k-h|^{2r})} \text {d}k \nonumber \\&\leqslant K^{- \frac{3}{2} r} \sup _{h\in {\mathbb {R}}^3} \int \frac{|k|^{\frac{3}{2} r}{\textbf{1}}(|k|\geqslant K) }{\omega (k)|k|^{2}(1+ |k-h|^{2r})}\text {d}k \lesssim K^{-\frac{3}{2} r }, \end{aligned}$$

where the last step follows by removing the restriction |k|⩾K and then applying (3.16). The other estimates follow analogously. □

We combine Lemmas 3.1–3.3 to obtain the following estimates.

#### Lemma 3.4

For all K⩾2 and $\Psi \in L^2({\mathbb {R}}^3_x) \otimes {\mathcal {F}}$, we have

3.24a $$\begin{aligned}&\Vert a ( {{\textbf {G}}}_K) \Psi \Vert \, \lesssim \, \min \big \{ \, ( \ln K )^{\frac{1}{2}} \Vert \langle p\rangle N^{\frac{1}{2}} \Psi \Vert \, , \, \Vert \langle p \rangle H_f^\frac{1}{2} \Psi \Vert \, , \, K^\frac{1}{2} \Vert H_f^\frac{1}{2} \Psi \Vert \big \} , \end{aligned}$$

3.24b $$\begin{aligned}&\Vert a (\omega {{\textbf {G}}}_K) \Psi \Vert \, \lesssim \, \min \big \{ \, K \, \Vert \langle p\rangle N^\frac{1}{2} \Psi \Vert \, , \, K^{\frac{1}{2}} \Vert \langle p\rangle H_f^\frac{1}{2} \Psi \Vert \, \big \} , \end{aligned}$$

3.24c $$\begin{aligned}&\Vert a(k {{\textbf {B}}}_K) \Psi \Vert \, \lesssim \, \min \big \{ \, K^{-\frac{3}{4}} \Vert \langle p \rangle N^{\frac{1}{2}} \Psi \Vert \, , \, K^{-\frac{1}{2}} \Vert H_f^{\frac{1}{2}} \Psi \Vert \, \big \}, \end{aligned}$$

3.24d $$\begin{aligned}&\Vert a(\omega k {{\textbf {B}}}_K) \Psi \Vert \, \lesssim \, \min \big \{ K^{-\frac{3}{8}} \Vert \langle p \rangle H_f^{\frac{1}{2}} \Psi \Vert \, ,\, K^{-\frac{1}{2}} \Vert \langle p^2 \rangle N^\frac{1}{2} \Psi \Vert \big \} . \end{aligned}$$

Additionally, for all $\Lambda \geqslant K \geqslant 2$:

3.24e $$\begin{aligned}&| \langle \Psi , a(k {{\textbf {B}}}_K) a (k {{\textbf {B}}}_\Lambda ) \Phi \rangle | \, \lesssim \, (K \Lambda )^{-\frac{1}{4}} \, \Vert \langle H_f^{\frac{1}{2}}\rangle \Psi \Vert \Vert \langle H_f^{\frac{1}{2}}\rangle \Phi \Vert , \end{aligned}$$

3.24f $$\begin{aligned}&\Vert a(k {{\textbf {B}}}_K) a (k {{\textbf {B}}}_\Lambda ) \Psi \Vert \, \lesssim \, (K \Lambda )^{-\frac{3}{8}} \, \Vert \langle p \rangle N \Psi \Vert \, \ . \end{aligned}$$

#### Proof

The bounds (3.24a) and (3.24b) follow from Lemmas 3.2 and 3.3. The second bound in (3.24c) follows directly from Lemma 3.1 and (3.19c). The first bound in (3.24c) and both bounds in (3.24d) follow from Lemma 3.2 together with (3.19c).

For the bound in (3.24e), we apply Cauchy–Schwarz, Lemma 3.1, and (3.19c):

3.25 $$\begin{aligned} | \langle \Psi , a( k {{\textbf {B}}}_K ) a( k {{\textbf {B}}}_\Lambda ) \Phi \rangle |&= | \langle \Psi , a( k {{\textbf {B}}}_K ) a( k {{\textbf {B}}} _\Lambda ) \langle N_K ^{\frac{1}{2}}\rangle \langle N_K^{\frac{1}{2}}\rangle ^{-1} \Phi \rangle | \nonumber \\&= | \langle (N_K + 3)^{\frac{1}{2}} \Psi , a( k {{\textbf {B}}}_K ) a( k {{\textbf {B}}} _\Lambda ) \langle N_K^{\frac{1}{2}}\rangle ^{-1} \Phi \rangle | \nonumber \\&\lesssim \Vert \langle N_K^{\frac{1}{2}} \rangle \Psi \Vert \, \Vert a( k {{\textbf {B}}}_K ) a( k{{\textbf {B}}}_\Lambda ) \langle N_K^{\frac{1}{2}}\rangle ^{-1} \Phi \Vert \nonumber \\&\lesssim \Vert \omega ^{ - \frac{1}{4}} k B_K \Vert _{L^2} \, \Vert \omega ^{ - \frac{1}{4}} k B_\Lambda \Vert _{L^2} \, \Vert \langle N_K^{\frac{1}{2}} \rangle \Psi \Vert \, \Vert H_{f,K}^\frac{1}{2} \Phi \Vert \nonumber \\&\lesssim (K\Lambda )^{-\frac{1}{4}} \, \Vert \langle H_f^{\frac{1}{2}} \rangle \Phi \Vert \, \Vert H_f^\frac{1}{2} \Psi \Vert , \end{aligned}$$

where we used $N_K \leqslant H_{f,K}\leqslant H_f$ for K⩾2. For the bound in (3.24f), we apply Lemma 3.2 twice, that is, we estimate

3.26 $$\begin{aligned} \Vert a(k {{\textbf {B}}}_K) a( k {{\textbf {B}}}_\Lambda ) \Psi \Vert ^2&\leqslant {\mathscr {C}}_{0,\frac{1}{2}}(k B_K) \, \Vert \langle |p|^\frac{1}{2} \rangle a (k {{\textbf {B}}}_\Lambda ) N^\frac{1}{2} \Psi \Vert ^2 \end{aligned}$$

and

3.27 $$\begin{aligned} \Vert \langle |p|^\frac{1}{2} \rangle a (k {{\textbf {B}}}_\Lambda ) N^\frac{1}{2} \Psi \Vert ^2&\leqslant 2\, \Vert a ( \langle |k|^\frac{1}{2} \rangle kB_\Lambda ) N^\frac{1}{2} \Psi \Vert ^2 + \, 2 \, \Vert a (k{{\textbf {B}}}_\Lambda ) \langle |p|^\frac{1}{2} \rangle N^\frac{1}{2} \Psi \Vert ^2 \nonumber \\&\leqslant 2\, {\mathscr {C}}_{0,1} (|k|^\frac{3}{2} B_\Lambda ) \, \Vert \langle p\rangle N \Psi \Vert ^2 + 2\, {\mathscr {C}}_{0,\frac{1}{2}} (kB_\Lambda ) \, \Vert \langle |p|^\frac{1}{2}\rangle ^2 N \Psi \Vert ^2 . \end{aligned}$$

Using $\langle |p|^{1/2}\rangle ^2 \lesssim \langle p\rangle$ and Lemma 3.2 concludes the proof of (3.24e); note that ${\mathscr {C}}_{0,1}(|k|^{3/2} B_\Lambda ) = {\mathscr {C}}_{1,1}(\omega k B_\Lambda )$, since ω(k)=|k|. □

### Bounds for $A_K$ and $D_K$

We now apply the previous estimates to control operators that will appear repeatedly in the upcoming sections. To this end, let us define

3.28 $$\begin{aligned} A_{K}&:= \phi ( {{\textbf {G}}}_K ) + 2 a^*( k {{\textbf {B}}}_{K } ) \cdot p + 2 p \cdot a ( k {{\textbf {B}}}_{K} ), \end{aligned}$$

3.29 $$\begin{aligned} D_{K}&:= 2 a^*(k {{\textbf {B}}}_{K } ) a( k {{\textbf {B}}}_{K} ) + a^*( k {{\textbf {B}}}_{K} )^2 + a( k {{\textbf {B}}}_{K}) ^2 + 4\pi \ln K \end{aligned}$$

The estimates summarized next play a central role in the construction of the renormalized fluctuation dynamics in Sect. 6. We provide bounds for $A_K$ and $D_K$ both in terms of the number operator *N* and the field energy $H_f$.

#### Lemma 3.5

Let R⩾0 be a bounded non-negative operator acting on $L^2({\mathbb {R}}^3_x)$. For all K⩾2 and $\Psi \in L^2({\mathbb {R}}^3_x) \otimes {\mathcal {F}}$ we have

3.30a $$\begin{aligned}&|\langle \Psi , A_{K} R A_{K} \Psi \rangle | \lesssim K^2 \big (\Vert \langle p \rangle R \langle p \rangle \Vert + \Vert R \Vert \big ) \Vert \langle H_f^\frac{1}{2} \rangle \langle p \rangle \Psi \Vert ^2 , \end{aligned}$$

3.30b $$\begin{aligned}&|\langle \Psi , [N,A_{K}] R A_{K} \Psi \rangle | \lesssim K^2 \big (\Vert \langle p \rangle R \langle p \rangle \Vert + \Vert R \Vert \big ) \Vert \langle H_f^\frac{1}{2} \rangle \langle p \rangle \Psi \Vert ^2 , \end{aligned}$$

3.30c $$\begin{aligned}&|\langle \Psi , D_{K} \Psi \rangle | \lesssim K^{-\frac{1}{2}} \Vert \langle H_f^\frac{1}{2} \rangle \Psi \Vert ^2 + \ln K \Vert \Psi \Vert ^2 , \end{aligned}$$

3.30d $$\begin{aligned}&|\langle \Psi , [N,D_{K}] \Psi \rangle | \lesssim K^{-\frac{1}{2}} \Vert \langle H_f^\frac{1}{2} \rangle \Psi \Vert ^2 . \end{aligned}$$

Moreover, in terms of the number operator *N* we have

3.31a $$\begin{aligned}&|\langle \Psi , A_K R A_{K} \Psi \rangle | \lesssim \ln K \big (\Vert \langle p \rangle R \langle p \rangle \Vert + \Vert R \Vert \big ) \Vert \langle N^\frac{1}{2} \rangle \langle p \rangle \Psi \Vert ^2 , \end{aligned}$$

3.31b $$\begin{aligned}&|\langle \Psi , [N , A_K ] R A_{K} \Psi \rangle | \lesssim \ln K \big (\Vert \langle p \rangle R \langle p \rangle \Vert + \Vert R \Vert \big ) \Vert \langle N^\frac{1}{2} \rangle \langle p \rangle \Psi \Vert ^2 , \end{aligned}$$

3.31c $$\begin{aligned}&|\langle \Psi , D_K \Psi \rangle | \lesssim \ln K \Vert \langle N^\frac{1}{2} \rangle \langle p \rangle \Psi \Vert ^2 , \end{aligned}$$

3.31d $$\begin{aligned}&|\langle \Psi , [N , D_K ] \Psi \rangle | \lesssim K^{-\frac{1}{2}} \Vert \langle N^\frac{1}{2} \rangle \langle p \rangle \Psi \Vert ^2 . \end{aligned}$$

#### Proof

Since R⩾0, it suffices to bound the diagonal terms in $A_K R A_K$. With the aid of Lemma 3.1 and $\Vert G_K\Vert _{L^2} \lesssim K$, we find

3.32 $$\begin{aligned} |\langle \Psi , \phi ({{\textbf {G}}}_K) R \phi ({{\textbf {G}}}_K) \Psi \rangle |&\lesssim K^2 \Vert R \Vert \, \Vert \langle H_f^\frac{1}{2} \rangle \Psi \Vert ^2 , \end{aligned}$$

and invoking Lemma 3.4, we also get

3.33 $$\begin{aligned} |\langle \Psi , (a^*(k{{\textbf {B}}}_K) \cdot p) R (p\cdot a (k {{\textbf {B}}}_K)) \Psi \rangle |&\lesssim K^{-1} \Vert \langle p \rangle R \langle p \rangle \Vert \, \Vert \langle H_f^\frac{1}{2} \rangle \Psi \Vert ^2 . \end{aligned}$$

For the third diagonal term in $A_K R A_K$, we apply Cauchy–Schwarz and then Lemma 3.2 (with s=0, r=1) and Lemma 3.3:

3.34 $$\begin{aligned} \langle \Psi , (p\cdot a(k {{\textbf {B}}}_K)) R (a^* (k {{\textbf {B}}}_K) \cdot p ) \Psi \rangle&= | \langle (N_K+2)^{\frac{1}{2}} \Psi , (p \cdot a(k {{\textbf {B}}}_K)) \langle N_K^\frac{1}{2} \rangle ^{-1} R (a^* (k {{\textbf {B}}}_K) \cdot p) \Psi \rangle | \nonumber \\&\lesssim \Vert p \langle N_K^\frac{1}{2} \rangle \Psi \Vert \, \Vert a(k {{\textbf {B}}}_K) \langle N_K^\frac{1}{2} \rangle ^{-1} R (a^* (k {{\textbf {B}}}_K) \cdot p) \Psi \Vert \nonumber \\&\lesssim {\mathscr {C}}_{0,1}(kB_K)^\frac{1}{2} \, \Vert p \langle N_K^\frac{1}{2} \rangle \Psi \Vert \, \Vert R a^* (k{{\textbf {B}}}_K) \cdot p \Psi \Vert \nonumber \\&\lesssim K^{-\frac{1}{2}} \, \Vert R^{\frac{1}{2}} \Vert \, \Vert \langle p\rangle \langle N_K^\frac{1}{2}\rangle \Psi \Vert \, \Vert R^{\frac{1}{2}} a^* (k{{\textbf {B}}}_K)\cdot p \Psi \Vert . \end{aligned}$$

Dividing by the last factor, and using $N_K\leqslant H_f$ for K⩾1 gives

3.35 $$\begin{aligned} \langle \Psi , (p\cdot a(k {{\textbf {B}}}_K) ) R ( a^* (k {{\textbf {B}}}_K) \cdot p ) \Psi \rangle&\lesssim K^{-1} \Vert R \Vert \, \Vert \langle p\rangle \langle H_f^\frac{1}{2}\rangle \Psi \Vert ^2. \end{aligned}$$

Combining (3.32)–(3.35) proves (3.30a); the commutator bound (3.30b) follows in the same way. The two bounds for $D_{K}$ are immediate consequences of Lemma 3.4.

To show (3.31a), we estimate again the diagonal terms in $A_K R A_K$. By Lemmas 3.2 and 3.3,

3.36 $$\begin{aligned} |\langle \Psi , a^* ({{\textbf {G}}}_K) R a ({{\textbf {G}}}_K) \Psi \rangle |&\lesssim {\mathscr {C}}_{0,1}(G_K) \, \Vert R \Vert \, \Vert \langle p \rangle \langle N^\frac{1}{2} \rangle \Psi \Vert ^2 \lesssim \ln K \, \Vert R \Vert \, \Vert \langle p \rangle \langle N^\frac{1}{2} \rangle \Psi \Vert ^2 \end{aligned}$$

and similarly for

3.37 $$\begin{aligned} |\langle \Psi , a({{\textbf {G}}}_K) R a^* ({{\textbf {G}}}_K) \Psi \rangle |&= |\langle (N+2)^\frac{1}{2} \Psi , a({{\textbf {G}}}_K) \langle N^\frac{1}{2} \rangle ^{-1} R \langle N^\frac{1}{2}\rangle ^{-1} a^* ({{\textbf {G}}}_K) (N+2)^\frac{1}{2} \Psi \rangle | \nonumber \\&\lesssim \Vert a({{\textbf {G}}}_K) \, \langle N^\frac{1}{2} \rangle ^{-1} R^\frac{1}{2} \Vert ^2 \, \Vert \langle N^\frac{1}{2} \rangle ^{-1} \Psi \Vert ^2 \nonumber \\&\lesssim \ln K \, \Vert \langle p \rangle R^{\frac{1}{2}} \Vert ^2 \Vert \langle N^\frac{1}{2} \rangle \Psi \Vert ^2. \end{aligned}$$

The remaining two diagonal terms in the estimate for $A_K R A_K$ are obtained in close analogy, as is the commutator bound (3.31b).

Finally, the two bounds for $D_K$ follow again directly from Lemma 3.4. □

In the next lemma, we recast the previous bounds in a form more suitable for the proof of our norm-approximation theorem in Sect. 7.

#### Lemma 3.6

Let $m \in \{0,1,2\}$. For all K⩾2, we have

3.38a $$\begin{aligned}&\Vert \langle N^\frac{1}{2}\rangle ^{m} \langle p\rangle ^{-1} (A_{K} - \phi ({{\textbf {G}}}_K)) \langle p\rangle ^{-1} \langle N^\frac{1}{2}\rangle ^{-m-1} \Vert \lesssim K^{-\frac{3}{4}} , \end{aligned}$$

3.38b $$\begin{aligned}&\Vert \langle N^\frac{1}{2}\rangle ^{m} \langle p\rangle ^{-1} A_{K} \langle p \rangle ^{-1} \langle N^\frac{1}{2}\rangle ^{-m-1}\Vert \lesssim ( \ln K)^\frac{1}{2} , \end{aligned}$$

3.38c $$\begin{aligned}&\Vert \langle N^\frac{1}{2}\rangle ^{m} \langle p\rangle ^{-2} [H_f, A_K ]\langle p\rangle ^{-2} \langle N^\frac{1}{2} \rangle ^{-m-1} \Vert \lesssim K , \end{aligned}$$

3.38d $$\begin{aligned}&\Vert \langle N^\frac{1}{2} \rangle ^{m} \langle p \rangle ^{-1} (D_{K} - 4\pi \ln K) \langle p \rangle ^{-1} \langle N^\frac{1}{2} \rangle ^{-m-2} \Vert \lesssim K^{-\frac{3}{4}}, \end{aligned}$$

3.38e $$\begin{aligned}&\Vert \langle N^\frac{1}{2} \rangle ^{m} \langle p \rangle ^{-1} D_{K}\langle p \rangle ^{-1} \langle N^\frac{1}{2} \rangle ^{-m-2} \Vert \lesssim \ln K , \end{aligned}$$

where $\Vert \cdot \Vert$ denotes the operator norm on $L^2({\mathbb {R}}^3_x) \otimes {\mathcal {F}}$.

#### Proof

For the first bound we note that $A_K - \phi ({{\textbf {G}}}_K) = 2 p\cdot a(k {{\textbf {B}}}_K) + 2 a^*(k {{\textbf {B}}}_K) \cdot p$. Commuting through the operator $\langle N^{\frac{1}{2}}\rangle ^{m}$ with *a* and $a^*$ (which shifts *N* by ±1) and applying (3.24c), we obtain

3.39 $$\begin{aligned} \Vert \langle N^\frac{1}{2}\rangle ^{m} \langle p\rangle ^{-1} (p \cdot a (k{{\textbf {B}}}_K) ) \langle p\rangle ^{-1} \langle N^\frac{1}{2}\rangle ^{-m-1} \Vert&\lesssim \Vert a (k {{\textbf {B}}}_K) \langle p\rangle ^{-1} \langle N^\frac{1}{2}\rangle ^{-1} \Vert \lesssim K^{-3/4}, \end{aligned}$$

3.40 $$\begin{aligned} \Vert \langle N^\frac{1}{2}\rangle ^{m} \langle p\rangle ^{-m-1} (a^*(k {{\textbf {B}}}_K) \cdot p) \langle p\rangle ^{-1} \langle N^\frac{1}{2}\rangle ^{-1} \Vert&\lesssim \Vert \langle N^\frac{1}{2}\rangle ^{-1} \langle p\rangle ^{-1} (a^*(k {{\textbf {B}}}_K) \cdot p) \langle p\rangle ^{-1} \Vert \nonumber \\&\lesssim \Vert a (k {{\textbf {B}}}_K) \langle p\rangle ^{-1} \langle N^\frac{1}{2}\rangle ^{-1} \Vert \nonumber \\&\lesssim K^{-3/4}. \end{aligned}$$

The second bound follows analogously, invoking also (3.24a).

For the commutator

3.41 $$\begin{aligned} [H_f, A_K] = a^*( \omega {{\textbf {G}}}_K ) - a( \omega {{\textbf {G}}}_K ) + 2 a^*( \omega k {{\textbf {B}}}_{K } )\cdot p - 2 p\cdot a ( \omega k {{\textbf {B}}}_{K} ), \end{aligned}$$

we apply (3.24b) and (3.24d):

3.42 $$\begin{aligned} \Vert \langle N^\frac{1}{2}\rangle ^{m} \langle p \rangle ^{-2} a (\omega {{\textbf {G}}}_K) \langle p\rangle ^{-2} \langle N^\frac{1}{2} \rangle ^{-m-1} \Vert&\lesssim K \end{aligned}$$

3.43 $$\begin{aligned} \Vert \langle N^\frac{1}{2}\rangle ^{m} \langle p\rangle ^{ -2} ( p \cdot a(\omega k {{\textbf {B}}}_K) ) \langle p \rangle ^{-2} \langle N^{\frac{1}{2}}\rangle ^{-m-2} \Vert&\lesssim \Vert a(\omega k {{\textbf {B}}}_K) \langle p \rangle ^{-2} \langle N^{\frac{1}{2}}\rangle ^{-1} \Vert \lesssim K^{-\frac{1}{2}} , \end{aligned}$$

and analogously for the terms with $a^*(\omega G_K)$ and $a^*(\omega k {{\textbf {B}}}_K) \cdot p$.

To obtain the fourth bound, we apply (3.24c) and (3.24f):

3.44 $$\begin{aligned} \Vert \langle N^\frac{1}{2} \rangle ^{m} \langle p \rangle ^{-1} a^*(k {{\textbf {B}}}_K) a(k{{\textbf {B}}}_K) \langle p \rangle ^{-1} \langle N^\frac{1}{2} \rangle ^{-m-2} \Vert&= \Vert \langle N^\frac{1}{2} \rangle ^{-1} \langle p \rangle ^{-1} a^*(k {{\textbf {B}}}_K) a(k{{\textbf {B}}}_K) \langle p \rangle ^{-1} \langle N ^\frac{1}{2} \rangle ^{-1} \Vert \nonumber \\&\lesssim \Vert a(k{{\textbf {B}}}_K) \langle p \rangle ^{-1} \langle N^\frac{1}{2} \rangle ^{-1} \Vert ^2\nonumber \\&\lesssim K^{-3/2} , \end{aligned}$$

3.45 $$\begin{aligned} \Vert \langle N^\frac{1}{2} \rangle ^{m} \langle p \rangle ^{-1} a( k {{\textbf {B}}}_{K} )^2 \langle p \rangle ^{-1} \langle N^\frac{1}{2} \rangle ^{-m-2} \Vert&\lesssim \Vert a( k {{\textbf {B}}}_{K} )^2 \langle p \rangle ^{-1} \langle N^\frac{1}{2} \rangle ^{-2} \Vert \lesssim K^{-\frac{3}{4}} , \end{aligned}$$

and similarly for $a^*(k {{\textbf {B}}}_K)^2$. The last bound is a direct consequence of the previous one. □

### Bounds for the classical potentials

We conclude with estimates for the classical potential $V_\varphi$ and the source term $\sigma (\psi )$, defined in (1.12) and (1.13), respectively. These bounds are used mainly for the aKG analysis in Sect. 5.

#### Lemma 3.7

There exists a constant C>0 such that the following bounds hold:

3.46a $$\begin{aligned} \Vert V_{\varphi } \psi \Vert _{L^2}&\leqslant C \Vert \varphi \Vert _{{\mathfrak {h}}_{1/2}} \, \Vert \psi \Vert _{H^1}&\qquad \quad&\forall \, \varphi \in {\mathfrak {h}}_{1/2},&\quad&\psi \in H^1, \end{aligned}$$

3.46b $$\begin{aligned} \Vert V_{\varphi } \psi \Vert _{L^2}&\leqslant C \Vert \varphi \Vert _{{\mathfrak {h}}_{-s}} \, \Vert \psi \Vert _{H^2}&\qquad \quad&\forall \, \varphi \in {\mathfrak {h}}_{-s},&\quad&\psi \in H^2, \quad s\in [0, \tfrac{3}{4} ], \end{aligned}$$

3.46c $$\begin{aligned} | \langle \psi , V_{\varphi } \psi \rangle |&\leqslant C \Vert \varphi \Vert _{{\mathfrak {h}}_{-1/2}} \Vert \psi \Vert _{H^1}^2&\qquad \quad&\forall \, \varphi \in {\mathfrak {h}}_{-1/2},&\quad&\psi \in H^1, \end{aligned}$$

3.46d $$\begin{aligned} | \langle \psi , V_{\varphi } \psi \rangle |&\leqslant C \Vert \varphi \Vert _{{\mathfrak {h}}_{1/2}} (\delta ^{-1} \Vert \psi \Vert _{L^2}^2 + \delta \Vert \psi \Vert _{H^1}^2 )&\qquad \quad&\forall \, \varphi \in {\mathfrak {h}}_{1/2},&\quad&\psi \in H^1, \quad \delta >0. \end{aligned}$$

In particular, the last inequality implies for all $\varphi \in {\mathfrak {h}}_{1/2}$ and $\psi \in H^1$:

3.47 $$\begin{aligned} \Vert \psi \Vert _{H^1}^2 \leqslant C (1+ \Vert \varphi \Vert _{{\mathfrak {h}}_{1/2}}^2 ) \Vert \psi \Vert _{L^2}^2 + C \langle \psi , (p^2 + V_\varphi ) \psi \rangle . \end{aligned}$$

#### Proof

The first two bounds can be viewed as commutative versions of Lemma 3.2. Set $f=\omega ^{-1/2}$ and replace the operator $a_k$ by the function φ(k). Proceeding as in Lemma 3.2, we find for $s\in {\mathbb {R}}$ and r⩾0:

3.48 $$\begin{aligned} \Big \Vert \int \text {d}k\, \omega (k)^{-1/2} e^{ikx} \varphi (k)\, \psi \Big \Vert ^2&= \int \text {d}k\,\text {d}\ell \, \omega ^{-\frac{1}{2}}(k)\,{\varphi (k)}\,\omega ^{-\frac{1}{2}}(\ell )\,{\varphi (\ell )}\, \Big \langle e^{i k x } \psi , e^{i\ell x} \psi \Big \rangle \nonumber \\&\leqslant \bigg \Vert \int \text {d}k \frac{1}{\omega ^{s+1}(k)\,\langle |p - k|^r \rangle ^2} \bigg \Vert \, \Vert \omega ^{s/2} \varphi \Vert _2^2 \, \Vert \langle |p|^r \rangle \psi \Vert ^2 \nonumber \\&\leqslant {\mathscr {C}}_{s,r} (\omega ^{-1/2})\, \Vert \varphi \Vert _{{\mathfrak {h}}_{s/2}}^2 \, \Vert \psi \Vert ^2_{H^r} \end{aligned}$$

with ${\mathscr {C}}_{s,r}(f)$ defined in Lemma 3.2. From (3.16), we infer that ${\mathscr {C}}_{1,1} (\omega ^{-1/2}) \lesssim 1$ and ${\mathscr {C}}_{-s,2} (\omega ^{-1/2}) \lesssim 1$ for $s\in [ 0, \frac{3}{4} ]$, which yields the first two statements.

For (3.46c), apply Cauchy–Schwarz:

3.49 $$\begin{aligned} | \langle \psi , V_{\varphi } \psi \rangle |&\leqslant 2 \int \text {d}k\, \omega ^{-1/2}(k)\, |\varphi (k)|\, | \widehat{|\psi |^2}(k) | \leqslant C \Vert \varphi \Vert _{{\mathfrak {h}}_{-1/2}} \Vert \psi \Vert _{L^4}^2 . \end{aligned}$$

By Sobolev’s inequality, $\Vert \psi \Vert _{L^4} \lesssim \Vert \psi \Vert _{H^1}$, this yields the claim.

To prove the last bound, decompose $\varphi =\varphi _K+\varphi _{>K}$ with $\varphi _K (k):= {\textbf{1}}_{|k|\leqslant K} \varphi (k)$, and estimate

3.50 $$\begin{aligned}&| \langle \psi , V_{\varphi _K} \psi \rangle | \leqslant \Vert \psi \Vert _{L^2}^2 \int _{|k|\leqslant K} \text {d}k\, \omega (k)^{-1/2} | \varphi (k) | \leqslant C K\, \Vert \varphi \Vert _{{\mathfrak {h}}_{1/2}} \Vert \psi \Vert _{L^2}^2 , \end{aligned}$$

3.51 $$\begin{aligned}&| \langle \psi , (V_{\varphi } - V_{\varphi _K}) \psi \rangle | \leqslant 2 K^{-1} \int _{|k|\geqslant K} \text {d}k\, \omega (k)^{1/2} |\varphi (k)|\, | \widehat{|\psi |^2}(k)| \leqslant 2K^{-1} \Vert \varphi \Vert _{{\mathfrak {h}}_{1/2}} \Vert \psi \Vert _{L^4}^2 , \end{aligned}$$

and hence

3.52 $$\begin{aligned} |\langle \psi , V_\varphi \psi \rangle | \leqslant C (K \Vert \psi \Vert _{L^2}^2 + K^{-1} \Vert \psi \Vert _{H^1}^2 ) \Vert \varphi \Vert _{{\mathfrak {h}}_{1/2}} \end{aligned}$$

for all K>0. Setting $\delta := K^{-1}$ proves the statement. □

#### Lemma 3.8

There is C>0 such that for all $L^2$-normalized $\psi \in H^1$ and $s\in [-\frac{1}{2},\frac{1}{2}]$,

3.53 $$\begin{aligned} \Vert \sigma (\psi )\Vert _{{\mathfrak {h}}_{s}}&\leqslant C \Vert \psi \Vert _{H^1}^2. \end{aligned}$$

Moreover, for all $L^2$-normalized $\psi ,\phi \in H^1$,

3.54 $$\begin{aligned} \Vert \sigma (\psi )-\sigma (\phi ) \Vert _{{\mathfrak {h}}_{-s}}&\leqslant C\,\big (\Vert \psi \Vert _{H^1}+\Vert \phi \Vert _{H^1}\big )\, \Vert \psi -\phi \Vert _{L^2}, \qquad s \in [ 0,1/2 ], \end{aligned}$$

3.55 $$\begin{aligned} \Vert \sigma (\psi ) - \sigma (\phi ) \Vert _{{\mathfrak {h}}_{1/2}}&\leqslant C \big ( \Vert \psi \Vert _{H^1} + \Vert \phi \Vert _{H^1} \big ) \Vert \psi - \phi \Vert _{H^1} . \end{aligned}$$

#### Proof

Recall that $\sigma (\psi )(k) = \omega (k)^{-1/2} \widehat{|\psi |^2}(k)=|k|^{-1/2} \widehat{|\psi |^2}(k)$. For the first bound, we split the integral into

3.56 $$\begin{aligned} \Vert \sigma (\psi ) \Vert _{{\mathfrak {h}}_{s}}^2 =\int \limits _{|k|\leqslant 1} \frac{|\widehat{|\psi |^2}(k)|^2}{|k|^{1+2s}} \text {d}k + \int \limits _{|k|>1} \frac{|\widehat{|\psi |^2}(k)|^2}{|k|^{1+2s}} \text {d}k =:I_1 + I_2. \end{aligned}$$

The low-momentum part satisfies $I_1 \leqslant \Vert \psi \Vert _{L^2}^4 \int _{|k|\leqslant 1 } |k|^{-1-2s} \text {d}k \lesssim 1$ for $s\in [-\frac{1}{2},\frac{1}{2}]$. For the large-momentum part, we use Plancherel and Sobolev to estimate

3.57 $$\begin{aligned} I_2 \;\leqslant \; \int _{{\mathbb {R}}^3} |\widehat{|\psi |^2}(k)|^2 \, \text {d}k = \Vert \psi \Vert _{L^4}^4 \lesssim \Vert \psi \Vert _{H^1}^4. \end{aligned}$$

For the second bound, set $\rho :=|\psi |^2-|\phi |^2$. By Plancherel, we have

3.58 $$\begin{aligned} \Vert \sigma (\psi )-\sigma (\phi )\Vert _{{\mathfrak {h}}_{-s}}^2 =\int \frac{|\widehat{\rho }(k)|^2}{|k|^{1+2s}} \, \text {d}k = c_s \iint \frac{\rho (x)\,\rho (y)}{|x-y|^{\,2-2s}}\, \text {d}x\, \text {d}y, \end{aligned}$$

for some constant $c_s$. Now apply the Hardy–Littlewood–Sobolev inequality [32, Theorem 4.3] for d=3, λ=2-2s, $s\in [0,\tfrac{1}{2}]$, and

3.59 $$\begin{aligned} p=q= p_s := \frac{3}{2+s} \in \big [\tfrac{6}{5},\tfrac{3}{2}\big ], \end{aligned}$$

to obtain $\Vert \sigma (\psi )-\sigma (\phi )\Vert _{{\mathfrak {h}}_{-s}}^2 \leqslant C_s\,\Vert \rho \Vert _{L^{p_s}}^2$. Write

3.60 $$\begin{aligned} \rho =|\psi |^2-|\phi |^2 = (\psi -\phi )\,\overline{\psi }+(\overline{\psi -\phi })\,\phi \end{aligned}$$

and take $q_s$ with $\frac{1}{p_s}=\frac{1}{2}+\frac{1}{q_s}$, i.e., $q_s= \frac{6}{1+2\,s}\in [2,6]$. Then by Hölder and Sobolev $H^1\hookrightarrow L^{q_s}$, we find

3.61 $$\begin{aligned} \Vert \rho \Vert _{L^{p_s}} \leqslant \Vert \psi -\phi \Vert _{L^2}\big (\Vert \psi \Vert _{L^{q_s}}+\Vert \phi \Vert _{L^{q_s}}\big ) \leqslant C\,\Vert \psi -\phi \Vert _{L^2}\big (\Vert \psi \Vert _{H^1}+\Vert \phi \Vert _{H^1}\big ) \end{aligned}$$

for all s∈[0,1/2] (Note that $C_s\lesssim 1$ uniformly on this interval).

For the last estimate, we have

3.62 $$\begin{aligned} \Vert \sigma (\psi ) - \sigma (\phi )\Vert _{{\mathfrak {h}}_{1/2}}&= \Vert |\psi |^2 - |\phi |^2\Vert _{L^2} . \end{aligned}$$

Using (3.60) and Hölder’s inequality, we find

3.63 $$\begin{aligned} \Vert |\psi |^2 - |\phi |^2\Vert _{L^2}&\leqslant \Vert \psi - \phi \Vert _{L^6} \, \big ( \Vert \psi \Vert _{L^3} + \Vert \phi \Vert _{L^3} \big ). \end{aligned}$$

By Sobolev embedding $H^1({\mathbb {R}}^3)\hookrightarrow L^6({\mathbb {R}}^3)$,

3.64 $$\begin{aligned} \Vert \psi -\phi \Vert _{L^6} \leqslant C\,\Vert \nabla (\psi -\phi )\Vert _{L^2} \leqslant C\,\Vert \psi -\phi \Vert _{H^1}. \end{aligned}$$

To bound the $L^3$-norms, we use $\Vert \psi \Vert _{L^3} \leqslant C\, \Vert \psi \Vert _{H^1}^{1/2}$. Thus

3.65 $$\begin{aligned} \Vert \sigma (\psi )-\sigma (\phi )\Vert _{{\mathfrak {h}}_{1/2}} = \Vert |\psi |^2-|\phi |^2\Vert _{L^2}&\leqslant C\,\Vert \psi -\phi \Vert _{H^1}\,\big (\Vert \psi \Vert _{H^1}+\Vert \phi \Vert _{H^1}\big ). \end{aligned}$$

as claimed. □

## The dressed Nelson Hamiltonian

In this section, we introduce the dressed Nelson Hamiltonian, which is related to the renormalized Nelson Hamiltonian $H^\varepsilon$ through the unitary dressing transformation (2.6). The dressing transformation allows us to isolate the UV singular term and work with the dressed Hamiltonian. While the dressed Hamiltonian has a more complicated (quadratic) structure, it involves more regular kernels. In a second step, we derive a suitable Weyl-shifted representation of the dressed Hamiltonian.

### Lemma 4.1

Let $U_K^\varepsilon$ be defined as in (2.6). There exists $K_0>0$ such that for all $K \geqslant K_0$ and ε>0, the renormalized Hamiltonian satisfies

4.1 $$\begin{aligned} H^{\varepsilon } = (U_K^\varepsilon )^* H_{{\mathscr {D}},K}^{\varepsilon } U_K^\varepsilon , \end{aligned}$$

where the dressed Hamiltonian is given by

4.2 $$\begin{aligned}&H_{{\mathscr {D}},K}^{\varepsilon } = p^2 + \varepsilon \Big ( \phi ( {{\textbf {G}}}_K ) + 2 a^*( k {{\textbf {B}}}_K) \cdot p + 2 p \cdot a ( k {{\textbf {B}}}_K ) \Big ) \nonumber \\&\quad + \varepsilon ^2 \Big ( H_f + 2 a^*( k {{\textbf {B}}}_K ) a( k {{\textbf {B}}}_K ) + a^*( k {{\textbf {B}}}_K )a^*( k {{\textbf {B}}}_K ) + a( k {{\textbf {B}}}_K ) a( k {{\textbf {B}}}_K ) + 4\pi \ln K \Big ) \nonumber \\&\quad - \varepsilon ^3 \phi (\omega {{\textbf {B}}}_K) + \varepsilon ^4 \Vert \omega ^{\frac{1}{2}} B_K \Vert ^2_{L^2} \end{aligned}$$

with quadratic form domain $Q(H_{{\mathscr {D}},K}^{\varepsilon }) = Q(p^2 + H_f )$.

Moreover, there are constants $C,K_0>0$ such that

4.3 $$\begin{aligned} \pm \big ( H_{{\mathscr {D}},K}^{\varepsilon } - \big ( p^2 + \varepsilon ^2 H_f \big ) \big )&\leqslant \tfrac{1}{2} \big ( p^2 + \varepsilon ^2 H_f \big ) + C K , \end{aligned}$$

4.4 $$\begin{aligned} \pm \big ( H_{{\mathscr {D}},K}^{\varepsilon } - \big ( p^2 + \varepsilon ^2 H_f \big ) \big )&\leqslant \tfrac{1}{2} \big ( p^2 + \varepsilon ^2 H_f \big ) + C\varepsilon ^2 ( \ln K ) (N+1) \end{aligned}$$

in the sense of quadratic forms, for all $K\geqslant K_0$ and ε>0.

The dressing identity for $H^\varepsilon$ follows the same strategy as in [16, Lem. 3.1]. Because of the semiclassical scaling and our slightly modified choice of dressing transformation, we give the details in Appendix A.

The two energy bounds follow from Lemmas 3.1 and 3.4. For (4.3), the contribution proportional to *K* comes from the field operator, estimated as

4.5 $$\begin{aligned} \big |\langle \Psi , \varepsilon \, a({\boldsymbol{G}}_K)\,\Psi \rangle \big | \;\leqslant \; C\,K^{1/2}\,\Vert \Psi \Vert \,\Vert \varepsilon \,H_f^{1/2}\Psi \Vert . \end{aligned}$$

In (4.4), the dependence on *K* is improved at the expense of involving the number operator. Here one uses in particular

4.6 $$\begin{aligned} \big |\langle \Psi , \varepsilon \, a({\boldsymbol{G}}_K)\Psi \rangle \big | \;=\;\big |\langle \langle N^{1/2}\rangle \Psi , \varepsilon \,a({\boldsymbol{G}}_K)N^{-1/2}\Psi \rangle \big | \;\leqslant \; C\,\varepsilon \,(\ln K)^{1/2}\,\Vert \langle N^{1/2}\rangle \Psi \Vert \,\Vert \langle p\rangle \Psi \Vert . \end{aligned}$$

The estimates for the remaining terms are straightforward and omitted here.

Next, we compute a Weyl-shifted representation of the dressed Hamiltonian, which will be useful for the proof in Sect. 7. This transformation isolates the leading-order particle Hamiltonian and expresses the remainder in increasing powers of ε. For $\varphi \in {\mathfrak {h}}_{1/2}$, consider

4.7 $$\begin{aligned} {\mathscr {H}}^{\varepsilon }_{{\mathscr {D}},K,\varphi }&:= e^{-i g_{K,\varphi }(x)} W(\varepsilon ^{-1} \varphi )^* H^{ \varepsilon }_{{\mathscr {D}} , K} W(\varepsilon ^{-1} \varphi ) e^{ig_{K,\varphi }(x)} \end{aligned}$$

where $g_{K,\varphi }(x) = 2 \text {Im}\langle \varphi , {{\textbf {B}}}(\cdot , k) \rangle$, so that $e^{-ig_{K,\varphi }(x)}$ corresponds to the classical analogue of the dressing transformation $U_K^\varepsilon$. A straightforward computation yields

4.8 $$\begin{aligned} W(\varepsilon ^{-1} \varphi )^* H^{ \varepsilon }_{{\mathscr {D}} ,K} W(\varepsilon ^{-1} \varphi )&= {\mathfrak {h}}_0 + \varepsilon {\mathfrak {h}}_1 + \varepsilon ^2 {\mathfrak {h}}_2 - \varepsilon ^3 \phi (\omega {{\textbf {B}}}_K ) + \varepsilon ^4 \Vert \omega ^{\frac{1}{2}} B_K \Vert ^2_{L^2} \end{aligned}$$

with

4.9 $$\begin{aligned} {\mathfrak {h}}_0&= p^2 + 2 \text {Re}\langle \varphi , {{\textbf {G}}}_K \rangle + 2 \langle \varphi , k {{\textbf {B}}}_K \rangle \cdot p + 2 p \cdot \langle k {{\textbf {B}}}_K , \varphi \rangle + 4 |\text {Re}\langle \varphi , k {{\textbf {B}}}_K \rangle |^2 + \Vert \omega ^{\frac{1}{2}} \varphi \Vert _{L^2}^2 , \end{aligned}$$

4.10 $$\begin{aligned} {\mathfrak {h}}_1&= \phi ({{\textbf {G}}}_K + \omega \varphi ) + 2 a^*(k {{\textbf {B}}}_K)\cdot p +2 p \cdot a(k {{\textbf {B}}}_K) + 4\text {Re}\langle \varphi , k {{\textbf {B}}}_K \rangle \cdot \phi (k {{\textbf {B}}}_K), \end{aligned}$$

4.11 $$\begin{aligned} {\mathfrak {h}}_2&= H_f + 2 a^*(k {{\textbf {B}}}_K) a (k {{\textbf {B}}}_K) + a^*( k {{\textbf {B}}}_K )^2+ a( k {{\textbf {B}}}_K )^2 + 4\pi \ln K - 2 \text {Re}\langle {{\textbf {B}}}_K , \omega \varphi \rangle . \end{aligned}$$

Transforming (4.8) with $e^{-i g_{K,\varphi }(x)}$ then yields

4.12 $$\begin{aligned} {\mathscr {H}}_{{\mathscr {D}},K,\varphi }^{\varepsilon }&= h_\varphi + \Vert \omega ^{\frac{1}{2}} \varphi \Vert ^2_{L^2} + \varepsilon \Big ( A_K + \phi (\omega \varphi ) \Big ) + \varepsilon ^2 \Big ( H_f + D_K - 2 \text {Re}\langle \omega \varphi , {{\textbf {B}}}_K \rangle \Big ) \nonumber \\&\quad - \varepsilon ^3 \phi (\omega {{\textbf {B}}}_K) + \varepsilon ^4 \Vert \omega ^{\frac{1}{2}} B_K \Vert ^2_{L^2} \end{aligned}$$

where $h_\varphi = p^2 + 2 \text {Re}\langle \varphi , {{\textbf {G}}} \rangle$ as in (SKG), and we recall the definitions of $A_K$ and $B_K$ in (3.28) and (3.29), respectively

Let us briefly explain where the different terms come from. Using

4.13 $$\begin{aligned} e^{-ig_{K,\varphi }(x)}\, p \,e^{ig_{K,\varphi }(x)} = p - 2\,\text {Re}\langle \varphi , k{{\textbf {B}}}_K\rangle , \end{aligned}$$

one obtains

4.14 $$\begin{aligned} e^{-ig_{K,\varphi }(x)} {\mathfrak {h}}_0 e^{ig_{K,\varphi }(x)}&= \big ( p - 2 \text {Re}\langle \varphi , k {{\textbf {B}}}_K \rangle \big )^2 + 2 \text {Re}\langle \varphi , {{\textbf {G}}}_K \rangle + 2\langle \varphi , k {{\textbf {B}}}_K \rangle \cdot \big ( p - 2 \text {Re}\langle \varphi , k {{\textbf {B}}}_K \rangle \big ) \nonumber \\&\quad + \big ( p- 2 \text {Re}\langle \varphi , k {{\textbf {B}}}_K \rangle \big ) \cdot \langle k {{\textbf {B}}}_K , \varphi \rangle + 4 |\text {Re}\langle \varphi , k {{\textbf {B}}}_K \rangle |^2 + \Vert \omega ^{\frac{1}{2} } \varphi \Vert ^2_{L^2} \nonumber \\&= p^2 + 2 \text {Re}\langle \varphi , {{\textbf {G}}}_K \rangle + 2\text {Im}\langle \varphi , k {{\textbf {B}}}_K \rangle \cdot p + 2 p \cdot \text {Im}\langle k {{\textbf {B}}}_K , \varphi \rangle + \Vert \omega ^{\frac{1}{2}} \varphi \Vert _{L^2}^2\nonumber \\&= p^2 + 2 \text {Re}\langle \varphi , {{\textbf {G}}}_K \rangle + 2 \text {Re}\langle \varphi , k^2 {{\textbf {B}}}_K \rangle + \Vert \omega ^{\frac{1}{2}} \varphi \Vert _{L^2}^2 \end{aligned}$$

where we used $p \cdot \text {Im}\langle \varphi , k {{\textbf {B}}}_K \rangle = - \text {Im}\langle k {{\textbf {B}}}_K, \varphi \rangle \cdot p + \text {Re}\langle \varphi , k^2 {{\textbf {B}}}_K \rangle$ in the last step. Invoking the identity ${{\textbf {G}}}_K + k^2 {{\textbf {B}}}_K = {{\textbf {G}}}$ explains the presence of the operator $h_\varphi$. We further note that

4.15 $$\begin{aligned}&e^{-ig_{K,\varphi }(x)} {\mathfrak {h}}_1 e^{ig_{K,\varphi }(x) } \nonumber \\&\quad = \phi ( {{\textbf {G}}}_K + \omega \varphi ) + 2 a^*(k {{\textbf {B}}}_K) \cdot \big ( p - 2 \text {Re}\langle \varphi , k {{\textbf {B}}}_K \rangle \big ) + 2 \big ( p - 2 \text {Re}\langle \varphi , k {{\textbf {B}}}_K \rangle \big ) \cdot a(k {{\textbf {B}}}_K) \nonumber \\&\quad \quad + 4\text {Re}\langle \varphi , k {{\textbf {B}}}_K \rangle \cdot \phi (k {{\textbf {B}}}_K) \nonumber \\&\quad = \phi ( {{\textbf {G}}}_K + \omega \varphi ) + 2 a^*(k {{\textbf {B}}}_K) \cdot p + 2 p \cdot a(k {{\textbf {B}}}_K) \end{aligned}$$

which explains the terms of order O(ε). The higher-order terms in ε in (4.8) do not involve gradients, so that they commute with $e^{ig_{K,\varphi } (x)}$.

## Well-posedness and properties of the aKG equation

In the following we prove Proposition 1.2, that is, we establish local well-posedness of the aKG equation. Moreover, we derive an equation of motion for the ground state $\psi _{\varphi _t}$ and establish some important estimates for $\psi _{\varphi _t}$ and the resolvent $R_{\varphi _t}$.

We recall the notation for the lowest eigenvalue $e(\varphi ) = \inf \sigma (h_\varphi )$ of the operator $h_\varphi$ as well as for the spectral gap $\triangle (\varphi ) = \inf \{ |e(\varphi )-\lambda |: \lambda \in \sigma (h_{\varphi })\setminus \{e(\varphi )\} \}$. Let us further recall that $\varphi \in C([-T,T];{\mathfrak {h}}_{1/2})$ is called a solution to (aKG) with initial condition $\varphi _0\in {\mathfrak {h}}_{1/2}$, in case that $e(\varphi _t)<0$ for all t∈[-T,T] and

5.1 $$\begin{aligned} \varphi _t =e^{-i\omega t}\varphi _0-i\int _0^t e^{-i\omega (t-s)} \sigma ( \psi _{\varphi _s}) \textrm{d}s, \end{aligned}$$

where $\psi _{\varphi _t}\geqslant 0$ is the unique ground state of $h_{\varphi _t}$ and $\sigma (\psi _{\varphi _s}) = \omega ^{-1/2} \widehat{| \psi _{\varphi _s} |^2}$. Existence and uniqueness of $\psi _{\varphi _t}$ follow from the condition $e(\varphi _t) < 0$.

We prepare the proof of Proposition 1.2 with two lemmas: the first establishes local stability of the spectral gap, and the second shows that the map $\varphi \mapsto \psi _\varphi$ is locally Lipschitz.

### Lemma 5.1

Let $\varphi \in {\mathfrak {h}}_{1/2}$ with e(φ)<0 and spectral gap $\triangle (\varphi )>0$ and let

5.2 $$\begin{aligned} B_r(\varphi ) := \big \{ u \in {\mathfrak {h}}_{1/2} : \Vert u - \varphi \Vert _{{\mathfrak {h}}_{1/2}} \leqslant r \big \}. \end{aligned}$$

Then there exist C>0 and $r_0>0$ such that for all $0<r\leqslant r_0$ and all $u\in B_r(\varphi )$,

5.3 $$\begin{aligned} \triangle (u)\ \geqslant \ \tfrac{1}{2}\,\triangle (\varphi )\ >0 . \end{aligned}$$

Moreover, $|e(\varphi ) - e(u)| + |\lambda _1(\varphi ) - \lambda _1(u) | \leqslant C r$ for all $u \in B_r(\varphi )$, where $\lambda _1(u)$ denotes the first excited eigenvalue of $h_u$.

### Proof

By Lemma 3.7, there is C>0 so that for all $\varphi ,\xi \in {\mathfrak {h}}_{1/2}$ and all $\psi \in H^1$ with $\Vert \psi \Vert _{L^2}=1$,

5.4 $$\begin{aligned} \big |\langle \psi ,(h_\varphi -h_\xi )\psi \rangle \big | \ \leqslant \ C\,\Vert \varphi -\xi \Vert _{{\mathfrak {h}}_{1/2}}\ \Vert \psi \Vert _{H^1}^2 . \end{aligned}$$

Let $e(u)=\inf \sigma (h_u)$ and let $\psi _{\varphi }$ denote the ground state of $h_{\varphi }$. The variational principle combined with the above inequality gives

5.5 $$\begin{aligned} e(u)\ \leqslant \ \langle \psi _{\varphi },h_u\psi _{\varphi }\rangle \ \leqslant \ e(\varphi )+C\,\Vert \varphi - u \Vert _{{\mathfrak {h}}_{1/2}} \Vert \psi _{\varphi }\Vert _{H^1}^2 \ \leqslant \ e(\varphi ) + C r , \end{aligned}$$

and thus for *r* sufficiently small, e(u)<0. Denoting now by $\psi _u$ the ground state of $h_u$ and exchanging φ,u in the above estimate yields

5.6 $$\begin{aligned} |e(\varphi )-e(u)| \ \leqslant \ C r \end{aligned}$$

for some constant *C* depending only on φ. Note that here we also used the estimate

5.7 $$\begin{aligned} \Vert \psi _u \Vert _{H^1}^2 \leqslant C \big ( 1+ \Vert u \Vert _{{\mathfrak {h}}_{1/2}}^2 + e(u) \big ) \leqslant C \big ( 1 + \Vert \varphi \Vert _{{\mathfrak {h}}_{1/2}}^2 \big ) \leqslant C \end{aligned}$$

as a consequence of Lemma 3.7.

Next, write $\lambda _1(u):=\inf \big (\sigma (h_u)\setminus \{e(u)\}\big )$, and recall by the max-min principle that

5.8 $$\begin{aligned} \lambda _1(u)=\sup _{\textrm{codim}(V)=1}\ \inf _{\psi \in V} \big \{ \langle \psi ,h_u \psi \rangle : \Vert \psi \Vert _{L^2}=1\big \}. \end{aligned}$$

Choosing $V=\{ \psi _\varphi \}^\perp$ and using (5.4), we obtain

5.9 $$\begin{aligned} \lambda _1(u)&\geqslant \inf _{\begin{array}{c} \psi \in H^1,\ \Vert \psi \Vert =1\\ \psi \perp \psi _{\varphi } \end{array}} \langle \psi ,h_u\psi \rangle \ \geqslant \ \inf _{\begin{array}{c} \psi \in H^1,\ \Vert \psi \Vert =1\\ \psi \perp \psi _{\varphi } \end{array}} \left( \langle \psi ,h_{\varphi }\psi \rangle \;-\;C r \Vert \psi \Vert _{H^1}^2 \right) . \end{aligned}$$

Using the bound (3.47) and $\Vert \varphi \Vert _{{\mathfrak {h}}_{1/2}} \leqslant C$, we have $\Vert \psi \Vert _{H^1}^2 \leqslant C ( 1 + \langle \psi , h_\varphi \psi \rangle )$, and thus

5.10 $$\begin{aligned} \lambda _1(u)&\geqslant (1-Cr) \inf _{\begin{array}{c} \psi \in H^1,\ \Vert \psi \Vert =1 \\ \psi \perp \psi _{\varphi } \end{array}} \langle \psi ,h_{\varphi }\psi \rangle - C r = (1-Cr) \lambda _1(\varphi ) - C r . \end{aligned}$$

Since $\triangle (u)=\lambda _1(u)-e(u)$, combining the above estimate with (5.6), we arrive at

5.11 $$\begin{aligned} \triangle (u)\ \geqslant \ \triangle (\varphi )-C r . \end{aligned}$$

Choosing $r_0>0$ sufficiently small then yields $\triangle (u)\geqslant \tfrac{1}{2}\,\triangle (\varphi )>0$ for all $u\in B_r(\varphi )$ with $0<r\leqslant r_0$. Exchanging the roles of *u* and φ in the argument for $\lambda _1$ gives the reverse comparison

5.12 $$\begin{aligned} \lambda _1(\varphi )&\geqslant (1-Cr)\,\lambda _1(u) - C r \end{aligned}$$

with a constant *C* that depends only on φ, hence $|\lambda _1(u)-\lambda _1(\varphi )| \leqslant C r$. Together with (5.6) this proves the last statement. □

### Lemma 5.2

Let $\varphi \in {\mathfrak {h}}_{1/2}$ with e(φ)<0, and define $B_r(\varphi )$ as in Lemma 5.1. There are constants C>0 and $r_0>0$ such that

5.13 $$\begin{aligned} \Vert \psi _u - \psi _\xi \Vert _{H^1}&\leqslant \frac{C}{\triangle (\varphi )^{1/2}} \left( \Vert \psi _u \Vert _{H^1} + \Vert \psi _\xi \Vert _{H^1} \right) \Vert u - \xi \Vert _{{\mathfrak {h}}_{1/2}} \end{aligned}$$

for all $u, \xi \in B_r(\varphi )$ with $0<r\leqslant r_0$, where $\psi _u$ and $\psi _\xi$ denote the normalized non-negative ground states of $h_\varphi$ and $h_\xi$, respectively. Moreover for s∈[0,3/4]

5.14 $$\begin{aligned} \Vert \psi _u - \psi _\xi \Vert _{L^2}&\leqslant \frac{C}{\triangle (\varphi )^{1/2}} \left( \Vert \psi _u \Vert _{H^2} + \Vert \psi _\xi \Vert _{H^2} \right) \Vert u - \xi \Vert _{{\mathfrak {h}}_{-s}} \end{aligned}$$

for all $u, \xi \in B_r(\varphi ) \cap {\mathfrak {h}}_{-1/2}$ with $0< r \leqslant r_0$.

### Proof

We apply (3.47) to $\psi _{u} - \psi _{\xi } \in H^1$ and $u-\xi \in {\mathfrak {h}}_{1/2}$, thereby obtaining

5.15 $$\begin{aligned} \Vert \psi _{u} - \psi _{\xi } \Vert _{H^1}^2 \leqslant C \left( (1+ \Vert u - \xi \Vert ^2_{{\mathfrak {h}}_{1/2}} ) \Vert \psi _{u} - \psi _{\xi } \Vert _{L^2}^2 + \langle \psi _{u} - \psi _{\xi } , h_{u - \xi } (\psi _{u} - \psi _{\xi } )\rangle \right) . \end{aligned}$$

For the $L^2$-norm, we use the bound $\Vert \psi _{u} - \psi _{\xi }\Vert _{L^2}^2 \leqslant 2 \Vert Q_{u} \psi _{\xi } \Vert _{L^2}^2$, where $Q_{u} = {\boldsymbol{1}} - | \psi _{u} \rangle \langle \psi _{u} |$, as well as

5.16 $$\begin{aligned} \Vert Q_{u} \psi _{\xi } \Vert _{L^2}^2 \leqslant \frac{1}{\triangle (u)}\langle \psi _{\xi },(h_{u}-e(u)) \psi _{\xi } \rangle \leqslant \frac{2}{\triangle (\varphi )}\langle \psi _{\xi },(h_{u}-e(u)) \psi _{\xi } \rangle \end{aligned}$$

where we applied Lemma 5.1 in the last step. We continue to estimate

5.17 $$\begin{aligned} \langle \psi _{\xi },(h_{u}-e(u)) \psi _{\xi } \rangle&= \langle \psi _{\xi } - \psi _u , h_{u} \psi _{\xi } \rangle - \langle \psi _{\xi } - \psi _u , \psi _{\xi } \rangle \langle \psi _{u} , h_u \psi _u \rangle \nonumber \\&= e(\xi ) \langle \psi _{\xi } - \psi _u , \psi _{\xi } \rangle + \langle \psi _{\xi } - \psi _u , V_{u - \xi } \psi _{\xi } \rangle \nonumber \\&\quad - \langle \psi _{\xi } - \psi _u , \psi _{\xi } \rangle \left( \langle \psi _{u} , h_\xi \psi _u \rangle + \langle \psi _{u} , V_{u - \xi } \psi _u \rangle \right) , \end{aligned}$$

where we used $h_u = h_\xi + V_{u - \xi }$. Since $e(\xi ) \leqslant \langle \psi _{u}, h_\xi \psi _u \rangle$ and $\langle \psi _\xi - \psi _u, \psi _\xi \rangle \geqslant 0$, it follows that

5.18 $$\begin{aligned} \langle \psi _{\xi },(h_{u}-e(u)) \psi _{\xi } \rangle&\leqslant \Vert \psi _\xi - \psi _u \Vert _{L^2} \left( \Vert V_{u -\xi } \psi _\xi \Vert _{L^2} + \Vert V_{u -\xi } \psi _u \Vert _{L^2} \right) \nonumber \\&\leqslant C \Vert \psi _\xi - \psi _u \Vert _{L^2} \left( \Vert \psi _\xi \Vert _{H^1} + \Vert \psi _u \Vert _{H^1} \right) \Vert u - \xi \Vert _{{\mathfrak {h}}_{1/2}} \nonumber \\&\leqslant \tfrac{1}{10} \Vert \psi _\xi - \psi _u \Vert _{L^2}^2 + C \left( \Vert \psi _u \Vert _{H^1} + \Vert \psi _\xi \Vert _{H^1} \right) ^2 \Vert u - \xi \Vert _{{\mathfrak {h}}_{1/2}}^2, \end{aligned}$$

where the second step follows from (3.46a). Hence, we get a bound of the form

5.19 $$\begin{aligned} \Vert \psi _u - \psi _\xi \Vert _{L^2}^2 \leqslant C \triangle (\varphi )^{-1} \left( \Vert \psi _u \Vert _{H^1} + \Vert \psi _\xi \Vert _{H^1} \right) ^2 \Vert u - \xi \Vert _{{\mathfrak {h}}_{1/2}}^2. \end{aligned}$$

It remains to estimate the middle term in (5.15). Using $h_{u - \xi } = h_u - V_\xi$:

5.20 $$\begin{aligned} \langle \psi _{u} - \psi _{\xi } , h_{u - \xi } (\psi _{u} - \psi _{\xi } )\rangle&= \langle \psi _{u} - \psi _{\xi } , h_u \psi _{u} - \psi _{\xi } \rangle - \langle \psi _{u} - \psi _{\xi } , V_\xi (\psi _{u} - \psi _{\xi } )\rangle \nonumber \\&\leqslant \langle \psi _{\xi } , ( h_u - e(u)) \psi _{\xi } \rangle - \langle \psi _{u} - \psi _{\xi } , V_\xi (\psi _{u} - \psi _{\xi } )\rangle , \end{aligned}$$

where we also used e(u)<0 and $(h_u - e(u) ) \psi _u = 0$. The first term was estimated in (5.18). For the second term we find by Cauchy–Schwarz, (3.46a) and $\Vert \xi \Vert _{{\mathfrak {h}}_{1/2}}\leqslant C$ that

5.21 $$\begin{aligned} | \langle \psi _{u} - \psi _{\xi } , V_\xi (\psi _{u} - \psi _{\xi } )\rangle |&\leqslant C \Vert \psi _{u} - \psi _{\xi } \Vert _{L^2} \Vert \psi _{u } - \psi _{\xi } \Vert _{H^1}. \end{aligned}$$

Adding everything together proves the first statement of the lemma.

For the second one, we proceed as in (5.16)–(5.18) to find

5.22 $$\begin{aligned} \Vert \psi _u - \psi _\xi \Vert _{L^2} \leqslant C \triangle (\varphi )^{-1} \left( \Vert V_{u -\xi } \psi _\xi \Vert _{L^2} + \Vert V_{u -\xi } \psi _u \Vert _{L^2} \right) . \end{aligned}$$

From here, the statement follows from (3.46b). □

We can now prove well-posedness of the aKG equation.

### Proof of Proposition 1.2

By assumption $e(\varphi _0)<0$, the spectral gap $\triangle (\varphi _0)>0$ is strictly positive. Therefore by Lemma 5.1, we have ▵(u)>0 for all $u \in B_r(\varphi _0)$ for sufficiently small r>0. Thus, for every $u \in B_r(\varphi _0)$ there is a unique normalized ground state $\psi _u \geqslant 0$ of $h_u$ with eigenvalue e(u)<0, and by Lemma 3.7 (3.47)

5.23 $$\begin{aligned} \Vert \psi _{u}\Vert _{H^1}\le C\left( 1+\Vert u \Vert _{{\mathfrak {h}}_{1/2}} \right) \leqslant \lambda \end{aligned}$$

for some λ>0 depending only on *r* and $\Vert \varphi _0 \Vert _{{\mathfrak {h}}_{1/2}}$.

*Fixed-point map.* On the metric space $X_T:=C([-T,T];B_r(\varphi _0))$, we define

5.24 $$\begin{aligned} ({\mathcal {T}}u)(t):=e^{-it \omega }\varphi _0-i\int _0^t e^{i(s-t)\omega }\, \sigma (\psi _{u_s}) \, \textrm{d}s, \qquad |t|\leqslant T, \end{aligned}$$

which we use to express (1.18) as a fixed-point equation ${\mathcal {T}}(\varphi )=\varphi$.

*Contraction.* To show that ${\mathcal {T}}$ maps $X_T$ into itself, we first estimate

$$\begin{aligned} \left\| {\mathcal {T}}u(t)-\varphi _0\right\| _{{\mathfrak {h}}_{1/2}} \le \left\| (e^{-it\omega }-1)\varphi _0\right\| _{{\mathfrak {h}}_{1/2}} + T \sup _{|s|\leqslant T} \Vert \sigma (\psi _{u_s}) \Vert _{{\mathfrak {h}}_{1/2}}. \end{aligned}$$

By dominated convergence $\lim _{t\rightarrow 0} \Vert (e^{-it\omega }-1)\varphi _0\Vert _{{\mathfrak {h}}_{1/2}}=0$, and using Lemma 3.8 together with (5.23), we obtain

5.25 $$\begin{aligned} \Vert \sigma (\psi _{u_s}) \Vert _{{\mathfrak {h}}_{1/2}} \leqslant C \Vert \psi _{u_s}\Vert _{H^1}^2 \leqslant C\,\lambda ^2. \end{aligned}$$

Analogously, $\left\| ({\mathcal {T}}u)(t)-({\mathcal {T}}u)(t_0)\right\| _{{\mathfrak {h}}_{1/2}}\underset{t\rightarrow t_0}{\longrightarrow }\ 0$ for any $u\in X_T$, where $t,t_0\in [-T,T]$. Choosing *T* small enough therefore yields ${\mathcal {T}}(X_T)\subseteq X_T$.

For $u,\xi \in X_T$, by Lemma 3.8 and the Lipschitz continuity of $u\mapsto \psi _u$ (Lemma 5.2 and (5.23)), we further have

5.26 $$\begin{aligned} \Vert {\mathcal {T}} u(t)-{\mathcal {T}} \xi (t)\Vert _{{\mathfrak {h}}_{1/2}} \leqslant \int _0^{|t|} \Vert \sigma (\psi _{u_s})-\sigma (\psi _{\xi _s})\Vert _{{\mathfrak {h}}_{1/2}}\,\mathrm ds&\leqslant C| t| \lambda \,\sup _{|s|\leqslant T}\Vert \psi _{u_s}-\psi _{\xi _s}\Vert _{H^1} \nonumber \\&\leqslant C D |t| \lambda ^2 \,\sup _{|s|\leqslant T}\Vert u_s-\xi _s\Vert _{{\mathfrak {h}}_{1/2}}, \end{aligned}$$

hence

5.27 $$\begin{aligned} \Vert {\mathcal {T}} u-{\mathcal {T}} \xi \Vert _{X_T}\;\leqslant \; T\,C D \lambda ^2\,\Vert u-\xi \Vert _{X_T}. \end{aligned}$$

Choose *T* even smaller, if necessary, so that $T C D \lambda ^2 <1$. Then ${\mathcal {T}}$ is a strict contraction on $X_T$, and there exists a unique fixed point $\varphi \in X_T$ with ${\mathcal {T}}(\varphi )=\varphi$. By construction, φ satisfies (1.18) and $e(\varphi _t)<0$ for |t|⩽T, hence $h_{\varphi _t}$ has a unique ground state for those times. In particular, $\triangle (\varphi _t)\geqslant \triangle (\varphi _0)/2$ by Lemma 5.1.

*Uniqueness.* Let $\xi \in C([-T,T];{\mathfrak {h}}_{1/2})$ be any solution of (1.18) with the same initial datum $\xi _0=\varphi _0$. By Lemma 3.7 and $e(\xi _{t})<0$, we have $\Vert \psi _{\xi _s}\Vert _{H^1}^2\le C ( 1 + \Vert \xi _s\Vert ^2_{{\mathfrak {h}}_{1/2}})$, and therefore

5.28 $$\begin{aligned} \Vert \xi _t- \varphi _0\Vert _{{\mathfrak {h}}_{1/2}} \le \left\| (e^{-it\omega }-1)\varphi _0\right\| _{{\mathfrak {h}}_{1/2}} + C T \sup _{|s| \leqslant T } C ( 1 + \Vert \xi _s\Vert ^2_{{\mathfrak {h}}_{1/2}}) \end{aligned}$$

By continuity of $t \mapsto \Vert \xi _t \Vert _{{\mathfrak {h}}_{1/2}}$, we conclude that $\xi \in C([-T,T];B_r(\varphi _0))$ for *T* small enough. By the contraction argument above the fixed point is unique in $X_T$, so $\xi =\varphi$ on [-T,T].

$C^1$-*regularity.* Set $g(t):=\sigma (\psi _{\varphi _t})$. The map t↦g(t) is continuous in ${\mathfrak {h}}_{-1/2}$ by Lemma 3.8, (5.23), and Lemma 5.2. Let $v(t):=e^{i\omega t}\varphi _t$. From (1.18),

5.29 $$\begin{aligned} v(t)=\varphi _0 - i\int _0^t e^{i\omega s} \, g(s)\,\mathrm ds, \end{aligned}$$

and since $g\in C([-T,T];{\mathfrak {h}}_{-1/2})$, the integral is $C^1$ with $\partial _t v(t)=-i\,e^{i\omega t}\,g(t)$ in ${\mathfrak {h}}_{-1/2}$. Thus $\varphi _t=e^{-i\omega t}v(t)\in C^1([-T,T];{\mathfrak {h}}_{-1/2})$ and satisfies the differential aKG in ${\mathfrak {h}}_{-1/2}$.

$L^2$-*persistence and continuity.* Assume $\varphi _0\in L^2$. From (1.18) and the unitarity of $e^{-i\omega t}$,

5.30 $$\begin{aligned} \Vert \varphi _t\Vert _{L^2} \;\leqslant \; \Vert \varphi _0\Vert _{L^2} \;+\; \int _0^{|t|} \Vert \sigma (\psi _{\varphi _s})\Vert _{L^2}\, \text {d}s, \end{aligned}$$

and Lemma 3.8 yields $\sup _{|s|\leqslant T}\Vert \sigma (\psi _{\varphi _s})\Vert _{L^2}\leqslant C$, so $\sup _{|t|\leqslant T}\Vert \varphi _t\Vert _{L^2}\leqslant C(T)$. Moreover, for $t,t_0\in [-T,T]$,

5.31 $$\begin{aligned} \Vert \varphi _t-\varphi _{t_0}\Vert _{L^2} \;\leqslant \; \Vert (e^{-i\omega (t-t_0)}-1)\varphi _{t_0}\Vert _{L^2} \;+\; \int _{t_0}^{t} \Vert \sigma (\psi _{\varphi _s})\Vert _{L^2}\, \text {d}s, \end{aligned}$$

and both terms vanish as $t\rightarrow t_0$, hence $\varphi \in C([-T,T];L^2)$.

*Energy conservation.* We first note the Hellmann–Feynman identity

5.32 $$\begin{aligned} \dot{e}(\varphi _t)=\langle \psi _{\varphi _t},(\partial _t h_{\varphi _t})\psi _{\varphi _t}\rangle , \end{aligned}$$

which follows from differentiating the eigenvalue equation $(h_{\varphi _t}-e(\varphi _t))\psi _{\varphi _t}=0$,

5.33 $$\begin{aligned} (\partial _t h_{\varphi _t})\psi _{\varphi _t}+(h_{\varphi _t}-e(\varphi _t))\,\partial _t\psi _{\varphi _t}-\dot{e}(\varphi _t)\,\psi _{\varphi _t}=0, \end{aligned}$$

and taking the scalar product with $\psi _{\varphi _t}$. Using (5.32), we see that the aKG solution conserves the energy ${\mathcal {E}}_{\textrm{field}}$ defined in (1.21):

5.34 $$\begin{aligned} \frac{d}{dt}{\mathcal {E}}_{\textrm{field}}(\varphi _t) \, =\, \langle \psi _{\varphi _t}, V_{i \omega \varphi _t}\psi _{\varphi _t}\rangle + 2\text {Re}\langle \dot{\varphi }_t,\omega \varphi _t\rangle \, =\, 0. \end{aligned}$$

Indeed, inserting $\dot{\varphi }_t=-i(\omega \varphi _t+\sigma (\psi _{\varphi _t}))$ into the second term produces $2\text {Re}\langle \dot{\varphi }_t,\omega \varphi _t\rangle = - 2\text {Im}\langle \sigma (\psi _{\varphi _t}), \omega \varphi _t\rangle = - \langle \psi _{\varphi _t}, V_{i\omega \varphi _t} \psi _{\varphi _t} \rangle$, which cancels the first contribution.

*Continuation and alternative.* Let *I* be any open existence interval containing 0. By energy conservation and (3.46d), there exist 0<α<1 and $C_0>0$ such that

5.35 $$\begin{aligned} (1-\alpha )\,\Vert \varphi _t\Vert _{{\mathfrak {h}}_{1/2}}^2 \;\leqslant \; {\mathcal {E}}_{\textrm{field}}(\varphi _0)+C_0, \qquad \forall t\in I, \end{aligned}$$

hence $\sup _{t\in I}\Vert \varphi _t\Vert _{{\mathfrak {h}}_{1/2}}<\infty$. On any compact interval J⊂I with $\inf _{t\in J}\triangle (\varphi _t)>0$, Lemma 5.2 shows that $u\mapsto \psi _u$ is Lipschitz on $\{\varphi _t:t\in J\}$, and hence $u\mapsto \sigma (\psi _u)$ is locally Lipschitz in ${\mathfrak {h}}_{1/2}$. Together with the a priori bound (5.35), this allows the standard continuation argument to extend the solution beyond *J*. Therefore, if $T_+<\infty$ resp. $T_-<\infty$, the only obstruction to continuation is closing of the gap:

5.36 $$\begin{aligned} \liminf _{t\uparrow T_+}\triangle (\varphi _t)=0 \qquad \text {resp.} \qquad \liminf _{t\downarrow -T_-}\triangle (\varphi _t)=0 . \end{aligned}$$

This yields the continuation alternative and shows that the existence interval is maximal. □

In the remainder of this section, we prove estimates on the reduced resolvent

5.37 $$\begin{aligned} R_{\varphi _t} = Q_{\varphi _t} ( h_{\varphi _t} - e(\varphi _t) )^{-1} Q_{\varphi _t}, \end{aligned}$$

and derive an equation of motion for $t\mapsto \psi _{\varphi _t}$.

### Lemma 5.3

Let $\varphi _t$ denote the solution to (aKG) for initial data $\varphi _0\in {\mathfrak {h}}_{1/2}$ with $e(\varphi _0)<0$, and let $\psi _{\varphi _t}\geqslant 0$ be the non-negative normalized ground state of $h_{\varphi _t}$. Then, there exists T>0 such that the following statements hold for all |t|⩽T.

(i) There is C>0 such that
  5.38 $$\begin{aligned} \Vert R_{\varphi _t} \Vert + \Vert \langle p \rangle R_{\varphi _t}^{1/2} \Vert + \Vert \langle p^2 \rangle R_{\varphi _t} \Vert + \Vert \psi _{\varphi _t}\Vert _{H^2}&\leqslant C . \end{aligned}$$
(ii) The map $t\mapsto \psi _{\varphi _t}$ is in $C((-T,T);H^1) \cap C^1((-T,T);L^2)$ and
  5.39 $$\begin{aligned} \partial _t \psi _{\varphi _t} = R_{\varphi _t} V_{i\omega \varphi _t} \psi _{\varphi _t} . \end{aligned}$$
(iii) There is C>0 such that
  5.40 $$\begin{aligned} \Vert \dot{\psi }_{\varphi _t} \Vert _{L^2} + \Vert \dot{\sigma }( \psi _{\varphi _t} ) \Vert _{L^2 } + \Vert \dot{R}_{\varphi _t} \Vert + \Vert \langle p \rangle \dot{R}_{\varphi _t} \langle p \rangle \Vert&\leqslant C \ \end{aligned}$$
  where $\Vert \cdot \Vert$ denotes the operator norm on $L^2({\mathbb {R}}^3)$, and $\dot{f}(t):= \partial _t f(t)$.

### Proof

The first bound $\Vert R_{\varphi _t} \Vert \lesssim 1$ obviously follows from the spectral gap $\inf _{|t| \leqslant T } \triangle (\varphi _t)>0$. For the second bound, use $p^2 = h_{\varphi _t} - V_{\varphi _t}$ and $h_{\varphi _t} R_{\varphi _t} = Q_{\varphi _t} + e(\varphi _t) R_{\varphi _t}$ so that

5.41 $$\begin{aligned} R_{\varphi _t}^{1/2} \langle p \rangle ^2 R_{\varphi _t}^{1/2} = R_{\varphi _t}^{1/2} (1+ p^2 ) R_{\varphi _t}^{1/2} = R_{\varphi _t} (1+e(\varphi _t)) + Q_{\varphi _t} - R_{\varphi _t}^{1/2} V_{\varphi _t} R_{\varphi _t}^{1/2}, \end{aligned}$$

which we use to estimate

5.42 $$\begin{aligned} \Vert \langle p \rangle R_{\varphi _t}^{1/2} \Vert ^2 \lesssim 1 + \Vert R_{\varphi _t}^{1/2} \Vert \, \Vert \langle p \rangle R_{\varphi _t}^{1/2} \Vert \end{aligned}$$

where the last step follows from Lemma 3.7 (3.46a) as well as $|e(\varphi _t) | \lesssim 1$. This proves the second bound. We proceed similarly in the third bound, namely by writing $\langle p^2\rangle R_{\varphi _t} = R_{\varphi _t} (1+e(\varphi _t) ) +Q_{\varphi _t} - V_{\varphi _t} R_{\varphi _t}$, which we now estimate as

5.43 $$\begin{aligned} \Vert \langle p^2\rangle R_{\varphi _t} \Vert&\lesssim 1 + \Vert V_{\varphi _t} R_{\varphi _t} \Vert \lesssim 1 + \Vert \langle p\rangle R_{\varphi _t} \Vert \lesssim 1 \end{aligned}$$

where we used again (3.46a). For the last bound in (i), we proceed one more time in the same spirit, that is

5.44 $$\begin{aligned} \Vert \psi _{\varphi _t}\Vert _{H^2}^2 \leqslant 1 + \Vert p^2 \psi _{\varphi _t} \Vert ^2_{L^2} \leqslant 1 + |e(\varphi _t)| + \Vert V_{\varphi _t} \psi _{\varphi _t} \Vert _{L^2}^2 \leqslant 1 + \Vert \psi _{\varphi _t} \Vert ^2_{H^1}. \end{aligned}$$

That $\Vert \psi _{\varphi _t}\Vert _{H^1} \lesssim 1$ follows as in (5.23). This proves the first statement.

Regarding the second statement, note that continuity and differentiability of $t\mapsto \psi _{\varphi _t}$ follow directly from Lemma 5.2 in combination with continuity and differentiability of $t\mapsto \varphi _t$. Moreover, observe that $\langle \psi _{\varphi _t},\partial _t\psi _{\varphi _t}\rangle = 2\text {Re}\langle \psi _{\varphi _t},\partial _t\psi _{\varphi _t}\rangle = \partial _t \Vert \psi _{\varphi _t}\Vert ^2 =0$.

To obtain (5.39), we use the Hellmann–Feynman identity (5.32) and $Q_{\varphi _t}\psi _{\varphi _t}=0$, which yields

5.45 $$\begin{aligned} Q_{\varphi _t}(h_{\varphi _t}-e(\varphi _t)) Q_{\varphi _t} \partial _t\psi _{\varphi _t} =-\,Q_{\varphi _t}(\partial _t h_{\varphi _t})\,\psi _{\varphi _t}. \end{aligned}$$

By uniform positivity of the spectral gap, the operator $h_{\varphi _t}-e(\varphi _t)$ is invertible on $\text {Ran}(Q_{\varphi _t})$ with bounded inverse $R_{\varphi _t}$, and thus

5.46 $$\begin{aligned} \partial _t\psi _{\varphi _t}=-\,R_{\varphi _t}(\partial _t h_{\varphi _t})\,\psi _{\varphi _t}. \end{aligned}$$

Since $h_{\varphi }=p^2+V_{\varphi }$ depends linearly on φ, we have $\partial _t h_{\varphi _t}=V_{\partial _t\varphi _t}$. Along the aKG flow, we have $\partial _t\varphi _t=-\,i\omega \varphi _t - i \sigma (\psi _{\varphi _t})$, hence $\partial _t h_{\varphi _t}=-\,V_{i\omega \varphi _t} -V_{ i \sigma (\psi _{\varphi _t})}$. Since $\overline{\sigma (\psi _{\varphi _t})(k)} = \sigma (\psi _{\varphi _t})(-k)$, we find $V_{ i \sigma (\psi _{\varphi _t})} = 0$, and thus $\partial _t \psi _{\varphi _t} = R_{\varphi _t} V_{i\omega \varphi _t}\psi _{\varphi _t}$.

For the third statement, note that the bound on $\Vert \dot{\psi }_{\varphi _t}\Vert _{L^2}$ was derived already in the proof of (ii). For the second bound, we compute

5.47 $$\begin{aligned} \dot{\sigma }(\psi _{\varphi _t})(k) = \langle R_{\varphi _t} V_{i\omega \varphi _t} \psi _{\varphi _t}, {{\textbf {G}}} (\cdot , k) \psi _{\varphi _t} \rangle + \langle \psi _{\varphi _t} , {{\textbf {G}}} (\cdot , k) R_{\varphi _t} V_{i\omega \varphi _t} \psi _{\varphi _t} \rangle . \end{aligned}$$

Both terms are estimated in analogy to the proof of (3.53), which yields

5.48 $$\begin{aligned} | \langle R_{\varphi _t} V_{i\omega \varphi _t} \psi _{\varphi _t}, {{\textbf {G}}} (\cdot , k) \psi _{\varphi _t}\rangle | \lesssim \Vert R_{\varphi _t} V_{i\omega \varphi _t} \psi _{\varphi _t} \Vert _{H^1} \, \Vert \psi _{\varphi _t}\Vert _{H^1} \lesssim 1. \end{aligned}$$

For the last bound, observe that $\dot{Q}_{\varphi _t} = - \dot{P}_{\varphi _t} = P_{\varphi _t} V_{i\omega \varphi _t} R_{\varphi _t} - R_{\varphi _t} V_{i\omega \varphi _t} P_{\varphi _t}$ with $P_{\varphi _t} = | \psi _{\varphi _t} \rangle \langle \psi _{\varphi _t}|$ to compute

5.49 $$\begin{aligned} \dot{R}_{\varphi _t}&= P_{\varphi _t} V_{i\omega \varphi _t} R_{\varphi _t}^2 - R_{\varphi _t}^2 V_{i\omega \varphi _t} P_{\varphi _t} - R_{\varphi _t} ( \dot{h}_{\varphi _t} - \dot{e}(\varphi _t)) R_{\varphi _t}. \end{aligned}$$

The bound $\Vert \dot{R}_{\varphi _t} \Vert + \Vert \langle p \rangle \dot{R}_{\varphi _t} \langle p \rangle \Vert \leqslant C$ now follows from Lemma 3.7 (3.46a) in combination with the resolvent bounds from Item (i), as well as the estimate

5.50 $$\begin{aligned} \langle p\rangle ^{-1} ( \dot{h}_{\varphi _t} - \dot{e}(\varphi _t)) \langle p\rangle ^{-1}&= \langle p\rangle ^{-1} \left( V_{i\omega \varphi _t} - \langle \psi _{\varphi _t} , V_{i\omega \varphi _t } \psi _{\varphi _t} \rangle \right) \langle p \rangle ^{-1} \lesssim 1 \end{aligned}$$

where the last step is a consequence of the form bound $\pm V_{\varphi } \leqslant C \langle p^2\rangle \Vert \varphi \Vert _{{\mathfrak {h}}_{1/2}}$ from (3.46a). This completes the proof of the lemma. □

## Renormalization of the quantum fluctuations

In this section, we construct the *renormalized Bogoliubov–Nelson evolution*, which describes quantum field fluctuations around the classical aKG field. Our main goal is to prove Proposition 1.4, derive important properties of the unitary propagator ${\mathbb {U}}(t)$, and obtain an explicit expression for its quadratic generator. The proof relies on two technical lemmas.

The first lemma provides a representation of the quadratic operator introduced in (1.27),

6.1 $$\begin{aligned} {\mathbb {H}}_\Lambda (t) = H_f - \langle \psi _{\varphi _t} \phi ( {{\textbf {G}}}_\Lambda ) R_{\varphi _t} \phi ( {{\textbf {G}}}_\Lambda ) \psi _{\varphi _t} \rangle , \end{aligned}$$

in terms of a quadratic operator ${\mathbb {H}}_{{\mathscr {D}}, K,\Lambda }(t)$ with cutoffs $2 \leqslant K\le \Lambda \leqslant \infty$ and an explicit energy shift. This representation is analogous to (4.1), but with the important distinction that no dressing transformation is required to relate ${\mathbb {H}}_\Lambda (t)$ and ${\mathbb {H}}_{{\mathscr {D}}, K,\Lambda }(t)-4\pi \ln \Lambda$. We nevertheless refer to ${\mathbb {H}}_{{\mathscr {D}}, K,\Lambda }(t)$ as the dressed Bogoliubov–Nelson Hamiltonian, since it corresponds to the Bogoliubov type approximation for the dressed Nelson Hamiltonian $H_{{\mathscr {D}},K}^\varepsilon$. We will show that the operators ${\mathbb {H}}_{{\mathscr {D}}, K,\Lambda }(t)$ have a limit as $\Lambda \rightarrow \infty$.

The second lemma provides the necessary bounds for ${\mathbb {H}}_{{\mathscr {D}},K,\Lambda }(t)$ to prove the existence of the corresponding unitary evolution. These results together constitute the main ingredients to prove Proposition 1.4.

For Λ>0, we define the cutoff version of ${{\textbf {B}}}_K$ by

6.2 $$\begin{aligned} {{\textbf {B}}}_{K,\Lambda }(x,k)&={{\textbf {B}}}_K(x,k) {\textbf{1}}_{|k|\le \Lambda } = \frac{e^{-ikx}}{\omega (k)^{\frac{1}{2}}|k|^{2}}\,{\textbf{1}}_{K\le |k|\le \Lambda }= \frac{e^{-ikx}}{|k|^{\frac{5}{2}}}\,{\textbf{1}}_{K\le |k|\le \Lambda }\,. \end{aligned}$$

The parameter *K* plays the same role as the cutoff appearing in Lemma 4.1. Throughout this section, all generators and evolutions are defined relative to $\varphi _t$, a solution to (aKG) for initial data $\varphi _0 \in {\mathfrak {h}}_{1/2}$ with $e(\varphi _t)<0$, as described in the beginning of Sect. 1.3.

We then introduce the dressed Bogoliubov–Nelson Hamiltonian

6.3 $$\begin{aligned} {\mathbb {H}}_{{\mathscr {D}},K,\Lambda }(t)&= H_f + \big \langle \psi _{\varphi _t},\, \big ( D_{K,\Lambda } + 2 \text {Re}\langle \sigma (\psi _{\varphi _t}), {{\textbf {B}}}_{K,\Lambda }\rangle - A_{K,\Lambda } R_{\varphi _t} A_{K,\Lambda } \big ) \psi _{\varphi _t} \big \rangle _{L^2}\,, \end{aligned}$$

6.4 $$\begin{aligned} A_{K,\Lambda }&= \phi ({{\textbf {G}}}_K) + 2p\cdot a(k {{\textbf {B}}}_{K,\Lambda }) + 2a^*({{\textbf {k}}} B_{K,\Lambda })\cdot p , \end{aligned}$$

6.5 $$\begin{aligned} D_{K,\Lambda }&= 2 a^*(k {{\textbf {B}}}_{K,\Lambda })\, a(k {{\textbf {B}}}_{K,\Lambda }) + a^*(k {{\textbf {B}}}_{K,\Lambda })^2 + a(k {{\textbf {B}}}_{K,\Lambda })^2 + 4\pi \ln K\,. \end{aligned}$$

Observe that $A_{K,\infty }=A_K$ and $D_{K,\infty }=D_K$, as defined earlier in (3.28) and (3.29).

As in the definition of ${\mathbb {H}}_\Lambda (t)$ in (1.27), the form of ${\mathbb {H}}_{{\mathscr {D}},K,\Lambda }(t)$ is motivated by second-order perturbation theory around the adiabatic subspace ${\mathcal {V}}(t):=\{\psi _{\varphi _t}\otimes \Theta :\Theta \in {\mathcal {F}}\}$ and by the corresponding Feshbach–Schur complement. Here, however, we apply the same formal prescription to the dressed Hamiltonian $H_{{\mathscr {D}},K}^{\varepsilon }$, rather than $H^{\varepsilon }_\Lambda$, and impose the additional momentum restriction $|k|\le \Lambda$. More precisely, the operator $\varepsilon ^2 {\mathbb {H}}_{{\mathscr {D}},K,\Lambda }(t)$ consists of all contributions from the Feshbach–Schur complement of ${\mathscr {H}}^{\varepsilon }_{{\mathscr {D}},K,\varphi _t}$, defined as in (4.7) but with the additional restriction $|k|\le \Lambda$, that are of order $\varepsilon ^2$ and quadratic in creation and annihilation operators.

The next lemma shows that the form domain of ${\mathbb {H}}_{{\mathscr {D}}, K,\Lambda }(t)$ always contains the dense subspace $Q(H_f) \subset {\mathcal {F}}$, for all $2 \leqslant K < \Lambda \leqslant \infty$. In particular, setting $\Lambda =\infty$ does not alter the domain. Moreover, it provides the key identity (in statement (ii)) and estimates (in statement (iii)) for the renormalization of the fluctuation dynamics.

### Lemma 6.1

Let ${\mathbb {H}}_{\Lambda }(t)$ and ${\mathbb {H}}_{{\mathscr {D}},K,\Lambda }(t)$ be defined by (1.27) and (6.3), respectively, both relative to the aKG solution $\varphi _t$ with initial data $\varphi _0\in {\mathfrak {h}}_{1/2}$ satisfying $e(\varphi _0)<0$. There exists T>0 such that the following statements hold:

(i) There exists C>0 such that for all |t|⩽T, in the sense of quadratic forms,
  6.6 $$\begin{aligned} \pm {\mathbb {H}}_{\Lambda }(t)&\le C\,\Lambda ^2\, H_f + C \qquad&\text {for all }\quad&1<\Lambda <\infty , \end{aligned}$$
  6.7 $$\begin{aligned} \pm {\mathbb {H}}_{{\mathscr {D}} , K,\Lambda }(t)&\le C\,K^2\, H_f + C\,\ln K \qquad&\text {for all } \quad&2\le K<\Lambda \le \infty . \end{aligned}$$
(ii) As quadratic forms on $Q(H_f)$, we have the identity for all |t|⩽T
  6.8 $$\begin{aligned} {\mathbb {H}}_{{\mathscr {D}} , K,\Lambda }(t) = {\mathbb {H}}_\Lambda (t) + 4\pi \ln \Lambda \qquad \text {for all } \quad 2\leqslant K<\Lambda <\infty \,. \end{aligned}$$
(iii) There is C>0 such that, for all $2\leqslant K<\Lambda <\infty$, |t|⩽T and all normalized $\Psi ,\Phi \in Q(H_f)$,
  6.9 $$\begin{aligned} \big | \big \langle \Psi ,\, \big ( {\mathbb {H}}_{{\mathscr {D}},K,\infty }(t) - {\mathbb {H}}_{{\mathscr {D}},K,\Lambda }(t) \big )\Phi \big \rangle \big | \le \frac{C\,K^{1/2}}{\Lambda ^{1/4}} \Big ( \big \langle \Psi , H_f \Psi \big \rangle + \big \langle \Phi , H_f \Phi \big \rangle + 1 \Big )\,. \end{aligned}$$

We refer to ${\mathbb {H}}_{{\mathscr {D}},K}(t):= {\mathbb {H}}_{{\mathscr {D}},K,\infty } (t)$ as the *renormalized Bogoliubov–Nelson Hamiltonian*.

### Proof of (i):

The first bound follows from (3.32) and $\Vert R_{\varphi _t} \Vert \lesssim 1$. For the second bound, we apply Lemma 3.5 in combination with $\Vert \langle p \rangle R_{\varphi _t} \langle p \rangle \Vert \lesssim 1$, see Lemma 5.3, and $\Vert \langle p \rangle P_{\varphi _t} \Vert \lesssim 1$. Note that Lemma 3.5 was stated only for $\Lambda =\infty$, but the proofs generalize trivially to finite values of Λ. □

### Proof of (ii):

For notational simplicity, let us ignore the *t*-dependence and write $h:=h_{\varphi _t}$, $e:= e(\varphi _t)$, $R:= R_{\varphi _t}$ and (h-e)R=Q=1-P with $P=|\psi \rangle \langle \psi |$. In fact, the following argument relies only on these relations together with (h-e)ψ=0. Let us also introduce the anti-hermitian field operator $\phi ^-(f):= a^*(f) - a(f)$, and for better readability, let us abbreviate ${{\textbf {B}}}:= {{\textbf {B}}}_{K,\Lambda }$ within this computation.

We start by noting that $2 a^*(k {{\textbf {B}}} ) a( k {{\textbf {B}}} ) + a^*( k {{\textbf {B}}} ) a^*( k {{\textbf {B}}} ) + a( k {{\textbf {B}}}) a( k {{\textbf {B}}} ) = \phi ( k {{\textbf {B}}} )^2 - \Vert k {{\textbf {B}}}\Vert ^2_{L^2}$ and write, using $\phi (k {{\textbf {B}}}) = - [p, \phi ^- ({{\textbf {B}}}) ]$,

$$\begin{aligned} \Big [ [ p^2 , \phi ^-({{\textbf {B}}} ) ] , \phi ^- ({{\textbf {B}}}) \Big ] = 2 ([p , \phi ^- ({{\textbf {B}}}) ])^2 - p [ \phi (k {{\textbf {B}}}) , \phi ^-({{\textbf {B}}}) ] - [ \phi (k {{\textbf {B}}}) , \phi ^-({{\textbf {B}}}) ] p. \end{aligned}$$

The last two terms on the right side vanish, since $$[ \phi (k {{\textbf {B}}}), \phi ^-({{\textbf {B}}}) ] = 2\text {Re}\langle {{\textbf {B}}}, k {{\textbf {B}}} \rangle =0$$. On the left side we can replace $$p^2 $$ by h-e in the commutator (since multiplication operators in *x* commute with $$\phi ^-({{\textbf {B}}})$$). Because $$[p,\phi ^-({{\textbf {B}}})] = \phi (k{{\textbf {B}}})$$, this leads to the identity

6.10 $$\begin{aligned} \phi (k {{\textbf {B}}} )^2&= \frac{1}{2}\Big [ \big [ h-e , \phi ^- ({{\textbf {B}}}) \big ] , \phi ^- ({{\textbf {B}}} ) \Big ] \end{aligned}$$

and therefore, taking the partial expectation in $\psi \in L^2({\mathbb {R}}^3_x)$ and using (h-e)ψ=0,

6.11 $$\begin{aligned} \big \langle \psi , \phi ( k {{\textbf {B}}} )^2 \psi \big \rangle&= - \big \langle \psi , \phi ^- ({{\textbf {B}}} ) (h - e) \phi ^- ({{\textbf {B}}} ) \psi \big \rangle . \end{aligned}$$

On the other hand, we use

6.12 $$\begin{aligned} 2 p \cdot a(k {{\textbf {B}}} ) + 2 a^*( k {{\textbf {B}}} ) \cdot p&= - \big [ h - e , \phi ^- ( {{\textbf {B}}} ) \big ] - \phi ( k^2 {{\textbf {B}}}) \end{aligned}$$

together with the identity ${{\textbf {G}}}_K - k^2 {{\textbf {B}}} = {{\textbf {G}}}_K - k^2 {{\textbf {B}}}_{K,\Lambda } = {{\textbf {G}}}_{\Lambda }$ and P(h-e)=0, so that

6.13 $$\begin{aligned} P A_{K,\Lambda } Q&= P \phi ({{\textbf {G}}}_{\Lambda } ) Q + P \phi ^- ( {{\textbf {B}}} ) (h-e) Q , \end{aligned}$$

6.14 $$\begin{aligned} Q A_{K,\Lambda } P&= Q \phi ({{\textbf {G}}}_{\Lambda } ) P - Q (h-e) \phi ^- ( {{\textbf {B}}} ) P. \end{aligned}$$

As a result, we have with $R = Q(h-e)^{-1}Q$:

6.15 $$\begin{aligned} \big \langle \psi , A_{K,\Lambda } R A_{K,\Lambda } \psi \big \rangle&= \langle \psi , \phi ({{\textbf {G}}}_{\Lambda } ) R \phi ({{\textbf {G}}}_{\Lambda } ) \psi \rangle + \langle \psi , \phi ^-( {{\textbf {B}}} ) Q \phi ({{\textbf {G}}}_{ \Lambda } ) \psi \rangle \nonumber \\&\quad - \langle \psi , \phi ({{\textbf {G}}}_{ \Lambda } ) Q \phi ^- ( {{\textbf {B}}} ) \psi \rangle - \langle \psi , \phi ^- ( {{\textbf {B}}} ) ( h - e ) \phi ^- ( {{\textbf {B}}} ) \psi \rangle . \end{aligned}$$

Next, write the second and third summands as

6.16 $$\begin{aligned}&\langle \psi , \phi ^-( {{\textbf {B}}} ) Q \phi ({{\textbf {G}}}_{ \Lambda } ) \psi \rangle - \langle \psi , \phi ({{\textbf {G}}}_{ \Lambda } ) Q \phi ^- ( {{\textbf {B}}} ) \psi \rangle \nonumber \\&\quad = \langle \psi , [ \phi ^-( {{\textbf {B}}}) , \phi ({{\textbf {G}}}_{ \Lambda } ) ] \psi \rangle - \langle \psi , \phi ^-( {{\textbf {B}}}) P \phi ({{\textbf {G}}}_{ \Lambda } ) \psi \rangle + \langle \psi , \phi ({{\textbf {G}}}_{ \Lambda } ) P \phi ^- ( {{\textbf {B}}} ) \psi \rangle \nonumber \\&\quad = -2 \text {Re}\langle {{\textbf {B}}} , {{\textbf {G}}}_\Lambda \rangle + 2 \text {Re}\langle \langle \psi , {{\textbf {G}}}_\Lambda \psi \rangle , \langle \psi , {{\textbf {B}}} \psi \rangle \rangle \nonumber \\&\quad = -2 \text {Re}\langle {{\textbf {B}}} , {{\textbf {G}}} \rangle + 2 \text {Re}\langle \sigma (\psi ) , \langle \psi , {{\textbf {B}}} \psi \rangle \rangle . \end{aligned}$$

Taking all the above into account we arrive at the claimed identity:

6.17 $$\begin{aligned} {{\mathbb {H}}}_{{\mathscr {D}},K,\Lambda }(t)&= {\mathbb {H}}_\Lambda (t) +4\pi \ln K - \Vert k {{\textbf {B}}} \Vert ^2_{L^2} + 2 \text {Re}\langle {{\textbf {B}}} , {{\textbf {G}}} \rangle = {\mathbb {H}}_\Lambda (t) + 4\pi \ln \Lambda , \end{aligned}$$

where we used $\langle {{\textbf {B}}}, 2 {{\textbf {G}}} - k^2 {{\textbf {B}}} \rangle = \langle {{\textbf {B}}}, {{\textbf {G}}} \rangle = \langle {{\textbf {B}}}_{K,\Lambda }, {{\textbf {G}}} \rangle = 4\pi \ln \Lambda - 4\pi \ln K$. This completes the proof of part (ii). □

### Proof of (iii):

Using ${{\textbf {B}}}_K - {{\textbf {B}}}_{K,\Lambda } = {{\textbf {B}}}_\Lambda$ with ${{\textbf {B}}}_\Lambda$ and ${{\textbf {B}}}_\Lambda$ defined as in (2.6), we can write

6.18a $$\begin{aligned} { {\mathbb {H}} }_{{\mathscr {D}}, K , \infty }(t) - { {\mathbb {H}} }_{{\mathscr {D}}, K,\Lambda }(t)&= \big \langle \psi _{\varphi _t}, ( D_{K} - D_{K,\Lambda } + 2 \text {Re} \langle \sigma ( \psi _{\varphi _t}) , {{\textbf {B}}}_\Lambda \rangle ) \psi _{\varphi _t} \big \rangle \end{aligned}$$

6.18b $$\begin{aligned}&\quad + \big ( \big \langle \psi _{\varphi _t}, ( A_{K,\Lambda } - A_{K} ) R_{\varphi _t} A_{K } \psi _{\varphi _t} \big \rangle + H.c.\big ) , \end{aligned}$$

where the scalar product denotes again the partial expectation in $L^2({\mathbb {R}}^3)$.

To bound the second line, note that $A_{K} -A_{K,\Lambda } = 2 a^*( k {{\textbf {B}}}_\Lambda ) \cdot p + 2 p\cdot a ( k {{\textbf {B}}}_{\Lambda } )$. Thus, we can use Cauchy–Schwarz and estimate via Lemma 3.5:

6.19 $$\begin{aligned} \langle \psi _{\varphi _t} , A_{K, \Lambda } R_{\varphi _t} A_{K,\Lambda } \psi _{\varphi _t} \big \rangle \leqslant C K^2 ( H_f +1 ) \end{aligned}$$

for all $\Lambda \in [K,\infty ]$, as well as via (3.33) and (3.35):

6.20 $$\begin{aligned}&\tfrac{1}{4} \langle \psi _{ \varphi _t } , ( A_{K} -A_{K,\Lambda } ) R_{\varphi _t} (A_{K} -A_{K,\Lambda } ) \psi _{\varphi _t} \rangle \nonumber \\&\quad \leqslant \langle \psi _{ \varphi _t } , ( a^*( k {{\textbf {B}}}_\Lambda ) \cdot p) R_{\varphi _t} ( p\cdot a ( k {{\textbf {B}}}_{\Lambda } ) ) \psi _{\varphi _t} \rangle + \langle \psi _{ \varphi _t } , ( p\cdot a ( k {{\textbf {B}}}_{\Lambda } ) ) R_{\varphi _t} ( a^*( k {{\textbf {B}}}_\Lambda )\cdot p) \psi _{\varphi _t} \rangle \nonumber \\&\quad \leqslant C \Lambda ^{-1} (H_f+1). \end{aligned}$$

Combining the above estimates, this gives for all $\Psi ,\Phi \in D(H_f^{1/2})$

6.21 $$\begin{aligned} | \langle \Psi , (6.18b) \Phi \rangle | \lesssim C K \Lambda ^{-\frac{1}{2}} ( \langle \Psi , \mathrm T\Psi \rangle + \langle \Phi , \mathrm T\Phi \rangle + 1 ). \end{aligned}$$

For (6.18a), we use again ${{\textbf {B}}}_K - {{\textbf {B}}}_{K,\Lambda } = {{\textbf {B}}}_\Lambda$ to get:

6.22 $$\begin{aligned}&D_{K} - D_{K,\Lambda } = 2 a^*(k {{\textbf {B}}}_{K}) a( k {{\textbf {B}}}_{\Lambda }) + 2 a^*(k {{\textbf {B}}}_{\Lambda }) a( k {{\textbf {B}}}_{K,\Lambda } ) \nonumber \\&\qquad \quad + \big ( a^*( k {{\textbf {B}}}_{K} ) a^*( k {{\textbf {B}}}_{\Lambda }) + a^*( k {{\textbf {B}}}_{\Lambda } ) a^*( k {{\textbf {B}}}_{K,\Lambda }) + H.c. \big ) . \end{aligned}$$

It then follows from the bounds in Lemma 3.4 that for all $\Psi ,\Phi \in D(H_f^{1/2})$

6.23 $$\begin{aligned} | \langle \Psi , ( D_{K} - D_{K,\Lambda } ) \Phi \rangle | \lesssim C (K \Lambda )^{-\frac{1}{4}} ( \langle \Psi , H_f \Psi \rangle + \langle \Phi , H_f \Phi \rangle + 1 ). \end{aligned}$$

The remaining term in (6.18a), is bounded using $\Vert \sigma ( \psi _{\varphi _t} ) \Vert _{L^2} \lesssim 1$, see Lemma 3.8, and $\Vert B_\Lambda \Vert _{L^2} \lesssim \Lambda ^{-1}$. This completes the proof of the lemma. □

To study the unitary evolutions generated by the quadratic operators ${\mathbb {H}}_{{\mathscr {D}}, K,\Lambda }( t)$, we need the following estimates.

### Lemma 6.2

Let ${\mathbb {H}}_{{\mathscr {D}},K,\Lambda }(t)$ be defined by (6.3), relative to the (aKG) solution $\varphi _t$ with initial data $\varphi _0\in {\mathfrak {h}}_{1/2}$ satisfying $e(\varphi _0)<0$. There are constants $T,C,K_0>0$, such that

6.24a $$\begin{aligned}&\tfrac{2}{3} H_f - C \ln K (N+1) \leqslant {{\mathbb {H}}}_{{\mathscr {D}} , K,\Lambda }(t) \leqslant \tfrac{3}{2} H_f + C \ln K , \end{aligned}$$

6.24b $$\begin{aligned}&\pm \partial _t {\mathbb {H}}_{{\mathscr {D}} , K,\Lambda }(t) \leqslant C K^2 \big ( H_f + 1 \big ) , \end{aligned}$$

6.24c $$\begin{aligned}&\pm i [ {\mathbb {H}}_{{\mathscr {D}},K,\Lambda }(t), N] \leqslant CK^2 ( H_f + 1 ) , \end{aligned}$$

for all |t|⩽T and $K_0 \leqslant K < \Lambda \leqslant \infty$.

### Proof

For the upper bound in (6.24a), use $- A_{K,\Lambda }R_{\varphi _t}A_{K,\Lambda } \leqslant 0$ and apply Lemma 3.5 (3.30c) to estimate $\pm D_K \leqslant \frac{1}{4} H_f + C \ln K$. Moreover, we estimate $|2 \text {Re}\langle \sigma (\psi _{\varphi _t}), B_{K,\Lambda } \rangle | \lesssim \Vert B_{K} \Vert _{L^2} \lesssim K^{-1}$. For the lower bound, we apply the same bound for the part involving $D_{K,\Lambda } + 2 \text {Re}\langle \sigma (\psi _{\varphi _t}), {{\textbf {B}}}_{K,\Lambda } \rangle$ and further estimate $- A_{K,\Lambda }R_{\varphi _t}A_{K,\Lambda } \gtrsim \ln K (N+1)$ by Lemma 3.5 (3.31a). (We note again that Lemma 3.5 was stated only for $\Lambda =\infty$, but the estimates extend trivially to all $\Lambda \geqslant K$.) This proves (6.24a).

Next, we compute the time derivative

6.25 $$\begin{aligned} \partial _t {\mathbb {H}}_{{\mathscr {D}},K,\Lambda }(t)&= 2 \text {Re}\big \langle \dot{\psi }_{\varphi _t} , \big ( D_{K,\Lambda } + 2 \text {Re}\langle \sigma ( \psi _{\varphi _t ) } , {{\textbf {B}}}_{K,\Lambda } \rangle - A_{K,\Lambda } R_{\varphi _t} A_{K,\Lambda } \big ) \psi _{\varphi _t} \big \rangle \nonumber \\&\quad + \big \langle \psi _{\varphi _t}, \big ( 2 \text {Re}\langle \dot{\sigma }( \psi _{\varphi _t} ) ,{{\textbf {B}}}_{K,\Lambda } \rangle - A_{K,\Lambda } \dot{R}_{\varphi _t} A_{K,\Lambda } \big ) \psi _{\varphi _t} \big \rangle . \end{aligned}$$

Let us explain the bound for the term involving $A_{K,\Lambda } R_{\varphi _t} A_{K,\Lambda }$. After taking the expectation value in a normalized state $\Phi \in {\mathcal {F}}$, one rewrites the inner product as

6.26 $$\begin{aligned} \big \langle \Psi , \langle \dot{\varphi }_{\psi _t} , A_{K,\Lambda } R_{\varphi _t} A_{K,\Lambda } \psi _{\varphi _t} \big \rangle \Psi \big \rangle = \langle \dot{\psi }_{\varphi _t} \otimes \Phi , ( A_{K,\Lambda } R_{\varphi _t} A_{K,\Lambda } ) \psi _{\varphi _t} \otimes \Phi \rangle \end{aligned}$$

where the inner product on the right side is now in $L^2\otimes {\mathcal {F}}$. Then, we use Cauchy–Schwarz and (3.30a) to obtain

6.27 $$\begin{aligned}&2 |\langle \dot{\psi }_{\varphi _t} \otimes \Phi , ( A_{K,\Lambda } R_{\varphi _t} A_{K,\Lambda } ) \psi _{\varphi _t} \otimes \Phi \rangle | \nonumber \\&\qquad \leqslant |\langle \dot{\psi }_{\varphi _t} \otimes \Phi , ( A_{K,\Lambda } R_{\varphi _t} A_{K,\Lambda } ) \dot{\psi }_{\varphi _t} \otimes \Phi \rangle | + |\langle \psi _{\varphi _t} \otimes \Phi , ( A_{K,\Lambda } R_{\varphi _t} A_{K,\Lambda } ) \psi _{\varphi _t} \otimes \Phi \rangle | \nonumber \\&\qquad \leqslant C K^2 (\Vert \langle p \rangle R_{\varphi _t} \langle p \rangle \Vert + \Vert R_{\varphi _t} \Vert ) \left( \Vert \langle p \rangle \dot{\psi }_{\varphi _t} \Vert _{L^2}^2 + \Vert \langle p \rangle \psi _{\varphi _t} \Vert _{L^2}^2 \right) \Vert \langle H_f^{1/2} \rangle \Phi \Vert ^2 \nonumber \\&\qquad \leqslant C K^2 \Vert \langle H_f^\frac{1}{2} \rangle \Phi \Vert ^2, \end{aligned}$$

where we used Lemmas 5.3 and 3.7 in the last step, in particular the bound $\Vert \langle p \rangle \dot{\psi }_{\varphi _t} \Vert _{L^2} = \Vert \langle p \rangle R_{\varphi _t} V_{i\omega \varphi _t} \psi _{\varphi _t} \Vert _{L^2} \leqslant C$. The other terms in the first line in (6.25) and also the first term in the second line are estimated in the same way. To this end, note that the diagonal bound (3.30c) implies $\Vert F^{-1/2} D_K F^{-1/2} \Vert \lesssim 1$ in operator norm with $F = K^{-1/2} (1+ H_f) + \ln K$, so that $|\langle \Psi , D_K \Phi \rangle | \lesssim \langle \Psi , F \Psi \rangle + \langle \Phi , F \Phi \rangle$ for all $\Psi ,\Phi \in D(H_f^{1/2})$. The remaining term involving $A_{K,\Lambda } \dot{R}_{\varphi _t} A_{K,\Lambda }$ is estimated via (3.30a) in combination with (5.40). This proves the bound for the time derivative.

For the commutator, we compute for $\Psi \in {\mathcal {F}}$

6.28 $$\begin{aligned} \langle \Psi , [ { {\mathbb {H}} }_{{\mathscr {D}},\Lambda ,K}(t) , N ] \Psi \rangle&= \langle \psi _{\varphi _t} \otimes \Psi , [D_{K,\Lambda } , N ] \psi _{\varphi _t} \otimes \Psi \rangle \nonumber \\&\quad - 2 \text {Re} \langle \psi _{\varphi _t} \otimes \Psi , [A_{K,\Lambda } , N ] R_{\varphi _t} A_{K,\Lambda } \psi _{\varphi _t} \otimes \Psi \rangle \end{aligned}$$

and then apply the commutator bounds from Lemma 3.5.

This completes the proof of the lemma. □

The next lemma shows that the unitary evolutions ${\mathbb {U}}_{K,\Lambda }(t)$ generated by ${\mathbb {H}}_{{\mathscr {D}}, K,\Lambda }(t)$ with initial time t=0 exist and that they have a limit ${\mathbb {U}}_{K,\Lambda }(t) \xrightarrow {\Lambda \rightarrow \infty } {\mathbb {U}}_{K,\infty }(t)$.

### Proposition 6.3

Let $\varphi _t$ denote the solution to (aKG) with initial data $\varphi _0$ satisfying $e(\varphi _0)<0$, and let ${\mathbb {H}}_{{\mathscr {D}},K,\Lambda }(t)$ be defined by (6.3) relative to $\varphi _t$. Then, there are $T,K_0>0$ such that the following statements hold:

(i) For all $K_0 \leqslant K < \Lambda \leqslant \infty$ and $\Psi \in D( H_f^{1/2} ) \subset {\mathcal {F}}$, there exists a unique solution $\Psi \in C([-T,T],{\mathcal {F}})\cap L^\infty ([-T,T],D(H_f^{1/2}))$ to the Cauchy problem
  6.29 $$\begin{aligned} {\left\{ \begin{array}{ll} \begin{aligned} &  i \partial _t \Psi (t) = {\mathbb {H}}_{{\mathscr {D}} , K,\Lambda } (t) \Psi (t) \\ &  \Psi (0) = \Psi \end{aligned} \end{array}\right. } \end{aligned}$$
  with the equation understood in $D((1+H_f)^{-1/2})$. The solution map $\Psi \mapsto \Psi (t)$ defines a unitary ${\mathbb {U}}_{K,\Lambda }(t)$ on ${\mathcal {F}}$, which is strongly continuous in *t*, and satisfies
  6.30 $$\begin{aligned} \langle {\mathbb {U}}_{K,\Lambda } (t) \Psi ,H_f {\mathbb {U}}_{K,\Lambda } (t) \Psi \rangle \leqslant C_K \langle \Psi , ( H_f + 1 ) \Psi \rangle \end{aligned}$$
  for all $\Lambda \in (K,\infty ]$, |t|⩽T, with some *K*-dependent constant $C_K$.
(ii) The strong limit
  6.31 $$\begin{aligned} s-\lim _{\Lambda \rightarrow \infty } {\mathbb {U}} _{K,\Lambda }(t) = {\mathbb {U}} _{K,\infty }(t) \end{aligned}$$
  exists for all $K\geqslant K_0$, |t|⩽T, is unitary, and $t\mapsto {\mathbb {U}} _{K,\infty }(t)$ is strongly continuous.

Before we prove this result, we use it to prove Proposition 1.4.

### Proof of Proposition 1.4

Observe that via the identity (6.8), we identify the strongly continuous evolution ${\mathbb {U}}_\Lambda (t)$ as

6.32 $$\begin{aligned} {\mathbb {U}}_\Lambda (t) = {\mathbb {U}}_{K,\Lambda }(t) \exp (it\, 4\pi \, \ln \Lambda ). \end{aligned}$$

Moreover, with (6.31), we immediately have ${\mathbb {U}}_\Lambda (t) \exp (-it 4\pi \ln \Lambda ) \xrightarrow { \Lambda \rightarrow \infty } {\mathbb {U}}_{K,\infty }(t) =: {\mathbb {U}}(t)$ for any choice of $K \in [K_0,\infty )$. This proves the statement of Proposition 1.4. □

### Proof of Proposition 6.3

The existence of the unitary evolution, strong continuity and the bound (6.30) follow from the abstract result [33, Theorem 8] applied to $H(t):= {\mathbb {H}}_{{\mathscr {D}}, K,\Lambda }(t)$, $A:= H_f + \ln K$ and B:=N+1. That the assumptions of [33, Theorem 8] are satisfied is precisely the statement of the first two bounds from Lemma 6.2.

To prove the convergence statement, one computes for $\Psi \in D(H_f^{1/2})$:

6.33 $$\begin{aligned}&\Vert ( {\mathbb {U}} _{K,\Lambda } (t) - {\mathbb {U}}_{K,\infty } (t)) \Psi \Vert ^2 = 2 \text {Re}\int _0^t \langle {\mathbb {U}}_{K,\Lambda } (s) \Psi , ({\mathbb {H}}_{{\mathscr {D}},K,\Lambda }(s) - {\mathbb {H}}_{{\mathscr {D}},K,\infty } (s) ) {\mathbb {U}}_{K,\infty }(s) \Psi \rangle \text {d} s \nonumber \\&\qquad \quad \leqslant \frac{C_K K^\frac{1}{2} }{\Lambda ^{1/4}} \int _0^t \bigg ( \Big \langle {\mathbb {U}}_{K,\Lambda } (s) \Psi , H_f {\mathbb {U}}_{K,\Lambda } (s) \Psi \Big \rangle + \Big \langle {\mathbb {U}}_{K,\infty } (s) \Psi , H_f {\mathbb {U}}_{K,\infty } (s) \Psi \Big \rangle + 1 \bigg )\text {d}s\nonumber \\&\qquad \quad \leqslant C_K |t| K^\frac{1}{2} \Lambda ^{-\frac{1}{4}}, \end{aligned}$$

where we applied Lemma 6.1 (iii) and (6.30). By density of $D(H_f^{1/2}) \subseteq {\mathcal {F}}$, this proves strong convergence ${\mathbb {U}}_{K,\Lambda } (t) \xrightarrow {\Lambda \rightarrow \infty } {\mathbb {U}}_{K,\infty }(t)$. □

As a final property of the renormalized Bogoliubov evolution, we need estimates for higher powers of the number operator.

### Lemma 6.4

Let ${\mathbb {U}}(t)$ be the unitary in Proposition 1.4, defined for t∈[-T,T]. For every $m\in {\mathbb {N}}$, there is a constant $C_m>0$ such that for all |t|⩽T,

6.34 $$\begin{aligned} {\mathbb {U}} (t)^* ( N +1 )^m {\mathbb {U}} (t)&\leqslant C_m (N+1)^m , \end{aligned}$$

in the sense of quadratic forms on ${\mathcal {F}}$.

### Proof

As in the proof of Proposition 1.4, we fix $K\geqslant K_0$ and write ${\mathbb {U}}(t)={\mathbb {U}}_{K,\infty }(t)$. We also abbreviate

6.35 $$\begin{aligned} B:=N+1, \qquad {\mathbb {H}}(t):={\mathbb {H}}_{{\mathscr {D}},K,\infty }(t), \end{aligned}$$

and decompose

6.36 $$\begin{aligned} {\mathbb {H}}(t)=H_f+s_K(t)+Q_K(t), \end{aligned}$$

where $s_K(t)$ collects all scalar terms and $Q_K(t)$ is the remaining quadratic part. Since $H_f$ and $s_K(t)$ commute with *N*, only $Q_K(t)$ contributes to commutators with functions of *B*.

The number-operator estimates from Lemma 3.5 (see (3.31a) and (3.31c)), together with the bounds on $\psi _{\varphi _t}$ and $R_{\varphi _t}$ from Lemma 5.3, imply

6.37 $$\begin{aligned} \left| \left\langle \Phi ,Q_K(t)\Phi \right\rangle \right| \leqslant C_K \left\langle \Phi ,B\Phi \right\rangle , \end{aligned}$$

uniformly for |t|⩽T. By polarization, possibly increasing $C_K$, we obtain

6.38 $$\begin{aligned} \left| \left\langle \Phi ,Q_K(t)\Psi \right\rangle \right| \leqslant C_K \left\| B^{1/2}\Phi \right\| \left\| B^{1/2}\Psi \right\| . \end{aligned}$$

For $m, L\in {\mathbb {N}}$, define the bounded increasing function

6.39 $$\begin{aligned} f_L(n):=\min \{(n+1)^m,(L+1)^m\}, \qquad F_L:=f_L(N). \end{aligned}$$

Let $\Pi _n$ denote the projection onto the *n*-particle sector in ${\mathcal {F}}$. Since $Q_K(t)$ is quadratic in creation and annihilation operators,

6.40 $$\begin{aligned} \Pi _\ell Q_K(t)\Pi _n=0 \qquad \text {unless} \qquad \ell -n\in \{-2,0,2\}. \end{aligned}$$

Let now Φ be a finite-particle vector and write $\Phi _n:=\Pi _n\Phi$. Since the number-preserving part of $Q_K(t)$ commutes with $F_L$, we get

6.41 $$\begin{aligned} \left| \left\langle \Phi , i[{\mathbb {H}}(t),F_L]\Phi \right\rangle \right|&= \left| \left\langle \Phi , i[Q_K(t),F_L]\Phi \right\rangle \right| \nonumber \\&\leqslant \sum _{r=\pm 2}\sum _{n\geqslant 0} \left| f_L(n+r)-f_L(n)\right| \left| \left\langle \Phi _{n+r},Q_K(t)\Phi _n \right\rangle \right| , \end{aligned}$$

with the convention that $\Phi _j=0$ for j<0 Next, we observe the elementary estimate

6.42 $$\begin{aligned} \left| f_L(n+r)-f_L(n)\right| (n+1) \leqslant C_m ( f_L(n+r)+f_L(n) ) , \qquad r=\pm 2, \end{aligned}$$

with a constant independent of *L*.

Moreover, by (6.38), we have for r=±2

6.43 $$\begin{aligned} \left| \left\langle \Phi _{n+r},Q_K(t)\Phi _n \right\rangle \right|&\leqslant C_K(n+1) \Vert \Phi _{n+r}\Vert \,\Vert \Phi _n\Vert . \end{aligned}$$

Combining (6.41), (6.42), and (6.43), we obtain

6.44 $$\begin{aligned} \left| \left\langle \Phi , i[{\mathbb {H}}(t),F_L]\Phi \right\rangle \right|&\leqslant C_{m,K} \sum _{r=\pm 2}\sum _{n\geqslant 0} \big (f_L(n+r)+f_L(n)\big ) \Vert \Phi _{n+r}\Vert \,\Vert \Phi _n\Vert \nonumber \\&\leqslant C_{m,K} \sum _{n\geqslant 0} f_L(n)\Vert \Phi _n\Vert ^2 \nonumber \\&= C_{m,K} \left\langle \Phi ,F_L\Phi \right\rangle . \end{aligned}$$

Here we used Cauchy’s inequality and shifted the summation index. Since the right-hand side is bounded by $C_{m,K}(L+1)^m\Vert \Phi \Vert ^2$, the commutator form in (6.44) extends by density to all vectors in ${\mathcal {F}}$, and the estimate remains valid for this extension.

We now pass to the dynamics. First let $\Psi \in D(H_f^{1/2})$ and set $\Phi (t):={\mathbb {U}}(t)\Psi$. Since $F_L$ is a bounded function of *N* and *N* commutes with $H_f$, $F_L$ preserves $D(H_f^{1/2})$. Hence the evolution equation from Proposition 6.3 justifies the following differentiation in the sense of quadratic forms

6.45 $$\begin{aligned} \left| \frac{\mathrm d}{\mathrm dt} \left\langle \Phi (t),F_L\Phi (t) \right\rangle \right|&= \left| \left\langle \Phi (t), i[{\mathbb {H}}(t),F_L]\Phi (t) \right\rangle \right| \leqslant C_{m,K} \left\langle \Phi (t),F_L\Phi (t) \right\rangle , \end{aligned}$$

where the last step is obtained by (6.44). Gronwall’s inequality then yields

6.46 $$\begin{aligned} \left\langle {\mathbb {U}}(t)\Psi ,F_L{\mathbb {U}}(t)\Psi \right\rangle \leqslant \exp (C_{m,K}|t|) \left\langle \Psi ,F_L\Psi \right\rangle \end{aligned}$$

for all $$\Psi \in D(H_f^{1/2})$$.

For fixed *L*, the estimate (6.46) extends from $D(H_f^{1/2})$ to all $\Psi \in {\mathcal {F}}$ by density and unitarity of ${\mathbb {U}}(t)$. Finally, let $\Psi \in D(B^{m/2})$. Since $F_L\uparrow B^m$ as $L\rightarrow \infty$, monotone convergence gives

6.47 $$\begin{aligned} \left\langle {\mathbb {U}}(t)\Psi ,B^m{\mathbb {U}}(t)\Psi \right\rangle \leqslant \exp (C_{m,K}|t|) \left\langle \Psi ,B^m\Psi \right\rangle . \end{aligned}$$

Since *K* is fixed, we absorb the dependence on *K* into the constant. Thus

6.48 $$\begin{aligned} {\mathbb {U}}(t)^*(N+1)^m{\mathbb {U}}(t) \leqslant C_m(N+1)^m \end{aligned}$$

as quadratic forms, uniformly for |t|⩽T. □

## Proof of the norm approximation

In this section we prove Theorem 2.1. We fix the initial field $\varphi _0\in {\mathfrak {h}}_{1/2}$ with $e(\varphi _0)<0$. Given $\varphi _0$, we choose T>0 small enough so that the aKG dynamics and the Bogoliubov–Nelson evolution are well-defined on [-T,T], and so that all previously established estimates hold uniformly for |t|⩽T. For notational convenience, we restrict to t∈[0,T] in the proof; the argument extends verbatim to t∈[-T,0].

After recalling the precise goal, introducing some notation, and stating a preparatory lemma, the proof proceeds in three steps: a first-order Duhamel expansion in Sect. 7.1, followed by two adiabatic perturbation expansions in Sects. 7.2 and 7.3.

Our aim is to estimate the norm of the difference

7.1 $$\begin{aligned} {{\widetilde{\delta }}}(t) :=\; e^{i\varepsilon ^{-2} \mu (t)}\,W(\varepsilon ^{-1}\varphi _t)^* \,e^{-it \varepsilon ^{-2} H^{\varepsilon }}\,W(\varepsilon ^{-1}\varphi _0)\,\psi _{\varphi _0}\otimes \Omega \;-\; \psi _{\varphi _t}\otimes {\mathbb {U}}(t)\Omega , \end{aligned}$$

where $e^{-it \varepsilon ^{-2} H^\varepsilon }$ and ${\mathbb {U}}(t)$ are the renormalized propagators from Propositions 1.1 and 1.4, respectively, and $(\varphi _t,\psi _{\varphi _t})$ is the aKG solution pair from Proposition 1.2 for initial data $\varphi _0\in {\mathfrak {h}}_{1/2}$ with $e(\varphi _0)<0$. The phase is

7.2 $$\begin{aligned} \mu (t)=\int _0^t\Big (\text {Im}\langle \varphi _s,\partial _s\varphi _s\rangle +\Vert \omega ^{1/2}\varphi _s\Vert _{L^2}^2+e(\varphi _s)\Big )\, \text {d}s, \end{aligned}$$

and, for convenience, we recall the effective equations

7.3 $$\begin{aligned} i \partial _t {\mathbb {U}}(t)\Omega&= {\mathbb {H}}_{{\mathscr {D}},K,\infty }(t)\,{\mathbb {U}}(t)\Omega , \qquad i \partial _t \varphi _t = \omega \varphi _t + \sigma (\psi _{\varphi _t}), \qquad \partial _t \psi _{\varphi _t} = R_{\varphi _t} V_{i\omega \varphi _t}\psi _{\varphi _t}. \end{aligned}$$

The generator ${\mathbb {H}}_{{\mathscr {D}},K,\infty }(t)$ is defined in (6.3) relative to $\varphi _t$, for $K\geqslant K_0$ and some ε-independent $K_0>0$. We may choose any $K\geqslant K_0$. Finally, recall that $(h_{\varphi _t}-e(\varphi _t))\psi _{\varphi _t}=0$.

Instead of estimating ${{\widetilde{\delta }}}(t)$ directly, it is convenient to estimate the modified difference

7.4 $$\begin{aligned} \delta (t):=\xi _K(t)\;-\;\psi _{\varphi _t}\otimes {\mathbb {U}}(t)\Omega , \end{aligned}$$

with

7.5 $$\begin{aligned} \xi _K(t):=e^{i\varepsilon ^{-2}\mu (t)}\,U_K^\varepsilon \,W(\varepsilon ^{-1}\varphi _t)^* \,e^{-it\varepsilon ^{-2}H^\varepsilon }\,W(\varepsilon ^{-1}\varphi _0)\,(U_K^\varepsilon )^*\,\psi _{\varphi _0}\otimes \Omega , \end{aligned}$$

where we inserted the unitary dressing transformation

7.6 $$\begin{aligned} U_K^\varepsilon =\exp \!\Big (\varepsilon \, a^*({\boldsymbol{B}}_K) - \varepsilon \, a({\boldsymbol{B}}_K)\Big ), \qquad {\boldsymbol{B}}_K(x,k)=\frac{{\boldsymbol{G}}(x,k)}{k^2}\,{\textbf{1}}_{\{|k|\geqslant K\}}. \end{aligned}$$

Using

7.7 $$\begin{aligned} \Vert ( U_K^\varepsilon -{\textbf{1}})\Psi \Vert \;\lesssim \; \varepsilon \,\Vert B_K\Vert _{L^2}\,\Vert (N+1)^{1/2}\Psi \Vert \quad \text {for all}\quad \Psi \in D(N^{1/2}), \end{aligned}$$

together with $\Vert B_K\Vert _{L^2}\lesssim K^{-1}$ and Lemma 6.4, one readily verifies

7.8 $$\begin{aligned} \Vert (U_K^\varepsilon )^*\delta (t)-{{\widetilde{\delta }}}(t)\Vert&\leqslant \Vert ((U_K^\varepsilon )^*-{\textbf{1}})\,\psi _{\varphi _0}\otimes \Omega \Vert + \Vert ((U_K^\varepsilon )^*-{\textbf{1}})\,\psi _{\varphi _t}\otimes {\mathbb {U}}(t)\Omega \Vert \;\lesssim \; \varepsilon K^{-1} \end{aligned}$$

for all |t|⩽T. Hence $\Vert {{\widetilde{\delta }}}(t)\Vert \lesssim \Vert \delta (t)\Vert +\varepsilon K^{-1}$. At the end of the proof we shall set $K= \varepsilon ^{-4/5}$, so in what follows we focus on estimating δ(t).

Since $$U_K^\varepsilon W(\varepsilon ^{-1}\varphi _t)^*=W(\varepsilon ^{-1}\varphi _t)^* U_K^\varepsilon e^{-ig_{K,\varphi _t}(x)}$$ with

$$\begin{aligned} g_{K,\varphi _t}(x)=2\,\text {Im}\langle \varphi _t,{\boldsymbol{B}}_K(x,\cdot )\rangle , \end{aligned}$$

and $U_K^\varepsilon H^\varepsilon (U_K^\varepsilon )^*=H^\varepsilon _{{\mathscr {D}},K}$ (see Lemma 4.1), we can write

7.9 $$\begin{aligned} \xi _K(t)=e^{i\varepsilon ^{-2}\mu (t)}\,e^{-ig_{K,\varphi _t}(x)}\,W(\varepsilon ^{-1}\varphi _t)^*\,e^{-it\varepsilon ^{-2}H^\varepsilon _{{\mathscr {D}},K}}\, W(\varepsilon ^{-1}\varphi _0)\,e^{ig_{K,\varphi _0}(x)}\,\psi _{\varphi _0}\otimes \Omega \end{aligned}$$

As a final preparation, let us state and prove the following energy propagation bound.

### Lemma 7.1

Let $\xi _K(t)$ be given by (7.9). There exist C,T>0 and $K_0>2$ such that

7.10 $$\begin{aligned} \Big \Vert \Big ( p^2 + \varepsilon ^2 H_f \Big )^{1/2} \xi _K(t) \Big \Vert \leqslant C K^\frac{1}{2} \end{aligned}$$

for all |t|⩽T, ε>0 and $K\geqslant K_0$.

### Proof

We estimate

7.11 $$\begin{aligned}&\hspace{-0.4cm} \Big \Vert \Big ( p^2 + \varepsilon ^2 H_f \Big )^{1/2} \xi _K(t) \Big \Vert \nonumber \\&= \Big \Vert \Big ( p^2 + \varepsilon ^2 H_f \Big )^{1/2} e^{ - i g_{K,\varphi _t} (x)} W( \varepsilon ^{-1} \varphi _t) ^* e^{-i t \varepsilon ^{-2} H^{\varepsilon }_{{\mathscr {D}},K}} W(\varepsilon ^{-1} \varphi _0 ) e^{ i g_{K,\varphi _0} (x)} \psi _{\varphi _0} \otimes \Omega \Big \Vert \nonumber \\&\lesssim \Big \Vert \Big ( p^2 + \varepsilon ^2H_f + 1 \Big )^{1/2} e^{-i t \varepsilon ^{-2} H^{ \varepsilon }_{{\mathscr {D}},K} } W(\varepsilon ^{-1} \varphi _0 ) e^{ i g_{K,\varphi _0} (x)} \psi _{\varphi _0} \otimes \Omega \Big \Vert \end{aligned}$$

where we used

7.12 $$\begin{aligned} e^{i g_{K,\varphi _t}(x)} p^2 e^{-i g_{K,\varphi _t}(x)}&= p^2 + p \cdot (\nabla g_{K,\varphi _t} )(x) + (\nabla g_{K,\varphi _t} )(x) \cdot p + | ( \nabla g_{K,\varphi _t} )(x)|^2 \nonumber \\&\leqslant 2 p^2 + 2 | (\nabla g_{K,\varphi _t} )(x) |^2,\nonumber \\ W( \varepsilon ^{-1} \varphi _t) \varepsilon ^2 H_f W( \varepsilon ^{-1} \varphi _t) ^*&= \varepsilon ^2 H_f + \varepsilon \phi (\omega \varphi _t) + \Vert \varphi _t\Vert _{{\mathfrak {h}}_{1/2}}^2 \leqslant 2 \varepsilon ^2 H_f +2 \Vert \varphi _t\Vert _{{\mathfrak {h}}_{1/2}}^2 , \end{aligned}$$

and the bounds $\Vert \varphi _t \Vert _{{\mathfrak {h}}_{1/2}}^2 \lesssim 1$ and

7.13 $$\begin{aligned} | (\nabla g_{K,\varphi _t}) (x) | = 2 | \langle \varphi _t , k {{\textbf {B}}}(x,\cdot ) \rangle | \leqslant \Vert \varphi _t \Vert _{{\mathfrak {h}}_{1/2}} \Vert \omega ^{1/2} B_K \Vert _{L^2} \lesssim 1 \end{aligned}$$

for all |t|⩽T. Now apply (4.3), commute the unitary propagator with its generator, and apply (4.3) again:

7.14 $$\begin{aligned} \Big \Vert \Big ( p^2 + \varepsilon ^2 H_f \Big )^{1/2} \xi _K(t) \Big \Vert&\lesssim \Big \Vert \Big ( H^\varepsilon _{{\mathscr {D}} , K} + K \Big )^{1/2} W(\varepsilon ^{-1} \varphi _0 ) e^{ i g_{K,\varphi _0} (x)} \psi _{\varphi _0} \otimes \Omega \Big \Vert \nonumber \\&\lesssim \Big \Vert \Big (p^2 + \varepsilon ^2 H_f+K \Big ) W(\varepsilon ^{-1} \varphi _0 ) e^{ i g_{K,\varphi _0} (x)} \psi _{\varphi _0} \otimes \Omega \Big \Vert \nonumber \\&\lesssim \Big \Vert \Big ( p^2 + \varepsilon ^2 H_f + K \Big )^{1/2} \psi _{\varphi _0} \otimes \Omega \Big \Vert \nonumber \\&\lesssim K^\frac{1}{2} . \end{aligned}$$

This proves the lemma. □

### First-order expansion

We note the identity

7.15 $$\begin{aligned} i\partial _t W(\varepsilon ^{-1}\varphi _t)^*=\Big (-\varepsilon ^{-1}\phi (i\dot{\varphi }_t)+\varepsilon ^{-2}\text {Im}\langle \varphi _t,\dot{\varphi }_t\rangle \Big )W(\varepsilon ^{-1}\varphi _t)^* \end{aligned}$$

and that $\dot{\mu }(t)=\text {Im}\langle \varphi _t,\dot{\varphi }_t\rangle +\Vert \omega ^{1/2}\varphi _t\Vert _{L^2}^2+e(\varphi _t)$. With this we compute the generator ${\mathscr {L}}_{{\mathscr {D}},K}^{\varepsilon }(t)$ of the dressed dynamics defined by $i\partial _t\xi _K(t)={\mathscr {L}}_{{\mathscr {D}},K}^{\varepsilon }(t)\,\xi _K(t)$.

A straightforward computation, using $i\dot{\varphi }_t=\omega \varphi _t+\sigma (\psi _{\varphi _t})$, yields

7.16 $$\begin{aligned} {\mathscr {L}}_{{\mathscr {D}},K}^{\varepsilon }(t)&=e^{-ig_{K,\varphi _t}(x)}\,W(\varepsilon ^{-1}\varphi _t)^*\,\varepsilon ^{-2}H^{\varepsilon }_{{\mathscr {D}},K}\,W(\varepsilon ^{-1}\varphi _t)\,e^{ig_{K,\varphi _t}(x)}\nonumber \\&\quad -\varepsilon ^{-2}\big (\Vert \omega ^{1/2}\varphi _t\Vert _{L^2}^2-e(\varphi _t)\big )-\varepsilon ^{-1}\phi (\omega \varphi _t+\sigma (\psi _{\varphi _t})) + 2\text {Re}\langle {{\textbf {B}}}_K,\omega \varphi _t+\sigma (\psi _{\varphi _t})\rangle \nonumber \\&=\varepsilon ^{-2}\big (h_{\varphi _t}-e(\varphi _t)\big ) + \varepsilon ^{-1}\big (A_K-\phi (\sigma (\psi _{\varphi _t}))\big )\nonumber \\&\quad +\big (H_f+D_K+2\text {Re}\langle {{\textbf {B}}}_K,\sigma (\psi _{\varphi _t})\rangle \big ) - \varepsilon \,\phi (\omega {{\textbf {B}}}_K)+\varepsilon ^2\Vert \omega ^{1/2}B_K\Vert _{L^2}^2, \end{aligned}$$

where we used (4.7) and (4.12) for $\varphi = \varphi _t$ and, for convenience, recall that

7.17a $$\begin{aligned} A_K&=\phi ({{\textbf {G}}}_K)+2\,a^*(k{{\textbf {B}}}_K)\cdot p+2\,p\cdot a(k{{\textbf {B}}}_K), \end{aligned}$$

7.17b $$\begin{aligned} D_K&=2\,a^*(k{{\textbf {B}}}_K)\,a(k{{\textbf {B}}}_K)+a^*(k{{\textbf {B}}}_K)^2+a(k{{\textbf {B}}}_K)^2+4\pi \ln K, \end{aligned}$$

Next we expand $\delta (t) = \xi _K(t)-\zeta (t)$, where we abbreviate $\zeta (t)=\psi _{\varphi _t}\otimes {\mathbb {U}}(t)\Omega$. With

7.18 $$\begin{aligned} \partial _t \xi _K(t) = -i {\mathscr {L}}_{{\mathscr {D}},K}^\varepsilon (t) \xi _K(t), \qquad \partial _t\zeta (t)= \big (-i {\mathbb {H}}_{{\mathscr {D}},K,\infty }(t)+ R_{\varphi _t}V_{i\omega \varphi _t}\big )\zeta (t) \end{aligned}$$

and $\Vert \xi _K(t) \Vert = \Vert \zeta (t) \Vert = 1$, we compute

7.19 $$\begin{aligned} \frac{1}{2} \frac{d}{dt} \Vert \delta (t)\Vert ^2&= - \frac{d}{dt} \text {Re}\langle \xi _K(s) , \zeta (s) \rangle \nonumber \\&= \text {Im}\Big \langle \xi _K(s),\big ({\mathscr {L}}_{{\mathscr {D}},K}^\varepsilon (s)-{\mathbb {H}}_{{\mathscr {D}},K,\infty }(s)-i\,R_{\varphi _s}V_{i\omega \varphi _s}\big )\zeta (s)\Big \rangle , \end{aligned}$$

where we used that ${\mathscr {L}}_{{\mathscr {D}},K}^\varepsilon (s)$ is symmetric. Since δ(0)=0, integration from zero to *t* gives

7.20 $$\begin{aligned} \Vert \delta (t) \Vert ^2&=2\text {Im}\int _0^t \text {d}s\,\Big \langle \xi _K(s),\big ({\mathscr {L}}_{{\mathscr {D}},K}^\varepsilon (s)-{\mathbb {H}}_{{\mathscr {D}},K,\infty }(s)-i\,R_{\varphi _s}V_{i\omega \varphi _s}\big )\zeta (s)\Big \rangle \nonumber \\&=2\text {Im}\Big ({\mathscr {M}}^{(1)}+{\mathscr {M}}^{(2)}+{\mathscr {R}}^{(3)}+{\mathscr {R}}^{(4)}+{\mathscr {R}}^{(5)}\Big ). \end{aligned}$$

Here, we split into two main terms and three remainders:

7.21a $$\begin{aligned} {\mathscr {M}}^{(1)}&=\int _0^t \text {d}s\,\Big \langle \xi _K(s),\,\varepsilon ^{-1}\big (A_K-\phi (\sigma _{\psi _{\varphi _s}})\big )\,\zeta (s)\Big \rangle ,\end{aligned}$$

7.21b $$\begin{aligned} {\mathscr {M}}^{(2)}&=\int _0^t \text {d}s\,\Big \langle \xi _K(s),\,P_{\varphi _s}A_KR_{\varphi _s}A_K\,\zeta (s)\Big \rangle ,\end{aligned}$$

7.21c $$\begin{aligned} {\mathscr {R}}^{(3)}&=\int _0^t \text {d}s\,\Big \langle \xi _K(s),\,Q_{\varphi _s}\big (D_K+2\text {Re}\langle {{\textbf {B}}}_K,\sigma (\psi _{\varphi _s})\rangle \big )\,\zeta (s)\Big \rangle ,\end{aligned}$$

7.21d $$\begin{aligned} {\mathscr {R}}^{(4)}&=\int _0^t \text {d}s\,\Big \langle \xi _K(s),\,\big (-i\,R_{\varphi _s}V_{i\omega \varphi _s}\big )\,\zeta (s)\Big \rangle ,\end{aligned}$$

7.21e $$\begin{aligned} {\mathscr {R}}^{(5)}&=\int _0^t \text {d}s\,\Big \langle \xi _K(s),\,\big (-\varepsilon \,\phi (\omega {{\textbf {B}}}_K)+\varepsilon ^2\Vert \omega ^{1/2}B_K\Vert _{L^2}^2\big )\,\zeta (s)\Big \rangle . \end{aligned}$$

We recall the definition of the projections $P_{\varphi _s}=|\psi _{\varphi _s}\rangle \langle \psi _{\varphi _s}|$ and $Q_{\varphi _s}={{\textbf {1}}}-P_{\varphi _s}$. We also use repeatedly the identity

7.22 $$\begin{aligned} {\mathbb {H}}_{{\mathscr {D}},K,\infty }(s)\,\zeta (s) = P_{\varphi _s}\big (H_f+D_K+2\text {Re}\langle {{\textbf {B}}}_K,\sigma (\psi _{\varphi _s})\rangle - A_KR_{\varphi _s}A_K\big )\,\zeta (s). \end{aligned}$$

Before proceeding with the expansion, we bound the error terms from the third and fifth lines.

**Line **(7.21c). Write ${\mathscr {R}}^{(3)}={\mathscr {R}}^{(3.1)}+{\mathscr {R}}^{(3.2)}$. For the second term, we estimate

7.23 $$\begin{aligned} |{\mathscr {R}}^{(3.2)} |&\lesssim \int _0^t \text {d}s \Big | \Big \langle \xi _K (s) , Q_{\varphi _s} \text {Re}\langle {{\textbf {B}}}_K, \sigma ( \psi _{\varphi _s} ) \rangle \zeta (s) \Big \rangle \Big | \lesssim \Vert B_K \Vert _{L^2} \int _0^t \text {d}s \Vert \sigma ( \psi _{\varphi _s} ) \Vert _{L^2} \lesssim K^{-1} \end{aligned}$$

where we used $\Vert B_K \Vert _{L^2} \lesssim K^{-1}$ and $\Vert \sigma ( \psi _{\varphi _s} ) \Vert _{L^2} \lesssim 1$ by Lemma 3.8. For the first term, use $Q_{\varphi _s}D_K\zeta (s)=Q_{\varphi _s}(D_K-4\pi \ln K)\zeta (s)$, since $Q_{\varphi _s} \zeta (s) =0$, and then

7.24 $$\begin{aligned} |{\mathscr {R}}^{(3.1)}|&\leqslant \int _0^t \text {d}s\,\big |\langle \xi _K(s),\,Q_{\varphi _s}(D_K-4\pi \ln K)\,\zeta (s)\rangle \big |\nonumber \\&\lesssim \int _0^t \text {d}s\,\Vert \langle p\rangle \,\xi _K(s)\Vert \,\Vert \langle p\rangle ^{-1}(D_K-4\pi \ln K) \langle p \rangle ^{-1} \langle N^\frac{1}{2} \rangle ^{-2} \Vert \, \Vert \langle p \rangle \langle N^\frac{1}{2}\rangle ^2 \zeta (s)\Vert \nonumber \\&\lesssim K^{-1/4}, \end{aligned}$$

where we applied Lemma 7.1, (3.38d) and Lemma 6.4. Note that the latter implies

7.25 $$\begin{aligned} \Vert \langle p\rangle \langle N^{\frac{1}{2}}\rangle ^{2}\zeta (s)\Vert \lesssim \Vert \psi _{\varphi _s}\Vert _{H^1}\,\Vert \langle N^{\frac{1}{2}}\rangle ^{2} {\mathbb {U}}(s)\Omega \Vert \lesssim 1. \end{aligned}$$

Adding both bounds, we get

7.26 $$\begin{aligned} | {\mathscr {R}}^{(3)} | \lesssim K^{-1/4}. \end{aligned}$$

**Line **(7.21e). Write ${\mathscr {R}}^{(5)}={\mathscr {R}}^{(5.1)}+{\mathscr {R}}^{(5.2)}$. For the term proportional to ε, we estimate

7.27 $$\begin{aligned} | {\mathscr {R}}^{(5.1)} |&\lesssim \varepsilon \int _0^t \text {d}s\, \Vert \langle p \rangle \xi _K(s) \Vert \, \langle p \rangle ^{-1} \phi (\omega {{\textbf {B}}}_K) \langle N^\frac{1}{2} \rangle ^{-1} \langle p\rangle ^{-1} \Vert \, \Vert \langle p \rangle \langle N^\frac{1}{2}\rangle \zeta (s) \Vert \nonumber \\&\lesssim \varepsilon K^{-3/4}, \end{aligned}$$

where we applied (3.24c), (7.25) and Lemma 7.1. Using $\Vert \omega ^{1/2}B_K\Vert _{L^2}^2\leqslant C K^{-1}$, the term proportional to $\varepsilon ^2$ satisfies $|{\mathscr {R}}^{(5.2)}|\lesssim \varepsilon ^2 K^{-1}$.

Since the remaining terms in (7.20) still contain contributions of order $O(\varepsilon ^{-1})$ and *O*(1), the first-order expansion is not sufficient to obtain the desired result. In the next two sections, we use an adiabatic expansion scheme to expand these terms to higher orders in ε.

### Second-order expansion

The key ingredient for the higher-order expansion is the following identity

7.28 $$\begin{aligned} Q_{\varphi _s} \xi _K (s)&= R_{\varphi _s} \Big ( h_{\varphi _s} - e ( \varphi _s) \Big ) \xi _K (s) \nonumber \\&= R_{\varphi _s} \Big ( \varepsilon ^2 \left( i\partial _s - {\mathscr {L}}_{{\mathscr {D}}, K}^\varepsilon (s) \right) + h_{\varphi _s} - e ( \varphi _s) \Big ) \xi _K (s) \nonumber \\&= R_{\varphi _s} \Big ( - \varepsilon \big ( A_K - \phi ( \sigma _{\psi _{\varphi _s}} ) \big ) + \varepsilon ^2 \big ( i\partial _s - H_f - D_K - 2\text {Re}\langle {{\textbf {B}}}_K , \sigma ( \psi _{\varphi _s}) \rangle \big ) \nonumber \\&\hspace{5.5cm} + \varepsilon ^3 \phi (\omega {{\textbf {B}}}_K) - \varepsilon ^4 \Vert \omega ^{1/2} B_K\Vert ^2_{L^2} \Big ) \xi _K (s) , \end{aligned}$$

where we used $(i\partial _s - {\mathscr {L}}_{{\mathscr {D}},K}^{\varepsilon } (s) ) \xi _K (s) =0$ and (7.16).

We now systematically replace $Q_{\varphi _s} \xi _K(s)$ via (7.28). The basic idea is that on $\text {Ran}(Q_{\varphi _s})\subset L^2\otimes {\mathcal {F}}$, due to the uniform spectral gap of $R_{\varphi _s}$, one can treat the operator $\varepsilon ^2 ( i\partial _s - {\mathscr {L}}_{{\mathscr {D}}, K}^{\varepsilon } (s) ) + ( h_{\varphi _s} - e(\varphi _s))$ as a perturbation of order O(ε).

To use the above identity in ${\mathscr {M}}^{(1)}$, it is crucial to observe that

7.29 $$\begin{aligned} {\mathscr {M}}^{(1)}&= \int _0^t \text {d}s\,\Big \langle \xi _K(s),\,\varepsilon ^{-1}\big (A_K-\phi (\sigma _{\psi _{\varphi _s}})\big )\,\zeta (s)\Big \rangle = \int _0^t \text {d}s \Big \langle \xi _K(s) , \varepsilon ^{-1} Q_{\varphi _s} A_K \zeta (s) \Big \rangle \end{aligned}$$

which follows from ${\textbf{1}}=P_{\varphi _s} + Q_{\varphi _s}$, $Q_{\varphi _s} \phi ( \sigma ( \psi _{\varphi _s} ) ) \zeta (s) = \phi ( \sigma ( \psi _{\varphi _s} ) ) Q_{\varphi _s} \zeta (s) = 0$ and the identity

7.30 $$\begin{aligned} P_{\varphi _s} ( A_K - \phi (\sigma ( \psi _{\varphi _s} ) ) ) P_{\varphi _s} =0. \end{aligned}$$

To verify the latter, let us compute

7.31 $$\begin{aligned} P_{\varphi _s} ( a^*( G_K ) + 2 a^*( k {{\textbf {B}}}_K ) \cdot p ) P_{\varphi _s}&= P_{\varphi _s} \otimes \int \text {d}k\, \langle \psi _{\varphi _s}, ( {{\textbf {G}}}_K (\cdot , k) +2 k {{\textbf {B}}}_K(\cdot ,k) \cdot p) \psi _{\varphi _s} \rangle \, a_k^* \nonumber \\&= P_{\varphi _s} \otimes a^*(\sigma ( \psi _{\varphi _s}) ), \end{aligned}$$

where we used $\langle \psi _{\varphi _s}, {{\textbf {G}}}_K \psi _{\varphi _s} \rangle = \sigma ( \psi _{\varphi _s} ) {\textbf{1}}_{|k|\leqslant K}$ and $2 \langle \psi _{\varphi _s}, ( k{{\textbf {B}}}_K \cdot p ) \psi _{\varphi _s} \rangle = \langle \psi _{\varphi _s}, G \psi _{\varphi _s} \rangle {\textbf{1}}_{|k|\geqslant K} = \sigma ( \psi _{\varphi _s} ) {\textbf{1}}_{|k|\geqslant K}$ as a consequence of $\psi _{\varphi _s}$ being real-valued and $(p\cdot \overline{k {{\textbf {B}}}_K}) = {{\textbf {G}}} {\textbf{1}}_{|k|\geqslant K}$ with ${{\textbf {G}}}$ defined in (1.7).

Because of the presence of the $P_{\varphi _s}$ projection, (7.28) is not applicable in ${\mathscr {M}}^{(2)}$. This term is genuinely of order one on the macroscopic time scale. One of the goals of the expansion is therefore to extract from ${\mathscr {M}}^{(1)}$ a contribution that cancels ${\mathscr {M}}^{(2)}$.

In the remainder of this section, we apply (7.28) to the contributions ${\mathscr {M}}^{(1)}$ and ${\mathscr {R}}^{(4)}$ in (7.20). Each step replaces $Q_{\varphi _s}\xi _K(s)$ by the right side of (7.28) and uses integration by parts to remove the time-derivative from $\xi _K(s)$. The additional terms generated by this will be shown to be of higher order in ε (except for the one that will cancel ${\mathscr {M}}^{(2)}$).

With (7.29), we can apply (7.28) to the first term ${\mathscr {M}}^{(1)}$. We split the resulting terms into ${\mathscr {M}}^{(1)} = {\mathscr {M}}^{(1.1)} +{\mathscr {R}}^{(1.2)} + {\mathscr {R}}^{(1.3)} + {\mathscr {R}}^{(1.4)}$ with

7.32a $$\begin{aligned} {\mathscr {M}}^{(1.1)}&= - \int _0^t \text {d}s \Big \langle \xi _K(s) , \big ( A_K - \phi (\sigma ( \psi _{\varphi _s} ) ) \big ) R_{\varphi _s} A_K \zeta (s) \Big \rangle , \end{aligned}$$

7.32b $$\begin{aligned} {\mathscr {R}}^{(1.2)}&= \varepsilon \int _0^t \text {d}s \Big \langle \Big ( i\partial _s - H_f \Big ) \xi _K (s) , R_{\varphi _s} A_K \zeta (s) \Big \rangle , \end{aligned}$$

7.32c $$\begin{aligned} {\mathscr {R}}^{(1.3)}&= - \varepsilon \int _0^t \text {d}s \Big \langle \xi _K (s) , D_K R_{\varphi _s} A_K \zeta (s) \Big \rangle \end{aligned}$$

7.32d $$\begin{aligned} {\mathscr {R}}^{(1.4)}&= \int _0^t \text {d}s \Big \langle \xi _K (s) , \Big ( - 2 \varepsilon \text {Re}\langle {{\textbf {B}}}_K, \sigma ( \psi _{\varphi _s} ) \rangle \!+\! \varepsilon ^2 \phi (\omega {{\textbf {B}}}_K ) \!-\! \varepsilon ^3 \Vert \omega ^{\frac{1}{2} } B_K \Vert ^2_{L^2} \Big ) R_{\varphi _s} A_K \zeta (s) \Big \rangle . \end{aligned}$$

To extract the term of order *O*(1), we repeat the same steps for ${\mathscr {M}}^{(1.1)}$. Inserting ${\textbf{1}} = P_{\varphi _s} + Q_{\varphi _s}$ and using $P_{\varphi _s} \phi ( \sigma ( \psi _{\varphi _s} ) ) R_{\varphi _s} = 0$ leads to

7.33 $$\begin{aligned} {\mathscr {M}}^{(1.1)} = - {\mathscr {M}}^{(2)} + {\mathscr {R}}^{(1.5)} \end{aligned}$$

with ${\mathscr {M}}^{(2)}$ as in (7.21c) and remainder

7.34 $$\begin{aligned} {\mathscr {R}}^{(1.5)} = -\int _0^t \text {d}s \Big \langle \xi _K (s) , Q_{\varphi _s} \big ( A_K - \phi (\sigma ( \psi _{\varphi _s} ) ) \big ) R_{\varphi _s} A_K \zeta (s) \Big \rangle . \end{aligned}$$

The important observation is the cancellation of ${\mathscr {M}}^{(2)}$. Before we proceed with this remainder, let us first estimate all other error terms.

**Line **(7.32c). After inserting identities ${\textbf{1}} = \langle p\rangle \langle p\rangle ^{-1}$ and ${\textbf{1}} = \langle N^\frac{1}{2}\rangle ^{-1} \langle N^\frac{1}{2}\rangle ^{1}$, using $[N,R_{\varphi _s}] =0$, and applying the Cauchy–Schwarz inequality, we obtain

7.35 $$\begin{aligned} | {\mathscr {R}}^{(1.3)}|&\leqslant \varepsilon \int ^t_0 \text {d}s \Big ( \Vert \langle p\rangle \xi _K (s) \Vert \, | \langle p\rangle ^{-1} D_K \langle p\rangle ^{-1}\langle N^\frac{1}{2} \rangle ^{-2}\Vert \, \Vert \langle p\rangle R_{\varphi _s} \langle p \rangle \Vert \nonumber \\&\qquad \qquad \times \, \Vert \langle N^\frac{1}{2} \rangle ^{2} \langle p\rangle ^{-1} A_K \langle p\rangle ^{-1} \langle N^\frac{1}{2}\rangle ^{-3} \Vert \, \Vert \langle N^\frac{1}{2} \rangle ^3 \langle p\rangle \zeta (s) \Vert \Big ) \, . \end{aligned}$$

Next we apply $\Vert \langle p \rangle \xi _K(s) \Vert \lesssim K^\frac{1}{2}$, see Lemma 7.1, and $\Vert \langle N^\frac{1}{2} \rangle ^3 \langle p\rangle \zeta (s) \Vert \lesssim 1$, see Lemma 6.4. The bound $\Vert \langle p\rangle R_{\varphi _s} \langle p \rangle \Vert \lesssim 1$ follows from Lemma 5.3. The remaining two operator norms are estimated in Lemma 3.6. Putting everything together yields the bound

7.36 $$\begin{aligned} | {\mathscr {R}}^{(1.3)}|&\lesssim \varepsilon K^\frac{1}{2} ( \ln K)^\frac{3}{2} . \end{aligned}$$

**Line **(7.32d). Using in addition (3.24c) and $\Vert \langle p \rangle R_{\varphi _s} \langle p \rangle \Vert \lesssim 1$, the fourth term is estimated similarly as

7.37 $$\begin{aligned} |{\mathscr {R}}^{(1.4)}| \lesssim (\ln K)^\frac{1}{2} \Big ( \varepsilon K^{-1} + \varepsilon ^2 K^{-\frac{1}{2} } \Big ) . \end{aligned}$$

**Line **(7.32b). Here, we need to integrate by parts. Recalling (7.18), one finds ${\mathscr {R}}^{(1.2)} = {\mathscr {R}}^{(1.21)} + {\mathscr {R}}^{(1.22)} + {\mathscr {R}}^{(1.23)} + {\mathscr {R}}^{(1.24)}$ with

7.38a $$\begin{aligned} {\mathscr {R}}^{(1.21)}&= \Big \langle i \xi _K(s) , \varepsilon R_{\varphi _s} A_K \zeta (s) \Big \rangle \Big |_{s=0}^{s=t} \end{aligned}$$

7.38b $$\begin{aligned} {\mathscr {R}}^{(1.22)}&= - \varepsilon \int _0^t \text {d}s \Big \langle \xi _K(s) , R_{\varphi _s} \Big [H_f , A_K \Big ] \zeta (s) \Big \rangle \end{aligned}$$

7.38c $$\begin{aligned} {\mathscr {R}}^{(1.23)}&= \varepsilon \int _0^t \text {d}s \Big \langle \xi _K (s) , R_{\varphi _s} A_K P_{\varphi _s} \Big ( A_K R_{\varphi _s} A_K - D_K - 2\text {Re}\langle {{\textbf {B}}}_K , \sigma ( \psi _{\varphi _s} ) \rangle \Big ) \zeta (s) \Big \rangle \end{aligned}$$

7.38d $$\begin{aligned} {\mathscr {R}}^{(1.24)}&= i \varepsilon \int _0^t \text {d}s \Big \langle \xi _K (s) , \Big ( \dot{R}_{\varphi _s} A_K - i R_{\varphi _s} A_K R_{\varphi _s} V_{i\omega \varphi _s} \Big ) \zeta (s) \Big \rangle . \end{aligned}$$

All lines except the second line are estimated using the same arguments as before. In the last line, one also invokes the bounds $\Vert \langle p\rangle \dot{R}_{\varphi _s} \langle p \rangle \Vert \lesssim 1$ (Lemma 5.3) and $\Vert \langle p\rangle ^{-1} V_{i\omega \varphi _s } \langle p \rangle ^{-1} \Vert \lesssim \Vert i\omega \varphi _s \Vert _{{\mathfrak {h}}_{-1/2}}=\Vert \varphi _s \Vert _{{\mathfrak {h}}_{1/2}} \lesssim 1$ (see (3.46c) in Lemma 3.7). The result is

7.39 $$\begin{aligned} | {\mathscr {R}}^{(1.21)}| + | {\mathscr {R}}^{(1.23)}| + | {\mathscr {R}}^{(1.24)} | \lesssim \varepsilon (\ln K)^\frac{3}{2}. \end{aligned}$$

The commutator term needs to be regularized with $\langle p^2 \rangle ^{-1}$, i.e., we estimate

7.40 $$\begin{aligned} | {\mathscr {R}}^{(1.22)} |&\leqslant \varepsilon \int _0^t \text {d}s \Vert R_{\varphi _s} \langle p^2\rangle \Vert \Vert \langle p\rangle ^{-2} [H_f , A_K ]\langle p\rangle ^{-2} \langle N^\frac{1}{2} \rangle ^{-1} \Vert \Vert \langle p^2 \rangle \langle N^\frac{1}{2}\rangle ^2 \zeta (s) \Vert \nonumber \\&\lesssim \varepsilon K \end{aligned}$$

where we used $\Vert R_{\varphi _s} \langle p^2 \rangle \Vert + \Vert \psi _{\varphi _s} \Vert _{H^2} \lesssim 1$, see Lemma 5.3, in combination with the bound (3.38c) and Lemma 6.4. Thus, we have

7.41 $$\begin{aligned} | {\mathscr {R}}^{(1.2)} | \lesssim \varepsilon K. \end{aligned}$$

**Line **(7.21d). In this term, we need to expand again via (7.28). To do this, we first introduce a cut-off L>0 by writing $\varphi _s = \varphi _{s,L}+\varphi _{s,>L}$ with $\varphi _{s,L}:= {\textbf{1}}_{|k|\leqslant L} \varphi _s$ and $V_{ i\omega \varphi _s} = V_{ i\omega \varphi _{s,L}} + V_{ i\omega \varphi _{s,>L}}$. The large-momentum part can be estimated immediately as

7.42 $$\begin{aligned}&\bigg | \int _0^t \text {d}s\,\Big \langle \xi _K(s),\,\big (-i\,R_{\varphi _s}V_{i\omega \varphi _{s,>L} }\big )\,\zeta (s)\Big \rangle \bigg | \nonumber \\&\qquad \qquad \leqslant \int _0^t \text {d}s \, \Vert R_{\varphi _s} \Vert \, \Vert V_{i\omega \varphi _{s,>L}} \langle p\rangle ^{-2} \Vert \, \Vert \langle p \rangle ^2 \zeta (s) \Vert \lesssim L^{-1/4} \end{aligned}$$

where we used (3.46b) to bound

7.43 $$\begin{aligned} \Vert V_{i\omega \varphi _{s,>L}} \langle p\rangle ^{-2} \Vert&\lesssim \Vert \omega \varphi _{s,>L} \Vert _{{\mathfrak {h}}_{-3/4}} \lesssim L^{-1/4} \Vert \varphi _s \Vert _{{\mathfrak {h}}_{1/2}} \lesssim L^{-1/4}, \end{aligned}$$

For the low-momentum part, we apply (7.28), which gives ${\mathscr {R}}^{(4)} = {\mathscr {R}}^{(4.1)} + {\mathscr {R}}^{(4.2)} + {\mathscr {R}}^{(4.3)} + {\mathscr {R}}^{(4.4)} + {\mathcal {O}}(L^{-1/4} )$ with

7.44 $$\begin{aligned} {\mathscr {R}}^{(4.1)}&= -i \varepsilon \int _0^t \text {d}s \Big \langle \xi _K(s) , \big ( - A_K + \phi (\sigma (\psi _{\varphi _s})) \big ) R_{\varphi _s}^2 V_{i \omega \varphi _{s,L}} \zeta (s) \Big \rangle \end{aligned}$$

7.45 $$\begin{aligned} {\mathscr {R}}^{(4.2)}&= -i \varepsilon ^2 \int _0^t \text {d}s \Big \langle \Big ( i\partial _s - H_f \Big ) \xi _K(s) , R_{\varphi _s}^2 V_{i \omega \varphi _{s,L}} \zeta (s) \Big \rangle \end{aligned}$$

7.46 $$\begin{aligned} {\mathscr {R}}^{(4.3)}&= i \varepsilon ^2 \int _0^t \text {d}s \Big \langle \xi _K(s) , \Big ( D_K + 2 \text {Re}\langle {{\textbf {B}}}_K, \sigma ( \psi _{\varphi _s} ) \rangle \Big ) R^2_{\varphi _s} V_{i \omega \varphi _{s,L} } \zeta (s) \Big \rangle \end{aligned}$$

7.47 $$\begin{aligned} {\mathscr {R}}^{(4.4)}&= i \int _0^t \text {d}s \Big \langle \xi _K(s) , \Big ( - \varepsilon ^3 \phi (\omega {{\textbf {B}}}_K ) + \varepsilon ^4 \Vert \omega ^{\frac{1}{2}} B_K \Vert ^2_2 \Big ) R^2_{\varphi _s} V_{i\omega \varphi _{s,L}} \zeta (s) \Big \rangle . \end{aligned}$$

All terms except the second are estimated using the same arguments as before, which yields

7.48 $$\begin{aligned} |{\mathscr {R}}^{(4.1)} | + |{\mathscr {R}}^{(4.3)} | + |{\mathscr {R}}^{(4.4)} | \lesssim \varepsilon \ln K . \end{aligned}$$

Note that the cutoff in $\varphi _{s,L}$ is not relevant for these terms, since we can estimate the potential as $\Vert \langle p \rangle ^{-1} V_{i\omega \varphi _{s,L}} \langle p\rangle ^{-1} \Vert \lesssim \Vert \varphi _s \Vert _{{\mathfrak {h}}_{1/2}} \lesssim 1$ by 3.46c.

In the second line, we proceed with integration by parts, so that ${\mathscr {R}}^{(4.2)} = {\mathscr {R}}^{(4.21)} + {\mathscr {R}}^{(4.22)} + {\mathscr {R}}^{(4.23)} + {\mathscr {R}}^{(4.24)}$ with

7.49 $$\begin{aligned} {\mathscr {R}}^{(4.21)}&= \varepsilon ^2 \Big \langle \xi _K(s) , R_{\varphi _s}^2 V_{i \omega \varphi _{s,K} } \zeta (s) \Big \rangle \Big |_{s=0}^{s=t} \nonumber \\ {\mathscr {R}}^{(4.22)}&= - i \varepsilon ^2 \int _0^t \text {d}s \Big \langle \xi _K(s) , R_{\varphi _s}^2 V_{i \omega \varphi _{s,L} } P_{\varphi _s} \Big ( A_K R_{\varphi _s} A_K - D_K - 2 \text {Re}\langle {{\textbf {B}}}_K , \sigma (\psi _{\varphi _s} ) \rangle \Big ) \zeta (s) \Big \rangle \nonumber \\ {\mathscr {R}}^{(4.23)}&= i \varepsilon ^2 \int _0^t \text {d}s \Big \langle \xi _K(s) , \Big ( 2 R_{\varphi _{s}} \dot{R}_{\varphi _{s}} V_{i \omega \varphi _{s,L}} + R_{\varphi _s}^2 \dot{V}_{i \omega \varphi _{s,L}} \Big ) \zeta (s) \Big \rangle \nonumber \\ {\mathscr {R}}^{(4.24)}&= i \varepsilon ^2 \int _0^t \text {d}s \Big \langle \xi _K(s) , \Big ( R_{\varphi _s}^2 V_{i\omega \varphi _{s,L}} R_{\varphi _s} V_{i\omega \varphi _{s} } \Big ) \zeta (s) \Big \rangle . \end{aligned}$$

All terms except for the one involving $\dot{V}_{i\omega \varphi _{s,L}}$ are estimated again as in previous terms (the cut-off *L* is not relevant for those terms), so we omit the details. The remaining term was the reason for introducing the momentum cut-off. We compute $\dot{V}_{i\omega \varphi _{s,L}} = V_{i\omega \dot{\varphi }_{s,L}} = V_{\omega ^2 \varphi _{s,L}} + V_{ \omega \sigma _L}$ with $\sigma _L:= {{\textbf {1}}} _{|k|\leqslant L} \sigma (\psi _{\varphi _s})$. Since $\omega \sigma (\psi _{\varphi _s}) = \omega ^{1/2} \widehat{ |\psi _{\varphi _s}|^2}$, we can use (3.46c) to bound

7.50 $$\begin{aligned} \Vert \langle p \rangle ^{-1} V_{\omega \sigma _L } \langle p \rangle ^{-1} \Vert&\lesssim \Vert \omega \sigma (\psi _{\varphi _s}) \Vert _{{\mathfrak {h}}_{-1/2}} = \Vert \psi _{\varphi _s}\Vert _{L^4}^2 \lesssim 1. \end{aligned}$$

Invoking also (3.46c), we find that

7.51 $$\begin{aligned} \bigg | \varepsilon ^2 \int _0^t \text {d}s \Big \langle \xi _K(s) , R_{\varphi _s}^2 \dot{V}_{i \omega \varphi _{s,L}} \zeta (s) \Big \rangle \bigg |&\lesssim \varepsilon ^2 \sup _{|s| \leqslant T} \Big ( \Vert V_{\omega ^2 \varphi _{s,L} } \langle p \rangle ^{-2} \Vert +\Vert \langle p \rangle ^{-1} V_{ \omega \sigma _L} \langle p \rangle ^{-1} \Vert \Big ) \nonumber \\&\lesssim \varepsilon ^2 \sup _{|s| \leqslant T} \Big ( \Vert \omega ^2 \varphi _{s,L} \Vert _{{\mathfrak {h}}_{-3/4}} + 1 \big ) \lesssim \varepsilon ^2 L^{3/2} . \end{aligned}$$

Adding everything up, and choosing $L=\varepsilon ^{-8/7}$ gives

7.52 $$\begin{aligned} | {\mathscr {R}}^{(4)} | \lesssim \varepsilon \ln K + \varepsilon ^{2/7}. \end{aligned}$$

**Line **(7.34). To continue with ${\mathscr {R}}^{(1.5)}$, we insert $A_K = \phi ({{\textbf {G}}}_K) + (A_K - \phi ({{\textbf {G}}}_K))$, so that ${\mathscr {R}}^{(1.5)} = {\mathscr {R}}^{(1.5a )} + {\mathscr {R}}^{(1.5b)} + {\mathscr {R}}^{(1.5c)}$ with

7.53a $$\begin{aligned} {\mathscr {R}}^{(1.5a )}&= - \int _0^t \text {d}s \Big \langle \xi _K(s) , Q_{\varphi _s} \phi ({{\textbf {G}}}_K - \sigma ( \psi _{\varphi _s}) ) R_{\varphi _s} \phi ( {{\textbf {G}}}_K) \zeta (s) \Big \rangle \end{aligned}$$

7.53b $$\begin{aligned} {\mathscr {R}}^{(1.5b )}&= \int _0^t \text {d}s \Big \langle \xi _K(s) , Q_{\varphi _s} \phi ({{\textbf {G}}}_K - \sigma ( \psi _{\varphi _s} ) ) R_{\varphi _s} (A_K-\phi ({{\textbf {G}}}_K)) \zeta (s) \Big \rangle \end{aligned}$$

7.53c $$\begin{aligned} {\mathscr {R}}^{(1.5c )}&= - \int _0^t \text {d}s \Big \langle \xi _K(s) , Q_{\varphi _s} ( A_K - \phi ({{\textbf {G}}}_K ) ) R_{\varphi _s} A_K \zeta (s) \Big \rangle . \end{aligned}$$

For ${\mathscr {R}}^{(1.5b)}$ we use (3.24a) and Lemmas 3.6, 7.1 and 6.4 to estimate

7.54 $$\begin{aligned} | {\mathscr {R}}^{(1.5b )} |&\lesssim (\ln K)^\frac{1}{2} \int _0^t \text {d}s \, \Vert \langle p \rangle \xi _K (s) \Vert \, \Vert \langle p \rangle ^{-1} \phi ({{\textbf {G}}}_K - \sigma (\psi _{\varphi _s} ) ) \langle p \rangle ^{-1} \langle N^\frac{1}{2} \rangle ^{-1} \Vert \, \Vert \langle p \rangle R_{\varphi _s} \langle p \rangle \Vert \nonumber \\&\qquad \quad \times \Vert \langle N^\frac{1}{2} \rangle \langle p \rangle ^{-1} (A_K - \phi ({{\textbf {G}}}_K) ) \langle p \rangle ^{-1} \langle N^\frac{1}{2} \rangle ^{-2} \Vert \, \Vert \langle N^\frac{1}{2}\rangle ^2 \langle p \rangle \zeta (s) \Vert \nonumber \\&\lesssim (\ln K)^\frac{1}{2} K^{-\frac{1}{4}}. \end{aligned}$$

In close analogy, one verifies that $|{\mathscr {R}}^{(1.5c)} | \lesssim (\ln K)^\frac{1}{2} K^{-\frac{1}{4}}$.

In (7.53a), there is no additional decay in *K*. Here we need to use the presence of the $Q_{\varphi _s}$ projection in order to expand one more time via (7.28).

### Third-order expansion

It remains to estimate the contribution ${\mathscr {R}}^{(1.5a)}$ defined in (7.53a). To this end, we perform a third expansion using (7.28). A direct application of (7.28) to (7.53a) would, after integration by parts, produce the term $\phi (\omega \sigma ( \psi _{\varphi _s} ))$ through the commutator $[H_f, \phi (\sigma ( \psi _{\varphi _s} )) ]$, which we cannot control in terms of the number operator. To avoid this term, we first isolate and estimate the large-momentum tail of $\sigma ( \psi _{\varphi _s} )$ and then apply the expansion only to the remaining low-momentum part. For this purpose we use the following elementary lemma.

#### Lemma 7.2

For every $L^2$-normalized $\psi \in H^2({\mathbb {R}}^3)$, and all L>1, we have

7.55 $$\begin{aligned}&\Vert \sigma ( \psi ) {\textbf{1}}_{|k|\geqslant L} \Vert _{L^2} \lesssim L^{-1} \Vert \psi \Vert _{H^2} \end{aligned}$$

7.56 $$\begin{aligned}&\Vert \omega \sigma (\psi ) {\textbf{1}}_{|k|\leqslant L} \Vert _{L^2} \lesssim (\ln L)^\frac{1}{2} \Vert \psi \Vert _{H^2}. \end{aligned}$$

#### Proof

Writing $e^{-ikx} = |k|^{-4} [ (k\cdot p), [ ( k\cdot p),e^{ - i kx} ] ]$, we can estimate

7.57 $$\begin{aligned} \Vert \sigma (\psi ) {\textbf{1}}_{|k|\geqslant L}\Vert _{L^2}^2&= \int \limits _{|k|\geqslant L} \text {d}k \, \omega ^{-1}(k) |\langle \psi , e^{-ik(\cdot )} \psi \rangle |^2 \lesssim \Vert \psi \Vert _{H^2} \int \limits _{|k|\geqslant L} \frac{\text {d}k}{|k|^{5}} . \end{aligned}$$

For the second one, use $\Vert \omega \sigma ( \psi ) {\textbf{1}}_{|k|\leqslant L} \Vert _{L^2} \lesssim 1 + \Vert \omega \sigma ( \psi ) {\textbf{1}}_{2\leqslant |k|\leqslant K} \Vert _{L^2}$ and estimate again with the above identity,

7.58 $$\begin{aligned} \Vert \omega \sigma ( \psi ) {\textbf{1}}_{2\leqslant |k|\leqslant L} \Vert _{L^2}^2&= \int \limits _{2\leqslant |k|\leqslant K} \text {d}k\, \omega (k) |\langle \psi , e^{-ik(\cdot )} \psi \rangle |^2 \lesssim \Vert \psi \Vert _{H^2} \int \limits _{2\leqslant |k|\leqslant L} \frac{\text {d}k}{|k|^{3}} . \end{aligned}$$

This proves the lemma. □

Coming back to the estimate for (7.53a), let us abbreviate $\sigma _K:= \sigma ( \psi _{\varphi _s}) {{\textbf {1}}}_{|k|\leqslant K}$ and $\sigma _{K}:= \sigma ( \psi _{\varphi _s}) {{\textbf {1}}}_{|k|> K}$. We split ${\mathscr {R}}^{(1.5a )} = {\mathscr {R}}^{(1.5a1)} + {\mathscr {R}}^{(1.5a2)}$ into

7.59 $$\begin{aligned} {\mathscr {R}}^{(1.5a1 )}&= - \int _0^t \text {d}s \Big \langle \xi _K(s) , Q_{\varphi _s} \phi ({{\textbf {G}}}_K - \sigma _K ) R_{\varphi _s} \phi ( {{\textbf {G}}}_K) \zeta (s) \Big \rangle , \end{aligned}$$

7.60 $$\begin{aligned} {\mathscr {R}}^{(1.5a2 )}&= \int _0^t \text {d}s \Big \langle \xi _K(s) , Q_{\varphi _s} \phi ( \sigma _{> K} ) R_{\varphi _s} \phi ( {{\textbf {G}}}_K) \zeta (s) \Big \rangle \end{aligned}$$

The large-momentum part is bounded by

7.61 $$\begin{aligned} | {\mathscr {R}}^{(1.5a2 )} |&\lesssim \int _0^t \text {d}s \Big ( \Vert \sigma _{> K} \Vert _{L^2} \Vert R_{\varphi _s} \langle p \rangle \Vert \nonumber \\&\qquad \times \Vert \langle p \rangle ^{-1} \phi ({{\textbf {G}}}_K) \langle p \rangle ^{-1} \langle N^\frac{1}{2} \rangle ^{-1} \Vert \, \Vert \langle p \rangle \langle N^\frac{1}{2} \rangle \zeta (s) \Vert \Big ) \nonumber \\&\lesssim K^{-1} (\ln K)^{1/2} \end{aligned}$$

where we applied (3.24a) together with Lemmas 5.3, 6.4 and 7.2.

**Line **(7.59). For shorter notation, let us further abbreviate $L_K:= \phi ({{\textbf {G}}}_K - \sigma _K ) R_{\varphi _s} \phi ({{\textbf {G}}}_K)$. Applying identity (7.28), we obtain ${\mathscr {R}}^{(1.5a1)} = {\mathscr {R}}^{(6.1)} + {\mathscr {R}}^{(6.2)} + {\mathscr {R}}^{(6.3)} + {\mathscr {R}}^{(6.4)}$ with

7.62 $$\begin{aligned} {\mathscr {R}}^{(6.1)}&= \int _0^t \text {d}s \Big \langle \xi _K(s) , \varepsilon \big ( A_K - \phi (\sigma _{\psi _s} ) \big ) R_{\varphi _s} L_K \zeta (s) \Big \rangle , \end{aligned}$$

7.63 $$\begin{aligned} {\mathscr {R}}^{(6.2)}&= -\varepsilon ^2 \int _0^t \text {d}s \Big \langle \Big ( i\partial _s - H_f \Big ) \xi _K (s) , R_{\varphi _s} L_K \zeta (s) \Big \rangle , \end{aligned}$$

7.64 $$\begin{aligned} {\mathscr {R}}^{(6.3)}&= \int _0^t \text {d}s \Big \langle \xi _K(s) , \varepsilon ^2 \Big ( D_K + 2 \varepsilon ^2 \text {Re}\langle {{\textbf {B}}}_K , \sigma ( \psi _{\varphi _s}) \rangle \Big ) R_{\varphi _s} L_K \zeta (s) \Big \rangle , \end{aligned}$$

7.65 $$\begin{aligned} {\mathscr {R}}^{(6.4)}&= \int _0^t \text {d}s \Big \langle \xi _K (s) , \Big ( - \varepsilon ^3 \phi (\omega {{\textbf {B}}}_K) + \varepsilon ^4 \Vert \omega ^{1/2} B_K \Vert ^2_{L^2} \Big ) R_{\varphi _s} L_K \zeta (s) \Big \rangle . \end{aligned}$$

All terms except the second line can be estimated in exact analogy to previous terms. The resulting estimate is

7.66 $$\begin{aligned} |{\mathscr {R}}^{(6.1)} | + | {\mathscr {R}}^{(6.3)} | + | {\mathscr {R}}^{(6.4)} | \lesssim \varepsilon (\ln K)^{\frac{3}{2}} + \varepsilon ^2 (\ln K)^2 \end{aligned}$$

In (7.63), we integrate by parts to find ${\mathscr {R}}^{(6.2)} = {\mathscr {R}}^{(6.21)} + {\mathscr {R}}^{(6.22)} + {\mathscr {R}}^{(6.23)} + {\mathscr {R}}^{(6.24)}$ with

7.67 $$\begin{aligned} {\mathscr {R}}^{(6.21)}&= - i \varepsilon ^2 \Big \langle \xi _K (s) , R_{\varphi _s} L_K \zeta (s) \Big \rangle \Big |_0^t \end{aligned}$$

7.68 $$\begin{aligned} {\mathscr {R}}^{(6.22)}&= - \varepsilon ^2 \int _0^t \text {d}s \Big \langle \xi _K (s) , R_{\varphi _s} \Big [ H_f , L_K \Big ] \zeta (s) \Big \rangle \end{aligned}$$

7.69 $$\begin{aligned} {\mathscr {R}}^{(6.23)}&= \varepsilon ^2 \int _0^t \text {d}s \Big \langle \xi _K (s) , R_{\varphi _s} L_K P_{\varphi _s} \Big ( A_K R_{\varphi _s} A_K - D_K - 2\text {Re}\langle {{\textbf {B}}}_K , \sigma ( \psi _{\varphi _s} ) \rangle \Big ) \zeta (s) \Big \rangle , \end{aligned}$$

7.70 $$\begin{aligned} {\mathscr {R}}^{(6.24)}&= -i \varepsilon ^2 \int _0^t \text {d}s \Big \langle \xi _K (s) , \Big ( \dot{R}_{\varphi _s} L_K + R_{\varphi _s} \dot{L}_K \big ) \zeta (s) \Big \rangle . \end{aligned}$$

These terms can be bounded again in the same way as previous error terms. In particular, for the commutator, we have

7.71 $$\begin{aligned} [H_f , L_K ]&= \phi ^-(\omega {{\textbf {G}}}_K - \omega \sigma _K ) R_{\varphi _s} \phi ({{\textbf {G}}}_K) + \phi ({{\textbf {G}}}_K - \sigma _ K ) R_{\varphi _s} \phi ^-(\omega {{\textbf {G}}}_K) \end{aligned}$$

where we recall that $\phi ^-({{\textbf {F}}}) = a^*({{\textbf {F}}}) - a({{\textbf {F}}})$. Using (3.24a), (3.24b) and Lemmas 3.8, 5.3 and 7.2, one readily verifies for $m\in {\mathbb {N}}_0$

7.72 $$\begin{aligned}&\Vert \langle p \rangle ^{-1} \langle N^\frac{1}{2}\rangle ^{m} \phi ^-( {{\textbf {G}}}_K - \sigma _K ) \langle N^\frac{1}{2}\rangle ^{-m-1} \langle p \rangle ^{-1} \Vert \lesssim (\ln K)^\frac{1}{2} + 1 , \end{aligned}$$

7.73 $$\begin{aligned}&\Vert \langle p \rangle ^{-1} \langle N^\frac{1}{2}\rangle ^{m} \phi ^-(\omega {{\textbf {G}}}_K - \omega \sigma _K ) \langle N^\frac{1}{2}\rangle ^{-m-1} \langle p \rangle ^{-1} \Vert \lesssim K + \ln K \, . \end{aligned}$$

In total, we arrive at

7.74 $$\begin{aligned} | {\mathscr {R}}^{(6)} | \leqslant \varepsilon ^2 K \ln K . \end{aligned}$$

Combining this with (7.61) and (7.66), as well as with the estimates for ${\mathscr {R}}^{(1.5b)}$ and ${\mathscr {R}}^{(1.5c)}$, we arrive at

7.75 $$\begin{aligned} |{\mathscr {R}}^{(1.5)} | \lesssim \varepsilon ^2 K \ln K + (\ln K)^\frac{1}{2} K^{-1/4}. \end{aligned}$$

### Conclusion of the proof

We now gather all estimates, starting from the expansion of δ(t) in (7.20). Recalling the cancellation in (7.33) and combining the error bounds (7.26), (7.36), (7.37), (7.41), (7.52), and (7.75), we obtain

7.76 $$\begin{aligned} \Vert \delta (t) \Vert ^2 \;\lesssim \; (\ln K)^{1/2} K^{-1/4} + \varepsilon K + \varepsilon ^2 K \ln K + \varepsilon ^{2/7}. \end{aligned}$$

Optimizing by choosing $K = \varepsilon ^{-4/5}$ proves Theorem 2.1.

One could in principle expand all terms containing a $Q_{\varphi _s}$ projection even further, which would lead to a sharper error bound. For the sake of clarity and conciseness, we do not pursue this refinement here.

## Proof of convergence of reduced densities

We start with the proof of Corollary 2.2, which follows directly from Theorem 2.1.

### Proof of Corollary 2.2

Let us abbreviate $\Psi _t^\varepsilon = e^{- i t\varepsilon ^{-2} H^\varepsilon + i \varepsilon ^{-2} \mu (t) } \Psi _0$ with initial state $\Psi _0 = \psi _{\varphi _0} \otimes W(\varepsilon ^{-1} \varphi _0 ) \Omega$, and $\Phi _t = \psi _{\varphi _t} \otimes W(\varepsilon ^{-1} \varphi _t ) {\mathbb {U}}(t) \Omega$, and observe that $\gamma _{\textrm{particle}}^\varepsilon (t) = \text {Tr}_{{\mathcal {F}}} | \Psi _t^\varepsilon \rangle \langle \Psi _t^\varepsilon |$ and $|\psi _{\varphi _t} \rangle \langle \psi _{\varphi _t} | = \text {Tr}_{{\mathcal {F}}} | \Phi _t \rangle \langle \Phi _t |$. We then write

8.1 $$\begin{aligned} \gamma _{\textrm{particle}}^\varepsilon (t) - |\psi _{\varphi _t} \rangle \langle \psi _{\varphi _t} |&= \text {Tr}_{{\mathcal {F}}} | \Psi _t^\varepsilon \rangle \langle \Psi _t^\varepsilon | - \text {Tr}_{{\mathcal {F}}} | \Phi _t \rangle \langle \Phi _t | \nonumber \\&\quad = \text {Tr}_{{\mathcal {F}}} | \Psi _t^\varepsilon \rangle \langle \Psi _t^\varepsilon - \Phi _t | + \text {Tr}_{{\mathcal {F}}} | \Psi _t^\varepsilon - \Phi _t \rangle \langle \Phi _t | \end{aligned}$$

to bound $\text {Tr}_{L^2} | \gamma _{\textrm{particle}}^\varepsilon (t) - |\psi _t \rangle \langle \psi _t | | \leqslant 2 \Vert \Psi _t^\varepsilon - \Phi _t \Vert \leqslant C \varepsilon ^{1/10} \ln \tfrac{1}{\varepsilon }$, by Theorem 2.1. □

The proof of Corollary 2.3 requires some additional work. The main difficulty lies in controlling the unbounded operators appearing in (2.14) and (2.15). To prepare for this, we establish two auxiliary lemmas. The first provides an improved estimate on the propagated field energy, while the second yields an a priori bound on $\Vert N_{\leqslant L}\,\xi _K(t)\Vert$, where the cut-off number operator is given by

$$ N_{\leqslant L} \;=\;\int _{|k|\leqslant L} \, a_k^* a_k \, \text {d}k. $$

### Lemma 8.1

Let $\xi _K(t)$ be defined in (7.5). There exist constants C,T>0, $K_0\geqslant 2$ such that

8.2 $$\begin{aligned} \big \Vert H_f^{1/2} \xi _K(t) \big \Vert ^2 \;\leqslant \; C \,\varepsilon ^{-2} \ln K \end{aligned}$$

for all |t|⩽T, ε>0 and $K \geqslant K_0$.

This estimate improves the field energy bound from Lemma 7.1.

### Proof of Lemma 8.1

As in the initial steps of the proof of Lemma 7.1, we start from

8.3 $$\begin{aligned} \big \Vert \varepsilon H_f^{1/2} \xi _K(t) \big \Vert&\leqslant \Big \Vert \big ( \varepsilon ^2 H_f + 1 \big )^{1/2} e^{-i t \varepsilon ^{-2} H^{ \varepsilon }_{{\mathscr {D}},K} } W(\varepsilon ^{-1} \varphi _0 ) e^{ i g_{K,\varphi _0} (x)} \psi _{\varphi _0} \otimes \Omega \Big \Vert . \end{aligned}$$

By Lemma 4.1, for $L,K \geqslant K_0$ we may rewrite

8.4 $$\begin{aligned} H^{ \varepsilon }_{{\mathscr {D}},K} \;=\; U_K^\varepsilon (U_L^\varepsilon )^* \, H^\varepsilon _{{\mathscr {D}},L}\, U_L^\varepsilon (U_K^\varepsilon )^* . \end{aligned}$$

Moreover, one has the quadratic form bound

8.5 $$\begin{aligned} U_L^\varepsilon (U_K^\varepsilon )^* \, \varepsilon ^2 H_f \, U_K^\varepsilon (U_L^\varepsilon )^* \;\lesssim \; \varepsilon ^2 H_f \,+\, \varepsilon ^4\,(K^{-1/2} + L^{-1/2}) \;\lesssim \; \varepsilon ^2 H_f + 1 , \end{aligned}$$

which follows from $\Vert \omega ^{-1/2} B_K\Vert _{L^2} \lesssim K^{-1/2}$, see (3.19c). Hence,

8.6 $$\begin{aligned} \big \Vert \varepsilon H_f^{1/2} \xi _K(t) \big \Vert&\lesssim \Big \Vert \big ( \varepsilon ^2 H_f + 1 \big )^{1/2} e^{-i t \varepsilon ^{-2} H^{ \varepsilon }_{{\mathscr {D}},L } } U_L^\varepsilon (U_K^\varepsilon )^* W(\varepsilon ^{-1} \varphi _0 ) e^{ i g_{K,\varphi _0} (x)} \psi _{\varphi _0} \otimes \Omega \Big \Vert . \end{aligned}$$

Using $\varepsilon ^2 H_f \lesssim H^{ \varepsilon }_{{\mathscr {D}},L } + L$, cf. (4.3), we can remove the propagator and obtain

8.7 $$\begin{aligned} \big \Vert \varepsilon H_f^{1/2} \xi _K(t) \big \Vert&\lesssim \Big \Vert \big ( H^{ \varepsilon }_{{\mathscr {D}},L } + L \big )^{1/2} U_L^\varepsilon (U_K^\varepsilon )^* W(\varepsilon ^{-1} \varphi _0 ) e^{ i g_{K,\varphi _0} (x)} \psi _{\varphi _0} \otimes \Omega \Big \Vert . \end{aligned}$$

Next, invoking (8.4) once more and the bound

8.8 $$\begin{aligned} H^{ \varepsilon }_{{\mathscr {D}},K} \;\lesssim \; p^2 + \varepsilon ^2 H_f + \ln K \,(N+1) , \end{aligned}$$

from (4.4), we deduce

8.9 $$\begin{aligned} \big \Vert \varepsilon H_f^{1/2} \xi _K(t) \big \Vert&\lesssim \Big \Vert \big ( p^2 + \varepsilon ^{2} H_f + \varepsilon ^2 \ln K (N+1) + L \big )^{1/2} W(\varepsilon ^{-1} \varphi _0 ) e^{ i g_{K,\varphi _0} (x)} \psi _{\varphi _0} \otimes \Omega \Big \Vert . \end{aligned}$$

At this point, the argument proceeds in analogy to (7.14), yielding

8.10 $$\begin{aligned} \Vert \varepsilon H_f^{1/2} \xi _K(t) \Vert \;\lesssim \; L^{1/2} + (\ln K)^{1/2}. \end{aligned}$$

Since $L\geqslant K_0$ was arbitrary, the claim follows. □

### Lemma 8.2

Let $\xi _K(t)$ be defined by (7.5). There are C,T>0 and $K_0>2$, such that

8.11 $$\begin{aligned} \Vert N_{\leqslant L} \xi _K(t) \Vert \;\leqslant \; C\, \varepsilon ^{-2} L^2 \end{aligned}$$

for all |t|⩽T, ε>0, $K\geqslant K_0$, and $0\leqslant L \leqslant K$.

### Proof

Define

8.12 $$\begin{aligned} f(t) \;:=\; \varepsilon ^4 \Vert N_{\leqslant L}\xi _K(t)\Vert ^2 + \varepsilon ^2 L^2 \Vert N_{\leqslant L}^{1/2}\xi _K(t)\Vert ^2. \end{aligned}$$

Since $\xi _K(0) = \psi _{\varphi _0}\otimes \Omega$, we have f(0)=0. With the fundamental theorem of calculus and using $i\partial _t \xi _K(t) = {\mathscr {L}}_{{\mathscr {D}},K}^\varepsilon (t)\xi _K(t)$, we obtain $f(t) = (\textrm{I})+(\textrm{II})$ with

8.13 $$\begin{aligned} (\textrm{I})&= i \varepsilon ^4 \int _0^t \! \text {d}s \,\langle \xi _K(s), [{\mathscr {L}}_{{\mathscr {D}},K}^\varepsilon (s), N_{\leqslant L}^2] \,\xi _K(s)\rangle , \end{aligned}$$

8.14 $$\begin{aligned} (\textrm{II})&= i \varepsilon ^2 L^2 \int _0^t \! \text {d}s \,\langle \xi _K(s), [{\mathscr {L}}_{{\mathscr {D}},K}^\varepsilon (s), N_{\leqslant L}] \,\xi _K(s)\rangle . \end{aligned}$$

Next, note that all terms in ${\mathscr {L}}_{{\mathscr {D}},K}^\varepsilon (t)$ except $\varepsilon ^{-1}\phi ({{\textbf {G}}}_K)$ commute with $N_{\leqslant L}$, since $[N_{\leqslant L},H_f]=0$ and all remaining field operators are supported in $\{|k|\geqslant K\}\subset \{|k|\geqslant L\}$. Hence

8.15 $$\begin{aligned} {[}{\mathscr {L}}_{{\mathscr {D}},K}^\varepsilon (t), N_{\leqslant L}]&= \varepsilon ^{-1}\phi ^-({{\textbf {G}}}_L), \end{aligned}$$

8.16 $$\begin{aligned} {[}{\mathscr {L}}_{{\mathscr {D}},K}^\varepsilon (t), N_{\leqslant L}^2]&= \varepsilon ^{-1}\big (N_{\leqslant L}\phi ^-({{\textbf {G}}}_L)+\phi ^-({{\textbf {G}}}_L)N_{\leqslant L}\big ), \end{aligned}$$

with $$\phi ^-({{\textbf {G}}}_L) = a^*({{\textbf {G}}}_L)-a({{\textbf {G}}}_L)$$.

Using $\Vert \phi ({{\textbf {G}}}_L)\xi _K(s)\Vert \lesssim L\,\Vert (N_{\leqslant L}+1)^{1/2}\xi _K(s)\Vert$, we estimate

8.17 $$\begin{aligned} |(\textrm{I})| \;\lesssim \; \varepsilon ^3 L \int _0^t \! \text {d}s \,\Vert N_{\leqslant L}\xi _K(s)\Vert \, \Vert (N_{\leqslant L}+1)^{1/2}\xi _K(s)\Vert \;\lesssim \; \int _0^t f(s)\,\text {d}s + \varepsilon ^2. \end{aligned}$$

Similarly, since $\Vert a({{\textbf {G}}}_L)\xi _K(s) \Vert \lesssim L\,\Vert N_{\leqslant L}^{1/2}\xi _K(s) \Vert$, we find

8.18 $$\begin{aligned} |(\textrm{II})| \;\lesssim \; \varepsilon L^3 \int _0^t \! \text {d}s \,\Vert N_{\leqslant L}^{1/2}\xi _K(s)\Vert \;\leqslant \; \int _0^t f(s)\,\text {d}s + L^4. \end{aligned}$$

Collecting terms, we obtain

8.19 $$\begin{aligned} f(t) \;\lesssim \; \int _0^t f(s)\,\text {d}s + L^4. \end{aligned}$$

By Grönwall’s inequality and f(0)=0, this yields $f(t)\lesssim L^4$, as claimed. □

We now prove Corollary 2.3, as a consequence of the preceding lemmas together with the bound established in (7.76).

### Proof of Corollary 2.3

As the implication from (2.14) to (2.15) is well-known and follows by standard arguments, we omit the proof and concentrate on (2.14).

We abbreviate

8.20 $$\begin{aligned} g(t) := \Big \Vert N^{1/2} W (\varepsilon ^{-1}\varphi _t)^*\,e^{-i\,\varepsilon ^{-2} t H^\varepsilon }\Psi _{K}^\varepsilon (\varphi _0) \Big \Vert ^2 , \end{aligned}$$

where $\Psi _{K}^\varepsilon (\varphi _0) = W(\varepsilon ^{-1} \varphi _0 ) (U_K^\varepsilon )^* \psi _{\varphi _0} \otimes \Omega$. Using the bound $U_K^\varepsilon N (U_K^\varepsilon )^* \;\lesssim \; N + \varepsilon ^2 \Vert B_K \Vert _{L^2}^2$, we obtain

8.21 $$\begin{aligned} g(t) \;\lesssim \; \Big \Vert N^{1/2} U_K^\varepsilon W (\varepsilon ^{-1}\varphi _t)^*\,e^{-i\,\varepsilon ^{-2} t H^\varepsilon }\Psi _{K}^\varepsilon (\varphi _0) \Big \Vert ^2 \;+\; \varepsilon ^2 K^{-1}. \end{aligned}$$

With (7.5) and (7.9) we can write

$$ U_K^\varepsilon W(\varepsilon ^{-1} \varphi _t )^* e^{-i t \varepsilon ^{-2} H^{\varepsilon }} \Psi _{K}^\varepsilon (\varphi _0) \;=\; e^{-i \varepsilon ^{-2} \mu (t)} \, \xi _K(t). $$

We now split the number operator into $N= N_{\leqslant L} + N_{L}$ with $N_{L} = \int _{|k| > L} a_k^* a_k \, \text {d}k$ for some 0<L⩽K. Since $N_{L} \leqslant L^{-1} H_f$ for L>1, Lemma 8.1 implies

8.22 $$\begin{aligned} \big \langle \xi _K(t) , N_{L} \xi _K(t) \big \rangle \;\leqslant \; L^{-1} \big \langle \xi _K(t) , H_f \xi _K(t) \big \rangle \;\leqslant \; \varepsilon ^{-2} (\ln K) L^{-1}. \end{aligned}$$

Thus

8.23 $$\begin{aligned} g(t) \;\lesssim \; \langle \xi _K( t), N_{\leqslant L} \xi _K(t) \rangle + \varepsilon ^{-2} (\ln K)\, L^{-1} + \varepsilon ^2 K^{-1}. \end{aligned}$$

To estimate the first term, recall $\zeta (t) = \psi _{\varphi _t} \otimes {\mathbb {U}}(t) \Omega$, with $\Vert N \zeta (t) \Vert \lesssim 1$ by Lemma 6.4. By Lemma 8.2 we obtain

8.24 $$\begin{aligned} \langle \xi _K (t) , N_{\leqslant L} \xi _K (t) \rangle&= \langle \xi _K (t) - \zeta (t) , N_{\leqslant L} \xi _K(t) \rangle + \langle \zeta (t) , N_{\leqslant L} \xi _K(t) \rangle \nonumber \\&\leqslant \Vert \xi _K (t) - \zeta (t) \Vert \, \Vert N_{\leqslant L} \xi _K(t) \Vert + \Vert N_{\leqslant L} \zeta (t) \Vert \nonumber \\&\lesssim \varepsilon ^{-2} L^2 \Vert \xi _K (t) - \zeta (t) \Vert + 1. \end{aligned}$$

Recalling from (7.76) that for $K=\varepsilon ^{-4/5}$

8.25 $$\begin{aligned} \Vert \delta (t)\Vert =\Vert \xi _K(t)-\zeta (t)\Vert \;\leqslant \; \varepsilon ^{1/10} \, \ln \tfrac{1}{\varepsilon } , \end{aligned}$$

we find

8.26 $$\begin{aligned} \langle \xi _K (t) , N_{\leqslant L} \xi _K (t) \rangle \;\lesssim \; \varepsilon ^{-19/10} (\ln \tfrac{1}{\varepsilon } )\,L^2 + 1 . \end{aligned}$$

Setting $K=\varepsilon ^{-4/5}$ in (8.23) and combining with the above bound this yields

8.27 $$\begin{aligned} \langle \xi _K (t) , N \xi _K(t) \rangle \;\lesssim \; \varepsilon ^{-19/10} (\ln \tfrac{1}{\varepsilon } )\, L^2 + 1 + \varepsilon ^{-2} (\ln \tfrac{1}{\varepsilon } )\,L^{-1} + \varepsilon ^{1/5}. \end{aligned}$$

Optimizing over *L* gives $L=\varepsilon ^{-1/30} \leqslant K$, which proves (2.14).

Finally, regarding (2.16), let ${\widetilde{\Psi }}_t^\varepsilon :=e^{-i \varepsilon ^{-2} t H^\varepsilon +i\varepsilon ^{-2}\mu (t)}\Psi ^\varepsilon _K(\varphi _0)$ and ${\widetilde{\gamma }}_{\textrm{particle}}^\varepsilon (t):= \operatorname {Tr}_{{\mathcal {F}}} |{\widetilde{\Psi }}_t^\varepsilon \rangle \langle {\widetilde{\Psi }}_t^\varepsilon |$, and ${\widetilde{\Phi }}_t = \psi _{\varphi _t} \otimes {\mathbb {U}}(t) \Omega$. Proceeding as in (8.1), we estimate

$$\begin{aligned} \text {Tr}_{L^2} | {\widetilde{\gamma }}_{\textrm{particle}}^\varepsilon (t) - |\psi _t \rangle \langle \psi _t | |&\le 2 \Vert {\widetilde{\Psi }}_t^\varepsilon - W(\varepsilon ^{-1} \varphi _t ){\widetilde{\Phi }}_t \Vert \le 2 \Vert {\widetilde{\Psi }}_t^\varepsilon - W(\varepsilon ^{-1} \varphi _t )(U_K^\varepsilon )^* {\widetilde{\Phi }}_t \Vert + C \varepsilon K^{-1} \\&= 2\Vert \delta (t) \Vert +C \varepsilon K^{-1}\leqslant C \varepsilon ^{1/10} \ln \tfrac{1}{\varepsilon } , \end{aligned}$$

where we have used (7.8) to control

$$\begin{aligned} \Vert W(\varepsilon ^{-1} \varphi _t ){\widetilde{\Phi }}_t - W(\varepsilon ^{-1} \varphi _t )(U_K^\varepsilon )^*{\widetilde{\Phi }}_t \Vert =\Vert ((U_K^\varepsilon )^*-1) {\widetilde{\Phi }}_t\Vert \lesssim \varepsilon K^{-1} \end{aligned}$$

and the bound on δ(t) from (7.76). □

## Data Availability

No data are associated with this article.

## Renormalization and dressing identity

To keep the paper self-contained, we briefly present the proofs of Proposition 1.1 and Lemma 4.1 for the Nelson Hamiltonian with semiclassical scaling. Since these follow well-known arguments [16], we only sketch the main steps.

### Proof of Proposition 1.1

We introduce the unitary dressing transformation (2.6) with ultraviolet cutoff $\Lambda \geqslant K$:

A.1 $$\begin{aligned} U_{K,\Lambda }^\varepsilon :=\exp \!\Big (\varepsilon \,a^*({\boldsymbol{B}}_{K,\Lambda })-\varepsilon \,a({\boldsymbol{B}}_{K,\Lambda })\Big ), \qquad {\boldsymbol{B}}_{K,\Lambda }(x,k):={\boldsymbol{B}}_K(x,k)\,{\textbf{1}}_{\{|k|\leqslant \Lambda \}} . \end{aligned}$$

Conjugating each term of the regularized Hamiltonian (1.9) yields

A.2 $$\begin{aligned} U_{K,\Lambda }^\varepsilon \,p^2\,(U_{K,\Lambda }^\varepsilon )^*&=p^2+\varepsilon \!\left( 2 a^*(k{\boldsymbol{B}}_{K,\Lambda })\cdot p +2 p \cdot a(k{\boldsymbol{B}}_{K,\Lambda })-\phi (k^2{\boldsymbol{B}}_{K,\Lambda })\right) \nonumber \\&\hspace{-1.8cm} +\varepsilon ^2 \left( 2\,a^*(k{\boldsymbol{B}}_{K,\Lambda })a(k{\boldsymbol{B}}_{K,\Lambda }) +a(k{\boldsymbol{B}}_{K,\Lambda })^2+a^*(k{\boldsymbol{B}}_{K,\Lambda })^2+\Vert kB_{K,\Lambda }\Vert _{L^2}^2\right) , \end{aligned}$$

A.3 $$\begin{aligned} U_{K,\Lambda }^\varepsilon \,(\varepsilon ^2 H_f)\,(U_{K,\Lambda }^\varepsilon )^*&=\varepsilon ^2 H_f-\varepsilon ^3\,\phi (\omega {\boldsymbol{B}}_{K,\Lambda }) +\varepsilon ^4\,\Vert \omega ^{1/2}B_{K,\Lambda }\Vert _{L^2}^2, \end{aligned}$$

A.4 $$\begin{aligned} U_{K,\Lambda }^\varepsilon \,\big (\varepsilon \,\phi ({\boldsymbol{G}}_\Lambda )\big )\,(U_{K,\Lambda }^\varepsilon )^*&=\varepsilon \,\phi ({\boldsymbol{G}}_\Lambda )-2\,\varepsilon ^2\,\text {Re}\langle {\boldsymbol{G}}_\Lambda ,{\boldsymbol{B}}_{K,\Lambda }\rangle . \end{aligned}$$

The scalar $\varepsilon ^2$-terms combine to

A.5 $$\begin{aligned} \varepsilon ^2\Vert kB_{K,\Lambda }\Vert _{L^2}^2-2\varepsilon ^2\text {Re}\langle {\boldsymbol{G}}_\Lambda ,{\boldsymbol{B}}_{K,\Lambda }\rangle =4\pi \varepsilon ^2(\ln K-\ln \Lambda ), \end{aligned}$$

while the field operator terms linear in ε add up to $\phi ({\boldsymbol{G}}_K)$:

A.6 $$\begin{aligned} \phi ({\boldsymbol{G}}_\Lambda )-\phi (k^2{\boldsymbol{B}}_{K,\Lambda })=\phi ({\boldsymbol{G}}_K). \end{aligned}$$

Adding $4\pi \varepsilon ^2\ln \Lambda$ removes the divergent part, and we obtain the dressed regularized operator

A.7 $$\begin{aligned} H_{{\mathscr {D}},K,\Lambda }^\varepsilon&:=U_{K,\Lambda }^\varepsilon \Big (H_\Lambda ^\varepsilon +4\pi \varepsilon ^2\ln \Lambda \Big )(U_{K,\Lambda }^\varepsilon )^*\nonumber \\&=p^2+\varepsilon \Big (\phi ({\boldsymbol{G}}_K)+2\,a^*(k{\boldsymbol{B}}_{K,\Lambda }) \cdot p+2\,p \cdot a(k{\boldsymbol{B}}_{K,\Lambda })\Big ) \nonumber \\&\quad +\varepsilon ^2\Big (H_f+2\,a^*(k{\boldsymbol{B}}_{K,\Lambda })a(k{\boldsymbol{B}}_{K,\Lambda }) +a(k{\boldsymbol{B}}_{K,\Lambda })^2+a^*(k{\boldsymbol{B}}_{K,\Lambda })^2 +4\pi \ln K\Big ) \nonumber \\&\quad -\varepsilon ^3\,\phi (\omega {\boldsymbol{B}}_{K,\Lambda }) +\varepsilon ^4\,\Vert \omega ^{1/2}B_{K,\Lambda }\Vert _{L^2}^2. \end{aligned}$$

Using the bounds (3.24a)–(3.24d) (which apply to $B_{K,\Lambda }$ as well), for any δ>0 there exist $C_\delta ,K_0>0$ such that for all $\Lambda \geqslant K\geqslant K_0$ and ε>0,

A.8 $$\begin{aligned} \pm \big (H_{{\mathscr {D}},K,\Lambda }^\varepsilon -p^2-\varepsilon ^2H_f\big ) \;\leqslant \;\delta \,(p^2+\varepsilon ^2 H_f)+C_\delta K \end{aligned}$$

as quadratic forms. By the KLMN theorem, $H_{{\mathscr {D}},K,\Lambda }^\varepsilon$ is thus self-adjoint and semibounded, with form domain $Q(p^2+H_f)$.

Moreover, following [16, Lem. 3.2] and using again (3.24a)–(3.24d), there exist constants C>0 and $\Lambda _0\geqslant K_0$ such that, for all $\Lambda _2\geqslant \Lambda _1\geqslant \Lambda _0$ and $K\geqslant K_0$,

A.9 $$\begin{aligned} \pm \big (H_{{\mathscr {D}},K,\Lambda _2}^\varepsilon -H_{{\mathscr {D}},K,\Lambda _1}^\varepsilon \big ) \;\leqslant \; C\,\Lambda _1^{-1/4}\,(p^2+\varepsilon ^2 H_f). \end{aligned}$$

By [16, Thm. A.1] there exists a self-adjoint, semibounded operator $H_{{\mathscr {D}},K}^\varepsilon :=H_{{\mathscr {D}},K,\infty }^\varepsilon$ with form domain $Q(p^2+H_f)$ such that

A.10 $$\begin{aligned} s - \lim _{\Lambda \rightarrow \infty } e^{-it H_{{\mathscr {D}},K,\Lambda }^\varepsilon } = e^{-it H_{{\mathscr {D}},K}^\varepsilon } \qquad (\forall t\in {\mathbb {R}}). \end{aligned}$$

Since $U_{K,\Lambda }^\varepsilon \xrightarrow [\Lambda \rightarrow \infty ]{}U_{K,\infty }^\varepsilon$ in the strong sense, we define

A.11 $$\begin{aligned} H^\varepsilon :=(U_{K,\infty }^\varepsilon )^*\,H_{{\mathscr {D}},K}^\varepsilon \,U_{K,\infty }^\varepsilon , \end{aligned}$$

which satisfies

A.12 $$\begin{aligned} s-\lim _{\Lambda \rightarrow \infty } e^{-it H_\Lambda ^\varepsilon }\,e^{-it\,4\pi \varepsilon ^2\ln \Lambda } = e^{-it H^\varepsilon }\qquad (t\in {\mathbb {R}}). \end{aligned}$$

This proves Proposition 1.1. □

### Proof of Lemma 4.1

For $K\geqslant K_0$, the dressing identity in Lemma 4.1 is exactly (A.11). The energy bounds (4.3)–(4.4) follow directly from (3.24a)–(3.24d). □

## Global existence of aKG near minimizers

In this appendix we prove Lemma 1.3 on global well-posedness of the aKG dynamics for initial data close to the manifold of minimizers of the field energy. Recall the field functional

B.1 $$\begin{aligned} {\mathcal {E}}_{\textrm{field}}(u) := e(u) + \Vert u\Vert _{{\mathfrak {h}}_{1/2}}^2, \end{aligned}$$

with minimal value $E_*={\mathcal {E}}_{\textrm{field}}(\varphi _*)$ attained by minimizers $\varphi _*=-\omega ^{-1}\sigma (\psi _*)$, where $\psi _*$ is a minimizer of the Pekar functional (known to be unique up to translations [31]),

B.2 $$\begin{aligned} {\mathcal {E}}_{\textrm{P}}(\psi ) = \langle \psi , p^2 \psi \rangle - \Vert \omega ^{-1/2} \sigma (\psi ) \Vert _{L^2}^2. \end{aligned}$$

The manifold of field minimizers is

B.3 $$\begin{aligned} {\mathcal {M}} := \{\,T_y\varphi _*: y\in {\mathbb {R}}^3\,\},\qquad (T_y u)(k):=e^{-ik\cdot y}u(k). \end{aligned}$$

The proof of Lemma 1.3 is based on the following coercivity estimate for the field functional.

### Lemma B.1

There exists c>0 such that

B.4 $$\begin{aligned} {\mathcal {E}}_{\textrm{field}}(u)-E_* \;\geqslant \; c\ \textrm{dist}_{{\mathfrak {h}}_{1/2}}(u,{\mathcal {M}})^2 \qquad \forall \,u\in {\mathfrak {h}}_{1/2}. \end{aligned}$$

This follows from the known coercivity of the Pekar functional (modulo translations and phase, see [10]). We omit the proof.

### Proof of Lemma 1.3

We start by noting that for any $u\in {\mathfrak {h}}_{1/2}$ and normalized $\psi \in H^1$, the variational principle gives

B.5 $$\begin{aligned} {\mathcal {E}}_{\textrm{field}}(u)\;\leqslant \;{\mathcal {E}}_{\mathrm P}(\psi ) + \Vert \omega ^{1/2}u+\omega ^{-1/2}\sigma (\psi )\Vert _{L^2}^2. \end{aligned}$$

Choosing $\psi =T_y\psi _*$ and recalling $\varphi _*=-\omega ^{-1}\sigma (\psi _*)$ yields

B.6 $$\begin{aligned} {\mathcal {E}}_{\textrm{field}}(u)-E_* \;\leqslant \; \Vert u-T_y\varphi _*\Vert _{{\mathfrak {h}}_{1/2}}^2, \end{aligned}$$

and taking the infimum over *y*,

B.7 $$\begin{aligned} {\mathcal {E}}_{\textrm{field}}(u)-E_* \;\leqslant \;\textrm{dist}_{{\mathfrak {h}}_{1/2}}(u,{\mathcal {M}})^2. \end{aligned}$$

Now, let $\varphi _t$ be the aKG solution with initial data $\varphi _0$ on the maximal interval $(-T_-,T_+)$. We recall conservation of the energy from (5.34),

B.8 $$\begin{aligned} {\mathcal {E}}_{\textrm{field}}(\varphi _t)&= {\mathcal {E}}_{\textrm{field}}(\varphi _0) \quad \text {for all} \quad t\in (-T_-,T_+). \end{aligned}$$

Combining (B.4) with energy conservation and (B.6), we thus obtain

B.9 $$\begin{aligned} \textrm{dist}_{{\mathfrak {h}}_{1/2}}(\varphi _t,{\mathcal {M}})^2&\leqslant \; c^{-1}\big ({\mathcal {E}}_{\textrm{field}}(\varphi _t)-E_*\big ) \nonumber \\&= \; c^{-1}\big ({\mathcal {E}}_{\textrm{field}}(\varphi _0)-E_*\big ) \nonumber \\&\leqslant \; c^{-1}\,\textrm{dist}_{{\mathfrak {h}}_{1/2}}(\varphi _0,{\mathcal {M}})^2, \end{aligned}$$

for all $$t\in (-T_-,T_+)$$.

By assumption, we have $\textrm{dist}_{{\mathfrak {h}}_{1/2}}(\varphi _0,{\mathcal {M}})\leqslant \eta$. Thus $\varphi _t$ remains in a fixed neighborhood of ${\mathcal {M}}$ for all $t\in (-T_-,T_+)$, meaning that for every such *t* there exists $\varphi _*\in {\mathcal {M}}$ with

B.10 $$\begin{aligned} \Vert \varphi _t - \varphi _*\Vert _{{\mathfrak {h}}_{1/2}} \;\leqslant \; c^{-1}\eta . \end{aligned}$$

Since the spectral gap is the same for all $\varphi _*\in {\mathcal {M}}$ and positive, $\triangle (\varphi _*)>0$, we conclude (for sufficiently small η and by Lemma 5.1) that

B.11 $$\begin{aligned} \triangle (\varphi _t)\ \geqslant \ \tfrac{1}{2} \,\triangle (\varphi _*)\ >\ 0 \qquad \forall \,t\in (-T_-,T_+). \end{aligned}$$

If $T_+<\infty$, then $\liminf _{t\rightarrow T_+}\triangle (\varphi _t) >0$, contradicting the continuation alternative from Proposition 1.2. Hence $T_+=\infty$. The same argument shows $T_-=\infty$, and thus the aKG solution with initial datum sufficiently close to ${\mathcal {M}}$ is global. □

## Convergence of SKG to aKG as $\varepsilon \rightarrow 0$

We begin by recalling well-posedness results for the SKG system. Global well-posedness of (SKG) with ε=1 was proved in [37, Thm. 1.4] for initial data $(\psi _0,\varphi _0)\in H^s\times {\mathfrak {h}}_{s-1/2}$ with s⩾0. The solutions conserve mass, $\Vert \psi _t\Vert _{L^2}=\Vert \psi _0\Vert _{L^2}$, and, for s⩾1, also conserve the semiclassical energy ${\mathcal {E}}(\psi _t,\varphi _t)={\mathcal {E}}(\psi _0,\varphi _0)$ defined in (1.11). Moreover, if $(\psi _0,\varphi _0)\in H^1\times {\mathfrak {h}}_{1/2}$, then

C.1 $$\begin{aligned} \sup _{t\in {\mathbb {R}}}\big (\Vert \psi _t\Vert _{H^1}+\Vert \varphi _t\Vert _{{\mathfrak {h}}_{1/2}}\big )<\infty . \end{aligned}$$

For general semiclassical parameter ε>0, global well-posedness in $H^1\times {\mathfrak {h}}_{1/2}$, together with the same conservation laws and uniform $H^1\times {\mathfrak {h}}_{1/2}$ bounds (uniform in ε>0 and $t\in {\mathbb {R}}$), follows by adapting the analysis of the Landau–Pekar equations in [11, Lemma 2.1].

In the next lemma we show that, on the slow time scale $t={\mathcal {O}}(\varepsilon ^{-2})$, SKG solutions converge to the aKG solution pair.

### Lemma C.1

Let $\varphi _0\in {\mathfrak {h}}_0 \cap {\mathfrak {h}}_{1/2}$ with $e(\varphi _0)<0$. Let $\varphi _t$ be the solution to (aKG) with initial datum $\varphi _0$ and let $\psi _{\varphi _t}\geqslant 0$ denote the normalized non-negative ground state of $h_{\varphi _t}$. Moreover, let $(\psi _t^\varepsilon , \varphi _t^\varepsilon )$ denote the solutions to (SKG) for initial data $(\psi _{\varphi _0}, \varphi _0)$. Then there exist $C,T,\varepsilon _0>0$, such that

$$\begin{aligned} \Big \Vert \exp \left( i \varepsilon ^{-2} \int _0^t e \left( \varphi ^\varepsilon _{\varepsilon ^{-2}s} \right) \, \text {d}s \right) \psi ^{\varepsilon }_{\varepsilon ^{-2}t} - \psi _{\varphi _t}\Big \Vert _{L^2}+\, \Big \Vert \varphi ^{\varepsilon }_{\varepsilon ^{-2}t}-\varphi _t \Big \Vert _{L^2} \le C \varepsilon ^{2/7} \end{aligned}$$

for all |t|⩽T and $\varepsilon \in (0,\varepsilon _0]$, where $e(\varphi ^\varepsilon _s)= \inf \sigma (h_{\varphi ^\varepsilon _s})$.

### Proof

We denote the SKG solutions on the slow time scale by $u_t^\varepsilon := \psi ^\varepsilon _{\varepsilon ^{-2}t}$ and $w_t^\varepsilon := \varphi _{\varepsilon ^{-2} t}^\varepsilon$ and recall that they satisfy

C.2 $$\begin{aligned} i\partial _t u_t^\varepsilon = \varepsilon ^{-2} h_{w_t^\varepsilon } u_t^\varepsilon , \qquad \qquad i\partial _t w_t^\varepsilon = \omega w_t^\varepsilon + \sigma (u_t^\varepsilon ). \end{aligned}$$

For the field we estimate

C.3 $$\begin{aligned} \Vert w_t^\varepsilon - \varphi _0 \Vert _{{\mathfrak {h}}_{1/2}} \leqslant \Vert (e^{-i\omega t}-1)\varphi _0\Vert _{{\mathfrak {h}}_{1/2}} + \int _0^{|t|} \Vert \sigma (u_s^\varepsilon ) \Vert _{{\mathfrak {h}}_{1/2}}\,\text {d}s\, , \end{aligned}$$

and use that the first term approaches zero as |t|→0 and that the second term is bounded by a constant times *T* (use Lemma 3.8 together with $\sup _{\varepsilon >0} \sup _{ t\in {\mathbb {R}} } \Vert u_t^\varepsilon \Vert _{H^1}\leqslant C$).

Therefore, by Lemma 5.1, there exists T>0 such that the spectral gap associated with $w_t^\varepsilon$ remains positive, i.e. $\inf _{\varepsilon>0} \inf _{|t|\leqslant T}\triangle (w_t^\varepsilon )>0$. In particular, for every |t|⩽T the operator $h_{w_t^\varepsilon }$ has a unique non-negative ground state $\psi _{w_t^\varepsilon }$ with eigenvalue $e(w_t^\varepsilon )<0$.

The ground state approximates the particle component of the SKG solution via

C.4 $$\begin{aligned} \Big \Vert e^{ i \varepsilon ^{-2} \int _0^t e(w_s^\varepsilon )\,\text {d}s } u_t^\varepsilon - \psi _{w_t^\varepsilon }\Big \Vert _{L^2} \leqslant C\varepsilon ^{2/7}, \qquad |t|\leqslant T. \end{aligned}$$

This can be interpreted as a nonlinear adiabatic theorem for the SKG dynamics, and its proof follows the same strategy as the nonlinear adiabatic theorem for the Landau–Pekar equations in [30, Thm II.1]. For completeness, we sketch the proof of (C.4) at the end of this section.

Moreover, since $\Vert w_t^\varepsilon -\varphi _t\Vert _{{\mathfrak {h}}_{1/2}}\leqslant CT$, Lemma 5.2 applies to $\psi _{w_t^\varepsilon }\geqslant 0$ and $\psi _{\varphi _t}\geqslant 0$, yielding

C.5 $$\begin{aligned} \Vert \psi _{w^\varepsilon _t} - \psi _{\varphi _t}\Vert _{L^2} \leqslant C \Vert w_t^\varepsilon -\varphi _t\Vert _{L^2}. \end{aligned}$$

Combining this with (C.4) gives

C.6 $$\begin{aligned} \Big \Vert e^{ i \varepsilon ^{-2} \int _0^t e(w_s^\varepsilon )\,\text {d}s } u_t^\varepsilon - \psi _{\varphi _t}\Big \Vert _{L^2} \leqslant C\varepsilon + C \Vert w_t^\varepsilon -\varphi _t\Vert _{L^2}. \end{aligned}$$

Finally, for |t|⩽T we estimate

C.7 $$\begin{aligned} \Vert w_t^\varepsilon -\varphi _t\Vert _{L^2}&\leqslant \int _0^{|t|} \Vert \sigma (u_s^\varepsilon )-\sigma (\psi _{\varphi _s})\Vert _{L^2}\,\text {d}s \nonumber \\&\leqslant C \int _0^{|t|} \Big \Vert e^{ i \varepsilon ^{-2} \int _0^s e(w_r^\varepsilon )\,\text {d}r } u_s^\epsilon - \psi _{\varphi _s}\Big \Vert _{L^2}\,\text {d}s \nonumber \\&\leqslant C \varepsilon ^{2/7} + C\int _0^{|t|}\Vert w_s^\varepsilon -\varphi _s\Vert _{L^2}\,\text {d}s, \end{aligned}$$

where we used that $\sigma (\psi )$ is quadratic in ψ. By Grönwall’s inequality we obtain $\Vert w_t^\varepsilon -\varphi _t\Vert _{L^2}\leqslant C\varepsilon ^{2/7}$, and inserting this into (C.6) proves the claimed bounds. □

### Proof of Inequality (C.4)

We briefly sketch the proof of the adiabatic bound (C.4). Since the spectral gap $\triangle (w_t^\varepsilon )>0$ persists uniformly for |t|⩽T and ε>0, all estimates from Lemma 5.3 apply to $w_t^\varepsilon$. In particular, there exists C>0 such that

C.8 $$\begin{aligned}&\Vert R_{w_t^\varepsilon }\Vert + \Vert \langle p\rangle R_{w_t^\varepsilon }^{1/2}\Vert + \Vert \langle p^2\rangle R_{w_t^\varepsilon }\Vert + \Vert \psi _{w_t^\varepsilon }\Vert _{H^2} \leqslant C, \end{aligned}$$

C.9 $$\begin{aligned}&\Vert \dot{\psi }_{w_t^\varepsilon }\Vert _{L^2} + \Vert \dot{\sigma }(\psi _{w_t^\varepsilon })\Vert _{L^2} + \Vert \dot{R}_{w_t^\varepsilon }\Vert + \Vert \langle p\rangle \dot{R}_{w_t^\varepsilon }\langle p\rangle \Vert \leqslant C , \end{aligned}$$

for all |t|⩽T and ε>0. Here, $R_{w_t^\varepsilon } = Q_{w_t^\varepsilon } (h_{w_t^\varepsilon } - e(w_t^\varepsilon ) )^{-1} Q_{w_t^\varepsilon }$ with $Q_{w_t^\varepsilon } = {{\textbf {1}}} - | \psi _{w_t^\varepsilon } \rangle \langle \psi _{w_t^\varepsilon }|$. Moreover,

C.10 $$\begin{aligned} \partial _t \psi _{w_t^\varepsilon } = R_{w_t^\varepsilon } V_{i\omega w_t^\varepsilon } \psi _{w_t^\varepsilon }. \end{aligned}$$

Define $${\widetilde{u}}_t^\varepsilon := e^{i\int _0^t e(w_s^\varepsilon )\,\text {d}s}\,u_t^\varepsilon $$. Then

C.11 $$\begin{aligned} \Vert {\widetilde{u}}_t^\varepsilon - \psi _{w_t^\varepsilon }\Vert _{L^2}^2&=2\,\text {Im}\int _0^t \langle {\widetilde{u}}_s^\varepsilon ,\;\big (\varepsilon ^{-2}(h_{w_s^\varepsilon }-e(w_s^\varepsilon ))-iR_{w_s}V_{i\omega w_s^\varepsilon }\big ) \psi _{w_s^\varepsilon }\rangle \,\text {d}s \nonumber \\&=-2\,\text {Im}\int _0^t \langle {\widetilde{u}}_s^\varepsilon ,\;R_{w_s^\varepsilon }V_{i\omega w_s^\varepsilon }\psi _{w_s^\varepsilon }\rangle \,\text {d}s , \end{aligned}$$

where we used $h_{w_s^\varepsilon } \psi _{w_s^\varepsilon } = e(w_s^\varepsilon ) \psi _{w_s^\varepsilon }$. The remaining term is estimated analogously to the contribution in ${\mathscr {R}}^{(4)}$ in (7.21d). Decomposing $w_s^\varepsilon =w_{s, L}^\varepsilon +w_{s,>L}^\varepsilon$ with $w_{s,L}^\varepsilon := w_s^\varepsilon {{\textbf {1}}}_{|k|\leqslant L}$, the momentum tail satisfies

C.12 $$\begin{aligned} \bigg | \int _0^t \langle {\widetilde{u}}_s^\varepsilon ,\;R_{w_s^\varepsilon }V_{i\omega w^\varepsilon _{s,>L}}\psi _{w_s^\varepsilon }\rangle \,\text {d}s\bigg |\;\lesssim \;L^{-1/4}. \end{aligned}$$

For the low-momentum part, we need to expand one time via the following identity

C.13 $$\begin{aligned} Q_{w_s^\varepsilon }{\widetilde{u}}_s^\varepsilon =R_{w_s^\varepsilon }(h_{w_s^\varepsilon }-e(w_s^\varepsilon )){\widetilde{u}}_s^\varepsilon =i\varepsilon ^2\, R_{w_s^\varepsilon }\, \partial _s{\widetilde{u}}^\varepsilon _s, \end{aligned}$$

which can be viewed as a classical version of (7.28). Note that we used $i\partial _t {\widetilde{u}}^\varepsilon _t = (h_{w_t^\varepsilon }-e(w_t^\varepsilon )) {\widetilde{u}}^\varepsilon _t$.

After integrating by parts we obtain

C.14 $$\begin{aligned} \Vert {\widetilde{u}}_t^\varepsilon -&\psi _{w_t^\varepsilon }\Vert _{L^2}^2 =-2\,\text {Im}\int _0^t \langle i\partial _s{\widetilde{u}}_s^\varepsilon ,\;R_{w_s^\varepsilon }^2V_{i\omega w_{s,L}^\varepsilon } \psi _{w_s^\varepsilon }\rangle \,\text {d}s \nonumber \\&=2\varepsilon ^2\,\text {Im}\langle {\widetilde{u}}_s,\;R_{w_s^\varepsilon }^2 V_{i\omega w_{s,L}^\varepsilon } \psi _{w_s^\varepsilon }\rangle \Big |_0^t \nonumber \\&\quad +2\varepsilon ^2\,\text {Im}\int _0^t \langle {\widetilde{u}}_s^\varepsilon ,\;(2\dot{R}_{w_s^\varepsilon } R_{w_s^\varepsilon } +\dot{V}_{i\omega w^\varepsilon _{s,L} }+R_{w_s^\varepsilon }^2V_{i\omega w_{s,L}^\varepsilon }R_{w_s^\varepsilon }V_{i\omega w_{s,L}^\varepsilon }) \psi _{w_s^\varepsilon }\rangle \,\text {d}s . \end{aligned}$$

Since these terms are completely analogous to the contributions ${\mathscr {R}}^{(4.32)}$ and ${\mathscr {R}}^{(4.24)}$, we obtain for $L=\varepsilon ^{-8/7}$ that

C.15 $$\begin{aligned} \Vert {\widetilde{u}}_t^\varepsilon - \psi _{w_t^\varepsilon }\Vert _{L^2}^2 \;\lesssim \; L^{-1/4}+\varepsilon ^2L^{3/2} \;\lesssim \;\varepsilon ^{2/7}. \end{aligned}$$

This proves (C.4). □

## Footnotes

1 We write ε in place of the $\varepsilon ^{1/2}$ used in [5] to stay consistent with our notation.
